# Side-Chain Type Ferrocene Macrocycles

**DOI:** 10.1021/prechem.3c00121

**Published:** 2024-03-13

**Authors:** Bin Lan, Jindong Xu, Lingyun Zhu, Xinyu Chen, Hideya Kono, Peihan Wang, Xin Zuo, Jianfeng Yan, Akiko Yagi, Yongshen Zheng, Songhua Chen, Yaofeng Yuan, Kenichiro Itami, Yuanming Li

**Affiliations:** † Key Laboratory of Molecule Synthesis and Function Discovery (Fujian Province University), College of Chemistry, Fuzhou University, Fuzhou 350108, China; ‡ Key Laboratory of Advanced Carbon-Based Functional Materials (Fujian Province University), College of Chemistry, Fuzhou University, Fuzhou 350108, China; § Institute of Transformative Bio-Molecules (WPI-ITbM), Nagoya University, Chikusa, Nagoya 464-8602, Japan; ∥ School of Materials Science and Engineering, National Institute for Advanced Materials, Nankai University, Tianjin 300350, China; ⊥ College of Chemistry and Material Science, Longyan University, Longyan 364012, China

**Keywords:** Ferrocene, Conjugated Macrocycles, Organometallic Macrocycles, Second-Order Nonlinear Optical, Nanoring

## Abstract

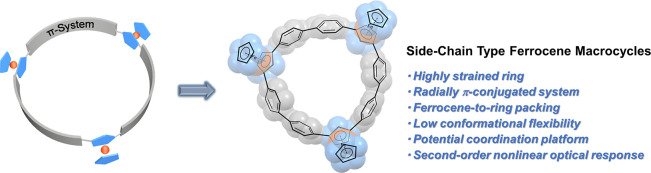

A class of side-chain type ferrocene macrocycles with a radially conjugated system is introduced in this study. The stereo configurations of these ferrocene rings were determined through single-crystal X-ray diffraction analysis. Notably, in the solid state, the ferrocene rings exhibit a distinctive herringbone stacking pattern imposed by a ferrocene-to-ring host–guest interaction. Through UV–vis absorption spectroscopy, electrochemical measurements, and theoretical calculations, valuable insights into the electronic properties of these rings were obtained. In addition, the single crystal of macrocycle **A**_**2**_**B** demonstrates a second-order nonlinear optical response. As a class of organometallic nanorings, this work holds great potential for further exploration in the fields of organometallic chemistry, molecular electronics, and host–guest chemistry.

## Introduction

**Figure 1:**
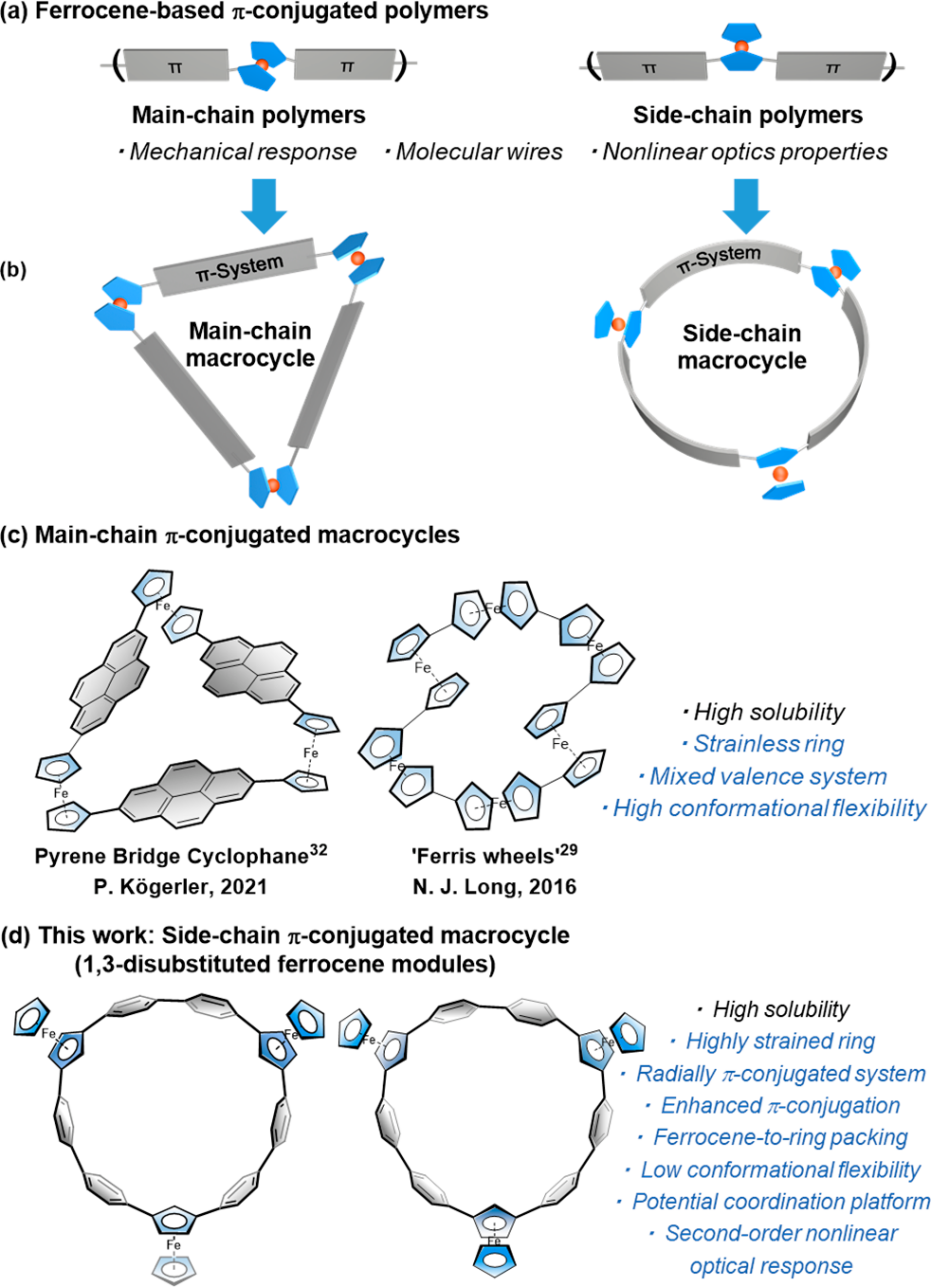
Conceptual representation of (a) ferrocene-based π-conjugated polymers; (b) main-chain and side-chain type ferrocene π-conjugated macrocycles; (c) representative examples of main-chain ferrocene π-conjugated macrocycles; (d) this work: side-chain type radially π-conjugated macrocycles.

Ferrocene-based polymers are attractive materials for potential use in optoelectronic devices (Figure 1a).^1−6^ Symmetrical conjugated cyclic structures not only are aesthetically captivating, but also add unique properties, such as enhanced π-conjugation, solubility, and host–guest interaction, when compared to their noncyclic counterparts.^7−17^ Among these structures, ferrocene-based macrocycles have garnered significant attention in the fields of molecular machinery,^18,19^ molecular electronics,^20−22^ and redox-active supramolecular systems.^23−26^ Drawing inspiration from the paradigm of ferrocene-based polymers,^26−28^ ferrocene-based π-conjugated macrocycles can be categorized into two types: main-chain macrocycles and side-chain macrocycles (Figure 1b). In main-chain macrocycles, the transition metal is an integral component of the macrocycle, whereas in side-chain macrocycles, the transition metal is coordinated to the backbone of the macrocycle.

To date, the main-chain macrocycles incorporating ferrocene units usually feature 1,1′-disubstituted ferrocene units^29,30^ and conjugated linkages, such as pyrene^31,32^ or 1,3-diethynylbenzene^33−35^ (Figure 1c). The inherent rotation of the cyclopentadienyl (Cp) ring in ferrocene likely contributes to the formation of these twisted systems by reducing the strain. These strainless rings exhibit mixed-valence properties, as well as high solubility. We noted that the side-chain macrocyclic species with 1,2-^36^ or 1,3-disubstituted^37,38^ ferrocene modules are much rarer. Macrocycles of this type typically exhibit varied stereoisomerism due to the different orientations of the CpFe group. To fundamentally understand the relationship between organometallic fragment orientation and electronic structure, side-chained ferrocene macrocycles are considered as suitable candidates. In addition, the dissociation of CpFe fragments from the incorporated ferrocenes in side-chain type macrocycles can lead to Cp-embedded macrocycles,^39−41^ which would be a new coordination platform for different metals as a curved π-conjugated macrocyclic ligands.^42,43^ This encouraged us to pursue the synthesis of side-chain type ferrocene rings.

The electrochemical,^25^ aromatic,^30^ and supramolecular properties exhibited by these macrocycles are closely associated with their shape, as well as the number, proximity, and connectivity of their redox centers.^26^ Accordingly, to enrich the functional landscape of side-chain type ferrocene-based conjugated macrocycles, we now introduce two nanosized conjugated macrocycles with different stereoisomerism by incorporating three or four 1,3-ferrocenylene units and paraphenylenes as linkage to give a new class of organometallic rings,^44−52^ which feature radial conjugation, restricted conformation, and enhanced π-conjugation. The unique ferrocene-to-ring host–guest interaction of these macrocycles was determined through single-crystal X-ray diffraction analysis.

## Results and Discussion

### Synthesis

**Scheme 1:**
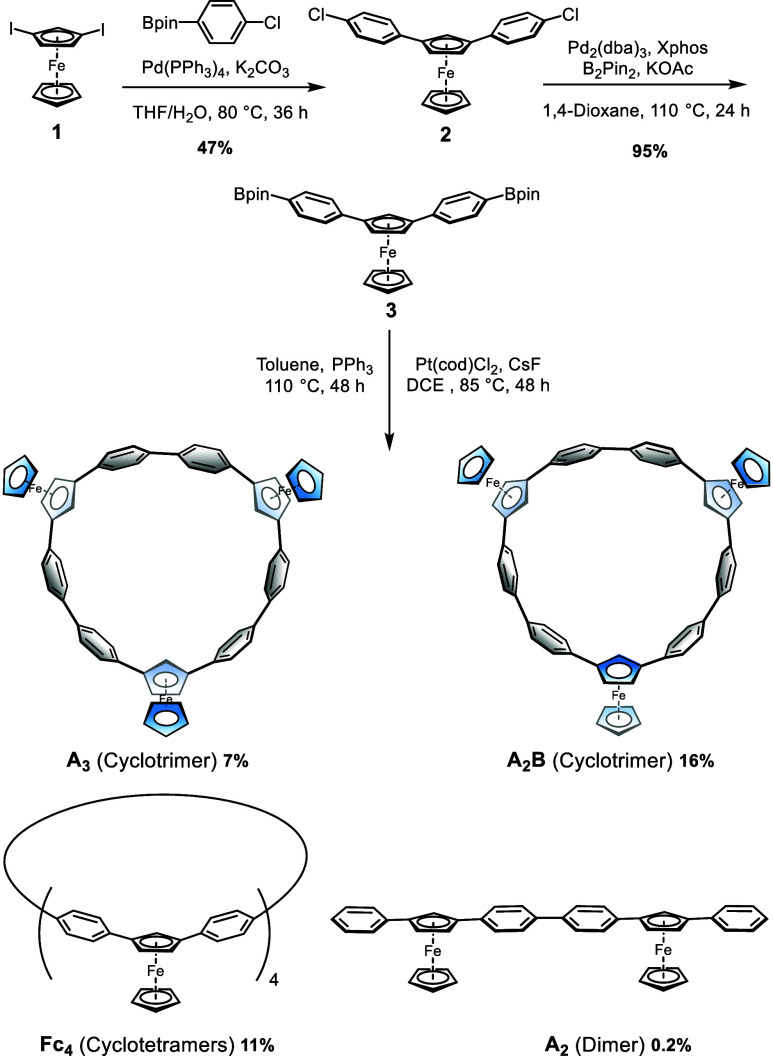
Synthetic Route to Side-Chain Type Ferrocene Rings

The synthesis of the target macrocycles was achieved by the Pt-mediated coupling strategy developed by Bäuerle,^53,54^ Yamago,^55^ and Isobe et al. (Scheme 1).^56,57^ Initially, 1,3-diiodoferrocene **1** was prepared according to the method established by Weissensteiner and co-workers.^58^ Subsequently, the macrocyclic precursor diboronic ester **3** was obtained through the Suzuki–Miyaura coupling reaction, followed by Miyaura borylation in 45% total yield. In addition, crystals of compound **2** were obtained by slow evaporation from a solution of the complex in a CH_2_Cl_2_/*n*-hexane mixture (see Figure S1 for detailed structure information). The transmetalation of compound **3** with Pt(cod)Cl_2_ in 1,2-dichloroethane (DCE) heated at reflux in the presence of cesium fluoride for 48 h afforded the macrocyclic platinum intermediate. Without further purification, the resultant mixture was subjected to reductive elimination conditions to give the target macrocycles, namely, cyclotrimers (**A**_**2**_**B** and **A**_**3**_) and cyclotetramers (**Fc**_**4**_).

As anticipated, the incorporation of 1,3-disubstituted ferrocene into the side-chain type macrocycle introduced geometrical isomerism. For the cyclotrimers, the ratio of two possible stereoisomers, **A**_**2**_**B** and **A**_**3**_, was determined to be 5:2 by using ^1^H NMR spectroscopy (Figure S53). Both stereoisomers could be separated through repeated silica gel column chromatography, yielding **A**_**3**_ in 7% yield and **A**_**2**_**B** in 16% yield. The stereoisomers of the cyclotetramers could not be separated using either silica gel column chromatography or gel permeation chromatography (GPC). However, **Fc**_**4**_ was confirmed by MALDI FT-ICR MS. Additionally, a linear dimer **A**_**2**_ was also isolated in 0.2% yield.

**Figure 2:**
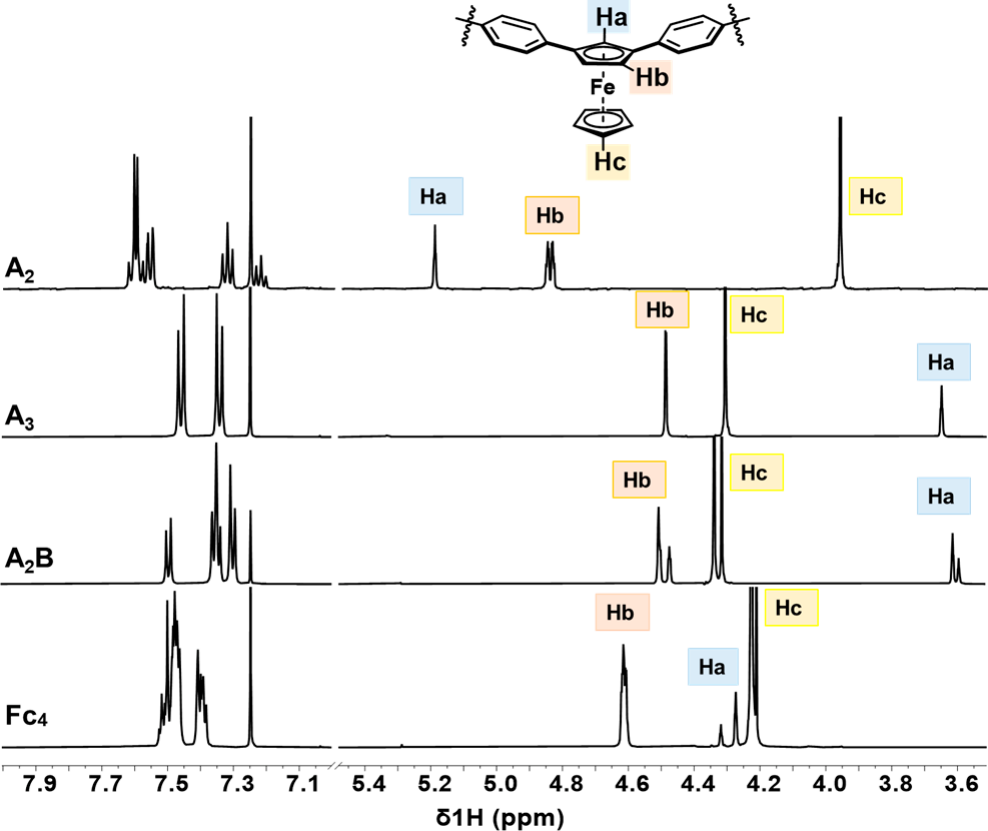
Partial ^1^H NMR spectra of **A**_**2**_, **A**_**3**_, **A**_**2**_**B**, and **Fc**_**4**_ in CDCl_3_.

The ^1^H NMR spectra of **A**_**2**_**B**, **A**_**3**_, **A**_**2**_, and **Fc**_**4**_ were compared to reveal interesting signal shifts of the hydrogen atoms of Cp rings (Figure 2). **A**_**3**_ exhibited a set of ferrocenyl signals, consistent with the theoretically high C_3 V_ symmetry, while **A**_**2**_**B** displayed two sets of signals corresponding to *C*_*S*_ symmetry. Upon comparison with **A**_**2**_, it was observed that the ferrocenyl units in the macrocyclic products exhibited tilting, resulting in progressive upfield shifts of the hydrogen atom signals (H_a_ and H_b_) on Cp embedded in the ring. Specifically, the signal of H_a_ showed a significant shift of 1.54 ppm for **A**_**3**_, 1.58 and 1.60 ppm for **A**_**2**_**B**, respectively. This upfield shift phenomenon is more pronounced than in main-chain type ferrocene rings.^29^ In contrast, the chemical shift of H_c_ shifted downfield as the conformationally flexible Cp rings moved away from the shielding region. Notably, for **Fc**_**4**_, an increasing ring size led to a gradual convergence of the corresponding signals toward those observed in linear congeners.

## X-ray Crystal Structures

**Figure 3:**
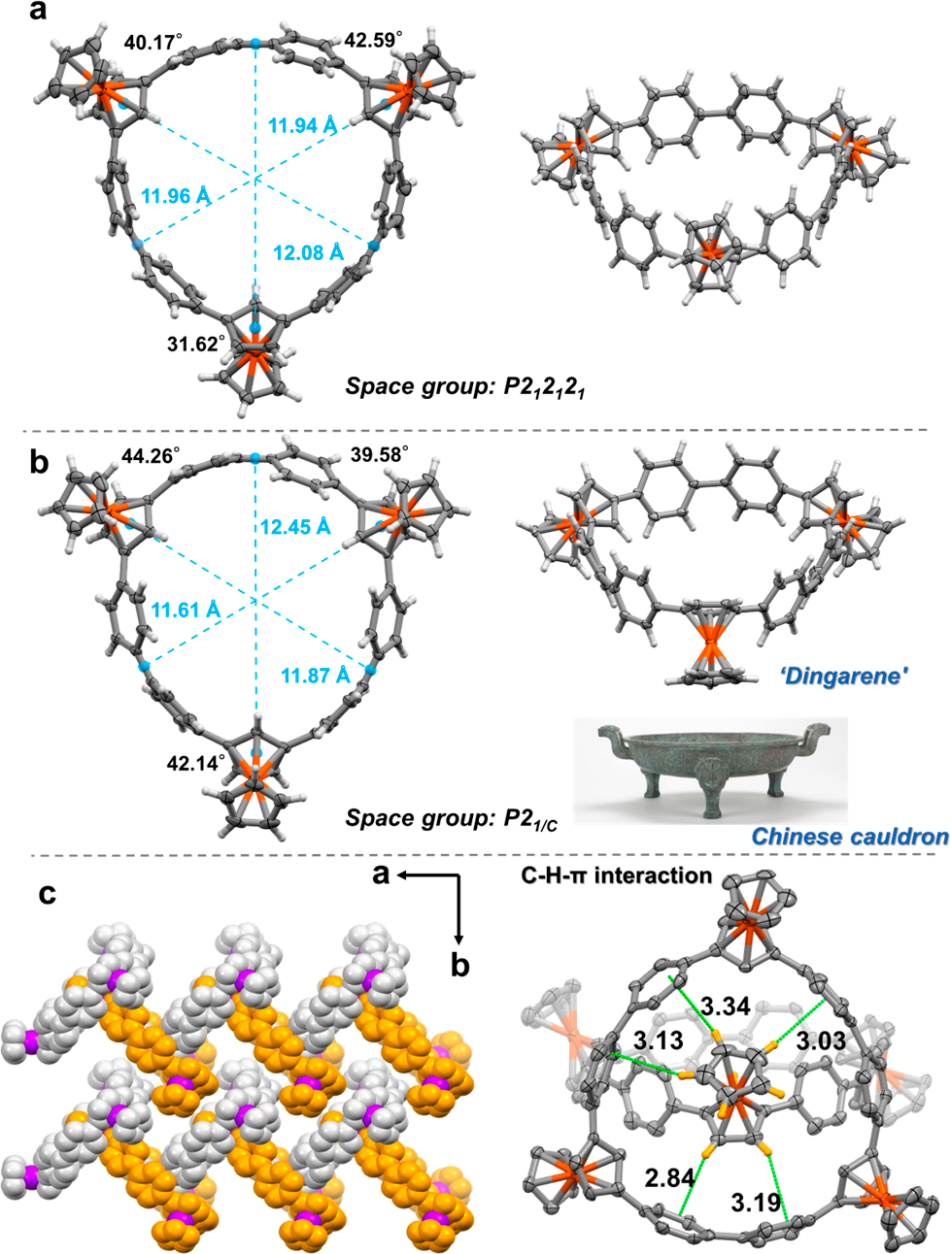
X-ray crystal structures of (a) **A**_**2**_**B** and (b) **A**_**3**_ (thermal ellipsoids are shown at 50% probability; solvent molecules have been omitted for clarity). (c) Crystal stacking along the *a*-axis of **A**_**2**_**B** and distances between the hydrogen atoms and the aromatic plane (iron atoms, purple).

Single crystals of **A**_**2**_**B** and **A**_**3**_, suitable for X-ray diffraction analysis, were obtained by slow diffusion of dichloromethane into an *n*-hexane solution, allowing for the unambiguous determination of the stereoconfiguration of the cyclotrimers. As shown in Figure 3b, all three ferrocenyl units are in the same orientation, with respect to the ring plane in **A**_**3**_. In **A**_**2**_**B**, two ferrocenyl units are in a *syn* orientation with each other, while the third ferrocenyl unit is in an *anti*-orientation relative to them. These distinct configurations result in different space groups, *P*2_1_2_1_2_1_ (**A**_**2**_**B**) and *P*2_1/*c*_ (**A**_**3**_). The average diameter of both macrocycles is approximately 12 Å. In **A**_**2**_**B**, the rotational angle between the macrocycle plane and the embeded Fc plane is 31.62° for the *anti* ferrocenyl unit, while for the *syn* ferrocenyl units, the angles are 40.17° and 42.59°, respectively. In the case of **A**_**3**_, these values are close, which are 44.26°, 39.58°, and 42.14°, respectively (see Figures S5 and S10 for detailed measurement reference information). Regarding the tilting of FeCp groups, the angles are 3.49°, 2.56° and 1.68° for **A**_**3**_, and 3.69° (anti), 1.52° (syn) and 0.62° (syn) for **A**_**2**_**B**. Due to tripod-like shape of **A**_**3**_, reminiscent of ancient Chinese cauldrons, ceremonial vessels known as “ding”, **A**_**3**_ was aptly named “dingarene”.

Furthermore, both **A**_**2**_**B** (Figure 3c) and **A**_**3**_ (Figure S9) displayed a distinctive herringbone stacking pattern imposed by a ferrocene-to-ring host–guest interaction. The ferrocenyl unit in each ring is effectively nestled within the cavity of the neighboring rings, primarily driven by Cp-H-π interactions. In the case of **A**_**2**_**B** (**A**_**3**_ see Figure S12), the distance between hydrogen atoms and aromatic planes (*d*_PLN_), the distance between the carbon atoms and the center of the aromatic planes (*d*_C–X_), and the angle at the hydrogen (∠C–H–X) (X represents the centroid of the phenyl) are measured to be 3.1 4.0, and 147.2°, respectively. These values align perfectly with the established C–H−π criteria, demonstrating the presence of these interactions.^59,60^ Notably, this observation is consistent with the host–guest complexes of Fc⊂[8]cycloparaphenylenes (CPP) that have been recently reported.^61^

## Redox and Photophysical Properties

**Figure 4:**
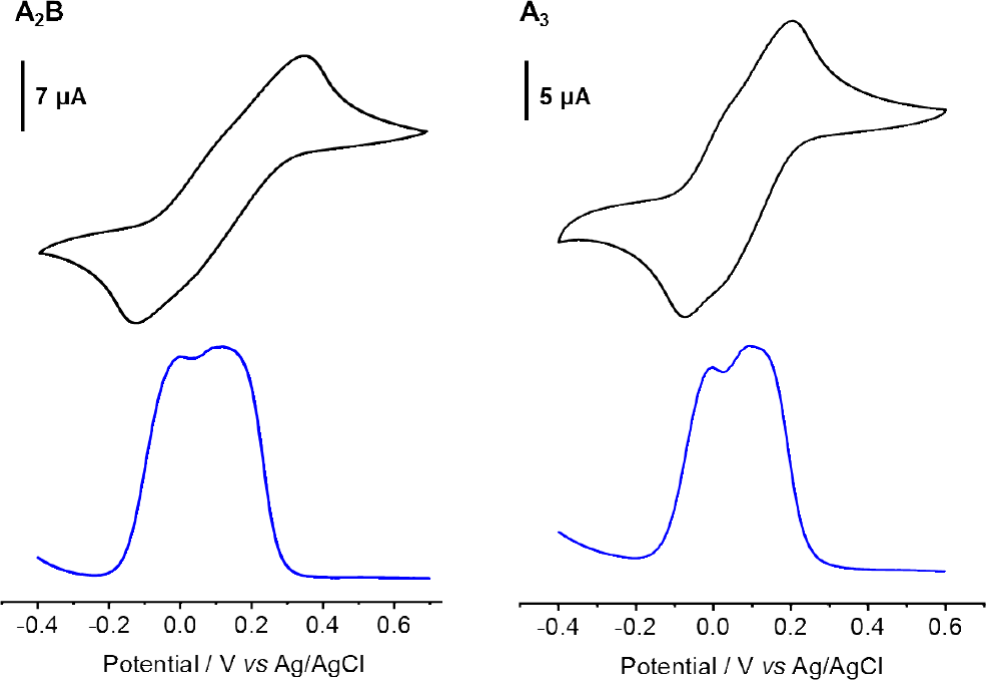
Cyclic voltammetry (CV, top) and differential pulse voltammetry (DPV, bottom) were recorded in 0.01 M [Na][BArF]/CH_2_Cl_2_.

For multiferrocenyl cyclic systems, electrochemistry serves as a conventional tool for assessing the electronic communication between redox sites.^62,63^ In this regard, the redox properties of **A**_**2**_**B** and **A**_**3**_ were investigated by using cyclic voltammetry (CV) and differential pulse voltammetry (DPV). Initially, the CV of **A**_**2**_**B** and **A**_**3**_ in 0.1 M [*n*-Bu_4_N][PF_6_]/CH_2_Cl_2_ were examined, revealing a single reversible redox wave for both compounds (Figure S13). In an attempt to enhance the splitting of the redox potentials, we substituted the electrolyte with [*n*-Bu_4_N][BArF] (BArF = tetrakis[3,5-bis(trifluoromethyl)phenyl]borate), as BArF exhibits a weak ion-pairing ability and increased electrostatic interaction.^64,65^ However, this modification also resulted in only one unresolved wave in the CV (Figure S14). Finally, by further replacing the cations with Na^+^, two unresolved waves were observed in 0.01 M [Na][BArF]/CH_2_Cl_2_ (Figure 4).^66,67^ The lack of a clear splitting between the oxidation waves suggests weak electronic communication between ferrocenyl units,^68,69^ which is likely due to the large iron–iron geometric distance.^70^ The lack of electronic interactions was verified further by the absence of any detectable intervalence charge transfer band in redox titrations (Figures S32 and S33).

**Figure 5:**
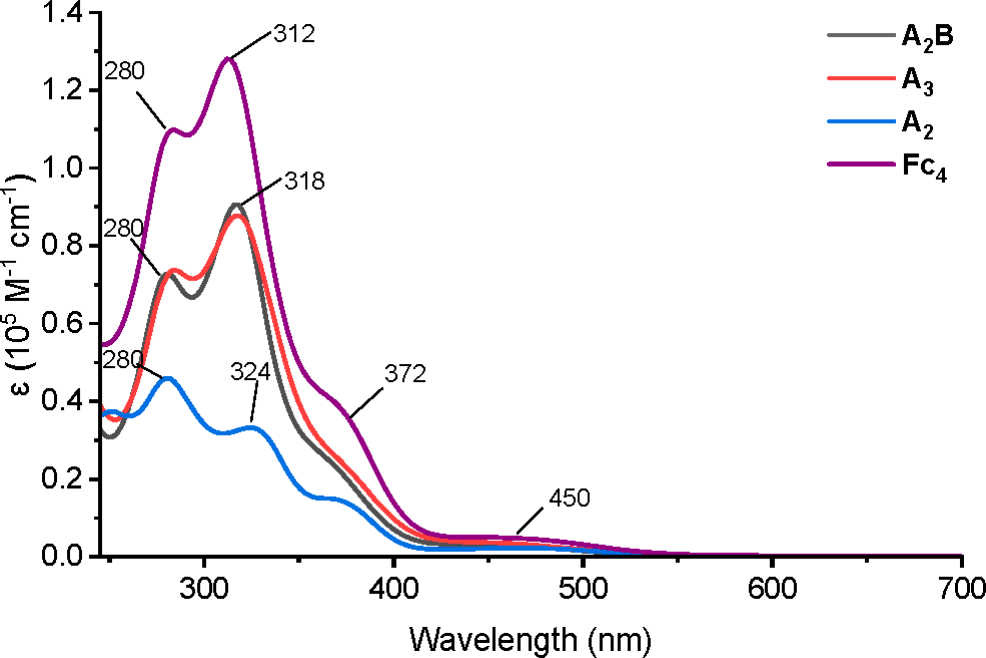
UV–vis absorption spectra of **A**_**2**_**B**, **A**_**3**_, **A**_**2**_, and **Fc**_**4**_ recorded in (10^–5^–10^–6^ M) CH_2_Cl_2_.

The UV–vis absorption spectra of **A**_**2**_, **A**_**2**_**B** and **A**_**3**_ recorded in CH_2_Cl_2_ are shown in Figure 5. **A**_**2**_**B** exhibits a maximum absorption at 318 nm and a weaker absorption at 280 nm, which is similar to **A**_**2**_ but with higher intensity observed. **A**_**3**_ displays a highly overlapping absorption spectrum with that of **A**_**2**_**B**. The dependence of the electronic structure on the *syn*/*anti* relationship of the ferrocenyl fragments appears insignificant, which is in agreement with the reported results for the ferrocene-dehydroannulenes.^36^ To further investigate the ring-size effect, the absorption spectrum of mixture **Fc**_**4**_ was obtained and showed a similar absorption pattern. Additionally, the much weaker absorption bands (around 450 nm) observed in these ferrocenyl compounds were attributed to ferrocene d-d transition.^71,72^ Likely because ferrocene is commonly known as a luminescence quencher, no detectable fluorescence was observed from these four compounds in a CH_2_Cl_2_ solution.

**Figure 6:**
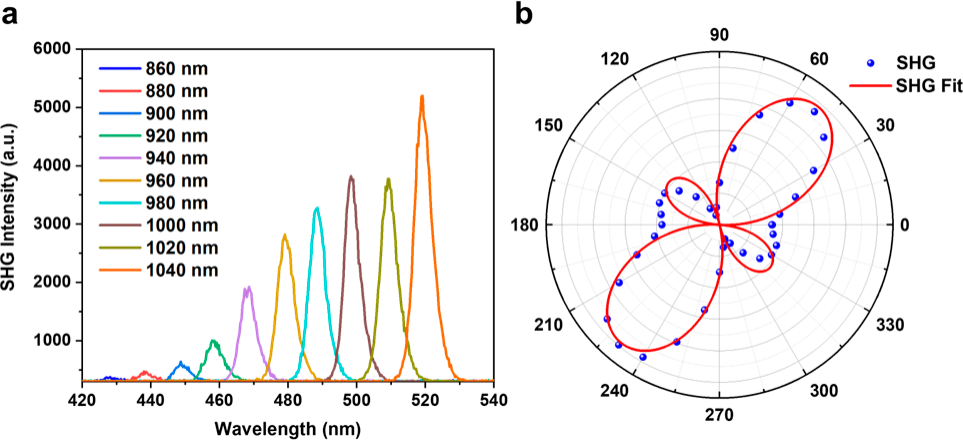
(a) NLO spectra of **A**_**2**_**B** single crystals pumped at different wavelengths from 860 to 1040 nm. (b) Polarization dependence plot of the SHG signal of the **A**_**2**_**B** single crystal with an incident wavelength of 1040 nm.

In addition, we note that **A**_**2**_**B** may have nonlinear optically relevant properties,^73,74^ with the noncentrosymmetric space group (*P*2_1_2_1_2_1_) that is required for second harmonic generation. As the excitation power remains constant, nonlinear optical (NLO) spectra of **A**_**2**_**B** show clear second-harmonic generation (SHG) responses in a broad scope of pump wavelengths swept from 860 to 1040 nm (Figure 6a), with the incidence and detection angles set at 45° with respect to the surface normal. The largest SHG signal can be pumped at approximately 1040 nm for **A**_**2**_**B**. The quadratic correlation plot of the SHG intensity versus the incident laser power for the **A**_**2**_**B** crystal at an 880 nm incident wavelength has a linear fitting slope of 1.99 (Figure S17). Next, the polarization dependence of the NLO signal is investigated (Figure 6b). The maximum value of the SHG response of the crystal occurs when the excitation beam is polarized in directions of 50° and 230°.

## Frontier Molecular Orbitals

**Figure 7:**
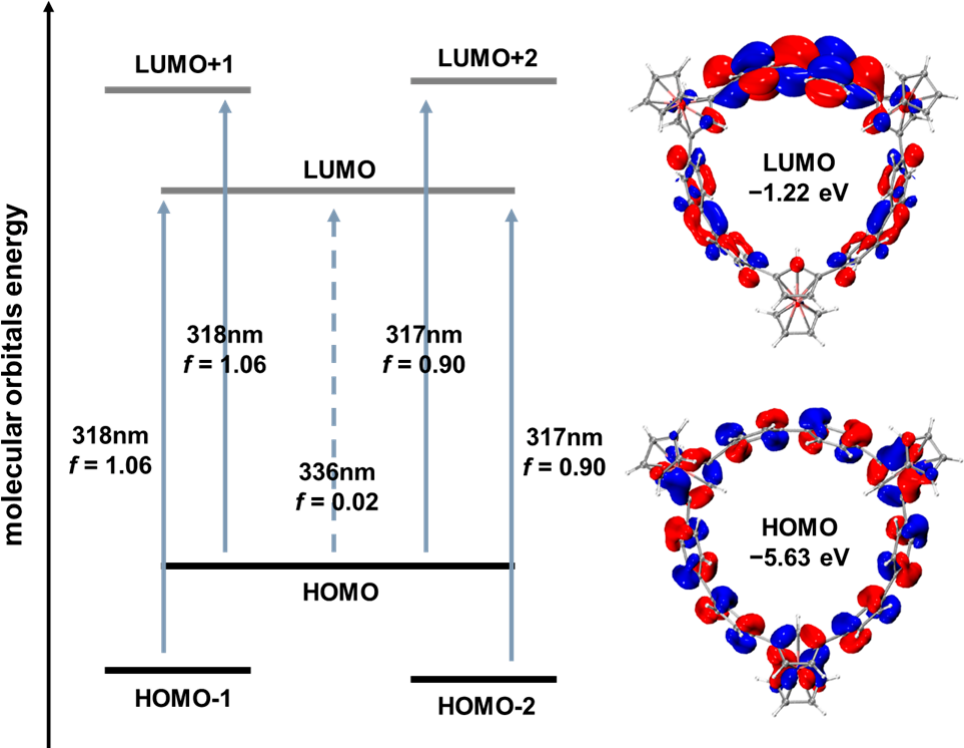
Qualitative energy diagram and frontier molecular orbitals of **A**_**2**_**B**, calculated at the PBE0-D3(BJ)/Def2-SVP level of theory.

To further understand the effect of the orientation of ferrocenyl units in macrocycles, density functional theory (DFT) calculations were carried out. Geometry optimizations for the structures of **A**_**3**_ and **A**_**2**_**B** were performed at the PBE0-D3(BJ)/Def2-SVP level of theory (Figure S18). It was found that **A**_**2**_**B** is thermodynamically more stable than **A**_**3**_, with a difference of about 2.3 kcal mol^–1^. Furthermore, time-dependent (TD)-DFT calculations were performed at the PBE0-D3(BJ)/Def2-SVP level of theory. **A**_**2**_**B** and **A**_**3**_ exhibit similar orbital distributions (Figures S25 and 26), with the highest occupied molecular orbital (HOMO) delocalized along the entire backbone and the lowest unoccupied molecular orbital (LUMO) predominantly delocalized on the biphenyl moieties, indicating the formation of a π-conjugate system. Both compounds have HOMO levels of −5.63 eV, while the LUMO levels are −1.22 and −1.26 eV for **A**_**2**_**B** and **A**_**3**_, respectively (Table S2). The simulated UV–vis absorption spectra of **A**_**2**_**B** and **A**_**3**_, obtained through TD-DFT, are consistent with the experimental results. In both cases, the HOMO to LUMO transition is forbidden. In addition, the major absorption peaks (318 nm) originate from HOMO–1 to LUMO and HOMO to LUMO+1 transitions (*f* = 1.06), as well as from HOMO–2 to LUMO and HOMO to LUMO+2 (*f* = 0.90) (Figure 7), while the higher energy absorption peak (280 nm) is more complicated (see Tables S3 and S4).

**Figure 8:**
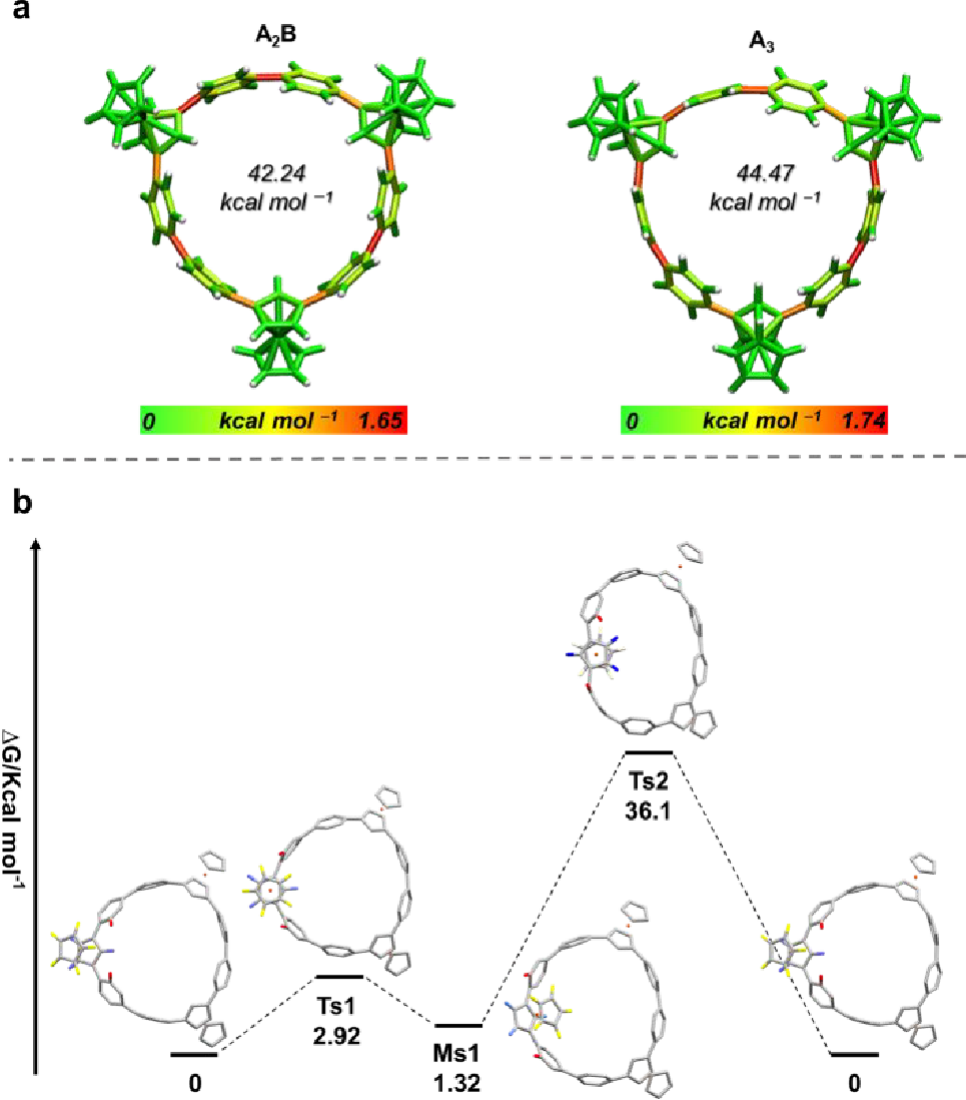
(a) Molecular strain energy for **A**_**2**_**B** and **A**_**3**_ calculated and visualized using StrainViz and (b) relative Gibbs free energy diagram of the ferrocenyl rotational barriers in **A**_**3**_ (B3LYP/SDD, 6-31g(d); Transition State: Ts; Metastable State: Ms.).

The strain energies of **A**_**2**_**B** and **A**_**3**_ were evaluated using homodesmotic reactions,^75^ revealing that **A**_**2**_**B** is slightly less strained (43 kcal mol^–1^) than **A**_**3**_ (45 kcal mol^–1^) (Scheme S1). This finding suggests that the main product being **A**_**2**_**B** is likely due to its lower strain. These values are also much lower than that [9]CPP (66 kcal mol^–1^).^75^ To further analyze the strain distribution (Figure 8a), we employed the StrainViz program developed by Jasti et al.^76^ The results indicate that **A**_**3**_ has more strain than **A**_**2**_**B** in various aspects (Table S6).

In addition, the barriers of flipping three types of ferrocenyl units in **A**_**2**_**B** and **A**_**3**_ were investigated. In all cases, two transition states (Ts) and one metastable state were identified. For example, in **A**_**3**_ (Figure 8b), the energy difference required to transition from the most stable structure to Ms1 is approximately 2.92 kcal mol^–1^. The Ts2, with an energy barrier of 36.1 kcal mol^–1^, is associated with a change in the orientation of the ferrocenyl unit. This energy barrier suggests that the ferrocenyl unit is unlikely to cross at room temperature (see Figure S20–21 for detailed information on the energy barrier for **A**_**2**_**B**).

In terms of future application, some insight into side-chain type ferrocene rings’ coordination organometallic chemistry has been provided. The dissociation of ferrocene in main-chain ferrocene macrocycles leads to ring-opened linear products.^39−41^ However, side-chain ferrocene macrocycles can form macrocycles embedded in Cp, which are more attractive for subsequent modification due to the metal coordination chemistry of the Cp unit. For example, Garbicz et al. recently reported that the Cp unit in side-chain macrocycle acts as a synthon for the construction of meso-tetraaryl-21-carbaporphyrin.^38^ Notably, we observed signals of macrocyclic species shedding CpFe by MALDI FT-ICR MS (Figures S34–S36), which demonstrated that the side-chain ferrocene rings could be promising macrocyclic ligands for exploring organometallic chemistry in a well-defined curved radially conjugated macrocyclic environment.

## Conclusions

In summary, new side-chain type ferrocene-based π-conjugated macrocycles have been successfully synthesized and analyzed in detail. The stereo configuration of the two cyclotrimers was unambiguously confirmed through single crystal X-ray diffraction. Notably, both macrocycles exhibit unique herringbone packing induced by ferrocene-to-ring host–guest interactions, suggesting potential applications in host–guest chemistry. UV–vis absorption spectroscopy and theoretical calculation indicate that the orientation of ferrocenyl units (*syn*/*anti*) has no significant effect on the electronic structure of these cyclic conjugated systems. Electrochemical measurements support these findings and also reveal a weak interaction between the redox centers. **A**_**2**_**B** single crystals demonstrated second-order nonlinear optical properties. In addition, these rings have the potential to generate Cp-containing macrocycles, formed after Cp-FeCp breaking. Further studies are underway to exploit the coordination ability of the Cp-containing macrocycle as a curved π-conjugated macrocyclic ligands. Considering the recent advances in single-molecule electronics with conjugated cyclic structures,^77−79^ the study of electronic transport performance of these new type macrocycles is ongoing as well.

## Supporting Information Available

Experimental procedures and characterization data for all compounds; electrochemistry, NLO measurements, UV–vis-NIR, VT-NMR experimental details, and computational results (PDF)
Crystallographic data for **2** (CIF)
Crystallographic data for **A**_**2**_**B** (CIF)
Crystallographic data for **A**_**3**_ (CIF)

## Supplementary Material

pc3c00121_si_001.pdf

pc3c00121_si_002.cif

pc3c00121_si_003.cif

pc3c00121_si_004.cif
