# Supplementary material for: Side-Chain Type Ferrocene Macrocycles
Source: Precis Chem. 2024 Mar 13;2(4):143–50. doi: 10.1021/prechem.3c00121 (PMC11503920; doi:10.1021/prechem.3c00121)
Supplement: Supplementary file 1 — pc3c00121_si_001.pdf [file pc3c00121_si_001.pdf]

Supporting Information

Side-chain Type Ferrocene Macrocycles

Bin Lan [a], Jindong Xu [a], Lingyun Zhu [a], Xinyu Chen [a], Hideya Kono [c], Peihan Wang [d], Xin Zuo[a], Jianfeng Yan[a], Akiko Yagi[c], Yongshen Zheng[d], Songhua Chen[e]*, Yaofeng Yuan[a]*, Kenichiro Itami[c]* and Yuanming Li[a,b]*

a *Key Laboratory of Molecule Synthesis and Function Discovery (Fujian Province University), College of Chemistry, Fuzhou University, Fuzhou 350108, China.*

b *Key Laboratory of Advanced Carbon-Based Functional Materials (Fujian Province University), College of Chemistry, Fuzhou University, Fuzhou 350108, China.*

c *Institute of Transformative Bio-Molecules (WPI-ITbM), Nagoya University, Chikusa, Nagoya 464-8602 (Japan).*

d *School of Materials Science and Engineering, National Institute for Advanced Materials, Nan-kai University, Tianjin, 300350, China*

e *College of Chemistry and Material Science, Longyan University, Longyan, 364012, China.*

**E-mail: yuanming.li@fzu.edu.cn*

**E-mail: yaofeng_yuan@fzu.edu.cn*

**E-mail: itami@chem.nagoya-u.ac.jp*

**E-mail: songhua@iccas.ac.cn*

Table of Contents

1. General S3

2. Synthetic Procedures S4

2.1 Synthesis of 1,3-Diiodoferrocene (**1**) S4

2.2 Synthesis of 1,3-Bis(4-chlorophenyl)ferrocene (**2**) S4

2.3 Synthesis of 1,3-Bis(4-(4,4,5,5-tetramethyl-1,3,2-dioxaborolan-2- yl)phenyl)ferrocene (**3**) S5

2.4 Synthesis of **A_2B** and **A_3** S5

3. X-Ray Crystallography S7

3.1 Single-crystal Structure and Packing of **2** S8

3.2 Single-crystal Structure and Packing of **A_2B** S9

3.3 Single-crystal Structure and Packing of **A_3** S11

4. Electrochemistry S14

5. NLO measurements S16

6. DFT Calculations S17

6.1 Calculated parameters S17

6.2 Calculated molecular geometries S18

6.3 Rotational Barriers S19

6.4 TD-DFT calculation S21

6.5 Strain energy calculations S23

6.6 Frontier Molecular Orbitals S24

6.7 Cartesian coordinates of optimized structures S27

7. Variable Temperature NMR Spectroscopy S33

8. UV/VIS/NIR SPECTROSCOPY S34

9. References S35

10. Mass Spectra S36

11. NMR Spectra S39

1. General

All reagents and solvents were obtained from commercial suppliers and used without further purification unless noted otherwise. The silica-gel column chromatography was performed using silica gel (200-300 mesh). All reactions were run under argon atmosphere unless otherwise stated. Nuclear magnetic resonance (NMR) spectra were recorded on a JEOL JNM-ECZ500R (^{1}H 500 MHz, ^{13}C 126 MHz) spectrometer and a JEOL JNM-ECZ600R (^{1}H 600 MHz, ^{13}C 151 MHz) spectrometer. Chemical shifts are expressed in parts per million (ppm) relative to tetramethylsilane (δ 0.00 ppm). ^{1}H and ^{13}C NMR spectra are referenced against the residual solvent peak ($CDCl_3$ δ_H = 7.26 ppm, $CDCl_3$ δ_C = 77.16 ppm). The high-resolution mass spectra (HRMS) were conducted on Thermo fisher scientific exactive plus LC-MS (ESI) mass spectrometer. Fourier transform ion cyclotron resonance ultra high resolution mass spectrometer (FTICR-MS) by matrix-assisted laser desorption/ionization (MALDI) were performed Bruker Solarix MRMS. UV–vis absorption spectra were recorded on a PerkinElmer Lambda750 UV-vis/NIR spectrophotometer. Single-crystal X-ray data were collected on a Supernova single-crystal diffractometer equipped with graphite-monochromatized Ga-Kα (λ = 1.34050) at 100 K or on a Bruker Apex diffractometer and 1.5 kW graphite monochromated Mo-Kα (λ = 0.71073) and Cu-Kα radiation (λ = 1.54184 Å) at 298 K. Absorption corrections were applied using SADABS. The structures were solved by the direct method and refined by full-matrix least-squares on F^2 using SHELXTL and OLEX2.[1] The diffused electron densities resulting from these residual cations and solvent molecules were removed from the data set using the SQUEEZE routine of PLATON and refined further using the data generated.[2]

2. Synthetic Procedures

2.1 Synthesis of 1,3-Diiodoferrocene (1)

1,3-Diiodoferrocene was prepared according to previous literature.[3]

2.2 Synthesis of 1,3-Bis(4-chlorophenyl)ferrocene (2)

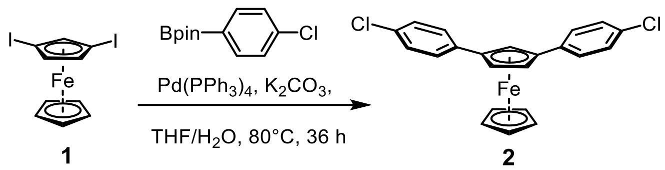

1,3-Diiodoferrocene **1** (1.10 g, 2.51 mmol, 1 equiv.), 4-chlorophenylboronic acid pinacol ester (2.40 g, 10.1 mmol, 4 equiv.), $Pd(PPh_3)_4$ (580 mg, 0.502 mmol, 20 mol%), and K_2CO_3 (3.47 g, 25.1 mmol, 10 equiv.) were added to a 250 mL round-bottom flask equipped with a stir bar. After the flask was evacuated and refilled with argon 3 times, degassed THF (65 mL) and water (25 mL) were transferred to the flask via syringe. The mixture was stirred at 80 °C for 36 h. After cooling down to room temperature, the aqueous layer was extracted with dichloromethane (DCM). The combined organic layers were washed with brine and dried with Na_2SO_4. Solvent was removed under reduced pressure. Purification using silica gel column chromatography (DCM/petroleum ether: 1:50) afforded the compound **2** as a red solid (479 mg, 47%).

Compound **2**
***R**f* **=** 0.55 (DCM/petroleum ether: 1:50)
^{1}H NMR (500 MHz, $CDCl_3$) δ 7.47–7.43 (m, 4H), 7.30–7.27 (m, 4H), 5.08 (t, *J* = 1.5 Hz, 1H), 4.78 (d, *J* = 1.4 Hz, 2H), 3.92 (s, 5H).
^{13}C{^{1}H} NMR (126 MHz, $CDCl_3$) δ 137.5, 131.9, 128.7, 127.3, 85.4, 71.5, 67.6, 65.0.
HRMS (ESI) *m*/*z* calculated for $C_{22}H_{16}Cl_2Fe$ $[M]^+$ 405.9973, Found: 405.9966.

2.3 Synthesis of 1,3-Bis(4-(4,4,5,5-tetramethyl-1,3,2-dioxaborolan-2-yl)phenyl)ferrocene (3)

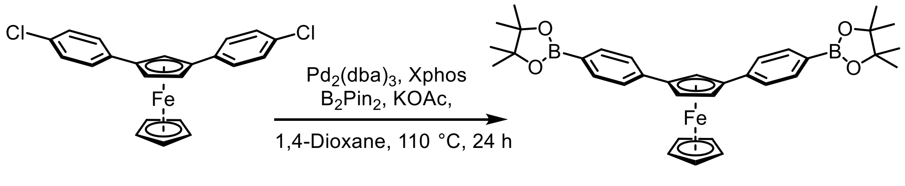

2 3

In a nitrogen-filled glove box, compound **2** (200 mg, 0.491 mmol, 1 equiv.), KOAc (289 mg, 2.95 mmol, 6 equiv.), bis(pinacolato)diboron (749 mg, 2.95 mmol, 6 equiv.), $Pd_2(dba)_3$ (9.00 mg, 9.82 μmol, 2 mol%), XPhos (14.0 mg, 29.5 μmol, 6 mol%) and anhydrous 1,4-dioxane (6 mL) were sequentially added to a Schlenk tube. The tube was then transferred out of glove box. The mixture was stirred at 110 °C for 24 h. After cooling to room temperature, the mixture was filtered through Celite with the aid of DCM (20 mL). The solvent was removed under reduced pressure. The crude product was recrystallized from DCM/MeOH to afford compound **3** as orange solid (275 mg, 95%).

Compound **3**
***R**_f* **=** 0.25 (DCM/petroleum ether: 1:1)
^{1}H NMR (500 MHz, $CDCl_3$) δ 7.76 (d, *J* = 7.7 Hz, 4H), 7.55 (d, *J* = 7.7 Hz, 4H), 5.24 (s, 1H), 4.87 (s, 2H), 3.89 (s, 5H), 1.37 (s, 24H).
^{13}C{^{1}H} NMR (126 MHz, $CDCl_3$) δ 142.3, 135.0, 125.4, 86.0, 83.9, 71.5, 67.9, 65.4, 25.0.
HRMS (ESI) *m*/*z* calculated for $C_{34}H_{40}B_2FeO_4$ $[M+H]^+$ 591.2535, Found:. $[M+H]^+$ 591.2522

2.4 Synthesis of A_2B and A_3

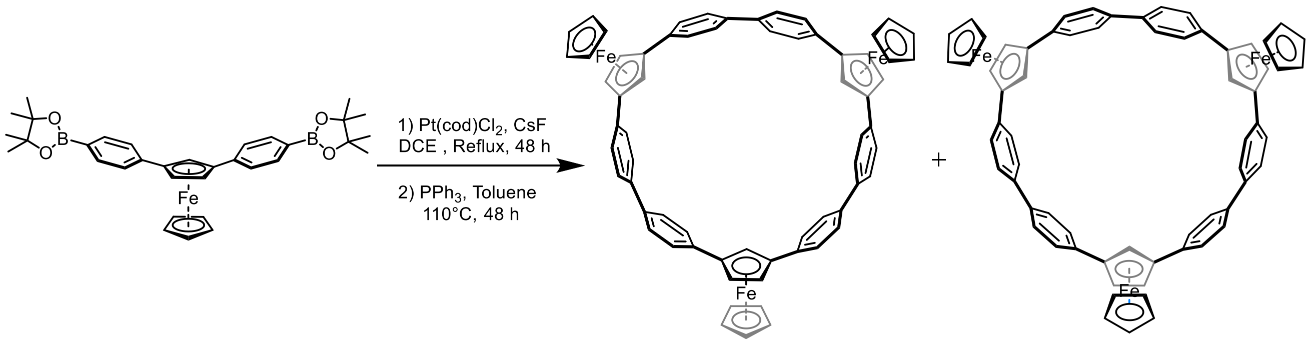

3 A_2B A_3

A mixture of **3** (71 mg, 0.120 mmol, 1 equiv.), $PtCl_2$(cod) (49.5 mg, 0.132 mmol, 1.1 equiv.) and CsF (110 mg, 0.720 mmol, 6 equiv.) in anhydrous 1,2-dichloroethane (DCE) (23 mL) was heated at 85 °C for 48 h under argon. After cooling down to room temperature, the solvent was removed under reduced pressure, methanol (20 mL) was added and the precipitates

were collected by filtration and washed. To the flask containing the crude material was added PPh_3 (315 mg, 1.2 mmol, 10 equiv.) and degassed anhydrous toluene (10 mL) The mixture was stirred at room temperature for 30 min, then heated at 110 °C for 48 h under argon. After cooling down to room temperature, toluene was removed under vacuum. The crude product was purified by silica gel column chromatography (DCM/petroleum ether: 1:2) to afford linear dimer **A_2** (0.1 mg, 0.2%), isomeric mixture of cyclotrimers (10.1 mg, 25%) and isomeric mixture of cyclotetramers (**Fc_4**) (4.84 mg, 11%). A mixture of cyclotrimers was further purified by silica gel column chromatography (ethyl acetate/ petroleum ether: 12:100) several cycles and recrystallization from $CHCl_3$/*n*-pentane to give pure **A_2B** (6.45 mg, 16%) and **A_3** (2.82 mg, 7%) as orange solids. Based on the results of experiments and the analysis of the post-processing, the remaining 64% should be higher linear polymers, and it appears that there are traces of cyclopentamers that could not be separated. In addition, no starting material residues were observed.

A_2
***R_f* =** 0.7 (DCM/petroleum ether: 1:2)
^{1}H NMR (500 MHz, $CDCl_3$) δ 7.64–7.59 (m, 8H), 7.57 (dd, *J* = 8.2, 1.3 Hz, 4H), 7.33 (t, *J* = 7.6 Hz, 4H), 7.23 (t, *J* = 7.4 Hz, 2H), 5.20 (t, *J* = 1.4 Hz, 2H), 4.85 (ddd, *J* = 8.7, 2.5, 1.4 Hz, 4H), 3.96 (s, 10H).
^{13}C{^{1}H} NMR (151 MHz, $CDCl_3$) δ 139.1, 138.6, 138.1, 128.6, 126.8, 126.7, 126.3, 86.6, 86.0, 71.4, 67.6, 67.4, 65.2.
HRMS (ESI) *m*/*z* calculated for $C_{44}H_{34}Fe_2$ $[M+H]^+$ 675.1432, Found:. $[M+H]^+$ 675.1427.

A_2B
***R_f* =** 0.4 (DCM/petroleum ether: 1:2)
***R_f* =** 0.35 (EA/petroleum ether: 12:100)
^{1}H NMR (600 MHz, $CDCl_3$) δ 7.51 (d, *J* = 8.6 Hz, 4H), 7.36 (t, *J* = 7.3 Hz, 12H), 7.31 (d, *J* = 8.0 Hz, 8H), 4.52–4.51 (m, 4H), 4.48 (dd, *J* = 2.4, 1.5 Hz, 2H), 4.35 (s, 10H), 4.32 (s, 5H), 3.62 (t, *J* = 1.5 Hz, 2H), 3.60 (t, *J* = 1.5 Hz, 1H).
^{13}C{^{1}H} NMR (151 MHz, $CDCl_3$) δ 139.9, 139.8, 138.0, 137.7, 137.7, 128.9, 128.6, 128.5, 127.3, 125.9, 89.8, 89.4, 89.0, 81.2, 80.9, 70.1, 70.1, 64.1, 63.7, 63.6.
FTICR (MALDI) *m*/*z* calculated for $C_{66}H_{48}Fe_3$ $[M]^+$ 1008.1804, Found: 1008.1808.

A_3
***R_f* =** 0.4 (DCM/petroleum ether: 1:2)
***R_f* =** 0.32 (ethyl acetate/petroleum ether: 12:100)
^{1}H NMR (600 MHz, $CDCl_3$) δ 7.47 (d, *J* = 8.5 Hz, 12H), 7.35 (d, *J* = 8.5 Hz, 12H), 4.50 (d, *J* = 1.5 Hz, 6566H), 4.32 (s, 15H), 3.66 (t, *J* = 1.6 Hz, 3H).
^{13}C{^{1}H} NMR (151 MHz, $CDCl_3$) δ 138.2, 137.6, 128.7, 126.1, 89.3, 80.0, 70.2, 64.1.
FTICR (MALDI) *m*/*z* calculated for $C_{66}H_{48}Fe_3$ $[M]^+$ 1008.1804, Found: 1008.1796.

Fc_4
***R_f* =** 0.2 (DCM/petroleum ether: 1:2)
^{1}H NMR (500 MHz, $CDCl_3$) δ 7.54–7.46 (m, 23H), 7.43–7.39 (m, 9H), 4.63 (ddd, *J* = 4.7, 3.1, 1.5 Hz, 8H), 4.33 (t, *J* = 1.5 Hz, 1H), 4.29 (t, *J* = 1.5 Hz, 2H), 4.26–4.21 (m, 21H).
FTICR (MALDI) *m*/*z* calculated for $C_{88}H_{64}Fe_4$ $[M]^+$ 1344.2406, Found: 1344.2408.

3. X-Ray Crystallography

Table S1. Crystallographic data and structure refinement details for **2**, **A_2B** and **A_3**

Compound	**2**	**A_2B**	**A_3**
CCDC Deposition number	2244808	2243015	2244807
Empirical formula	$C_{22}H_{16}Cl_2Fe$	$C_{66}H_{48}Fe_3$	$C_{66}H_{48}Fe_3$
Formula weight	407.09	1008.59	1008.59
Temperature/K	296.15	104(6)	104(6)
Crystal system	monoclinic	orthorhombic	monoclinic
Space group	P1	$P2_12_12_1$	$P2_1/c$
a/Å	10.9681(16)	11.8673(6)	14.3925(5)
b/Å	7.6771(11)	13.7758(7)	11.6493(4)
c/Å	21.343(4)	31.2560(19)	34.8215(11)
α/°	90	90	90
β/°	90.092(6)	90	99.292(3)
γ/°	90	90	90
Volume/$Å^3$	1797.2(5)	5109.8(5)	5761.7(3)
Z	1	4	4
ρ_{calc} g/cm^3	1.505	1.311	1.163
μ/mm	1.137	4.865	4.315
F(000)	832.0	2088.0	2088.0
Crystal size/mm^3	0.12 × 0.11 × 0.08	0.15 × 0.11 × 0.06	0.2 × 0.11 × 0.05
Radiation	Mo Kα (λ = 0.71073)	Ga Kα (λ = 1.3405)	Ga Kα (λ = 1.3405)
2Θ range for data collection/°	4.172 to 49.994	4.916 to 112.67	4.472 to 120.958
Index ranges	-13 ≤ h ≤ 13, -9 ≤ k ≤ 9, -25 ≤ l ≤ 25	-10 ≤ h ≤ 14, -16 ≤ k ≤ 16, -38 ≤ l ≤ 34	-18 ≤ h ≤ 15, -14 ≤ k ≤ 15, -44 ≤ l ≤ 44
Reflections collected	21893	19153	79602
Independent reflections	11193 [R_{int} = 0.0608, R_{sigma} = 0.0911]	9301 [R_{int} = 0.0610, R_{sigma} = 0.0837]	12902 [R_{int} = 0.0457, R_{sigma} = 0.0296]
Data/restraints/parameters	11193/3/901	9301/0/622	12902/0/622
Final R indexes [I>=2σ (I)]	0.974	1.050	1.187
Final R indexes [all data]	R_1 = 0.0409, wR_2 = 0.0924	R_1 = 0.0682, wR_2 = 0.1691	R_1 = 0.0797, wR_2 = 0.1605
Largest diff. peak/hole / e $Å^{-3}$	R_1 = 0.0567, wR_2 = 0.1010	R_1 = 0.0912, wR_2 = 0.1807	R_1 = 0.0846, wR_2 = 0.1628
Final R indexes [I>=2σ (I)]	0.27/-0.25	0.71/-0.51	1.25/-0.88

These data can be obtained free of charge from The Cambridge Crystallographic Data Centre via www.ccdc.cam.ac.uk/data_request/cif.

3.1 Single-crystal Structure and Packing of 2

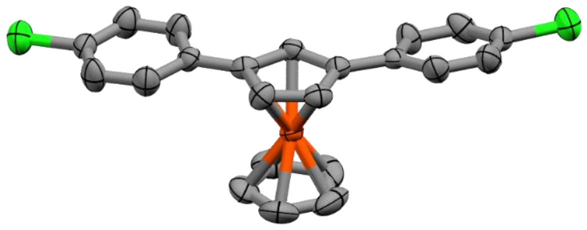

Figure S1. X-ray crystallographic structures of **2**. (thermal ellipsoids shown at 50% probability, All hydrogen atoms was omitted for clarity)

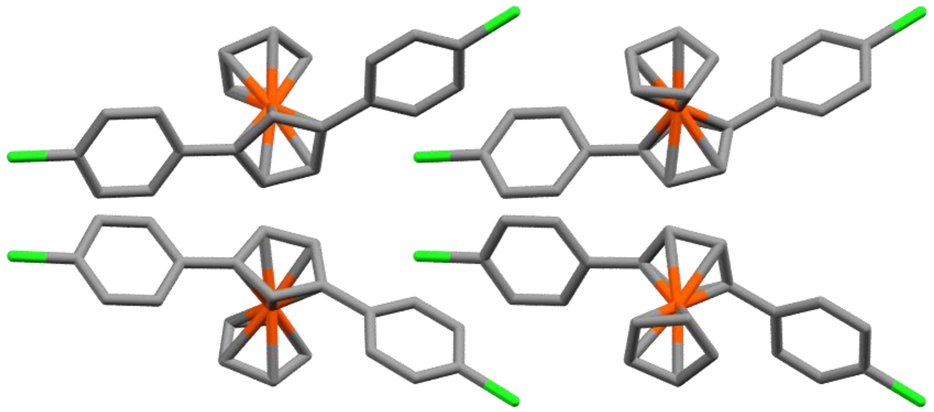

Figure S2. Packing of **2**. (All hydrogen atoms was omitted for clarity)

3.2 Single-crystal Structure and Packing of A_2B

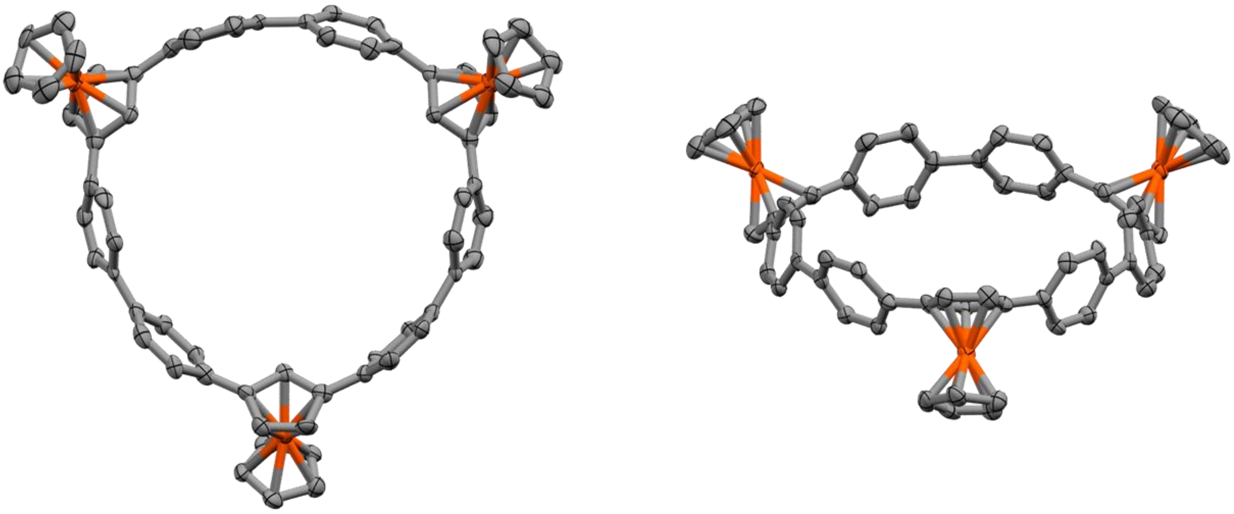

Figure S3. X-ray crystallographic structures of $\mathbf{A_2B}$ (thermal ellipsoids shown at 50% probability. All hydrogen atoms and solvent was omitted for clarity)

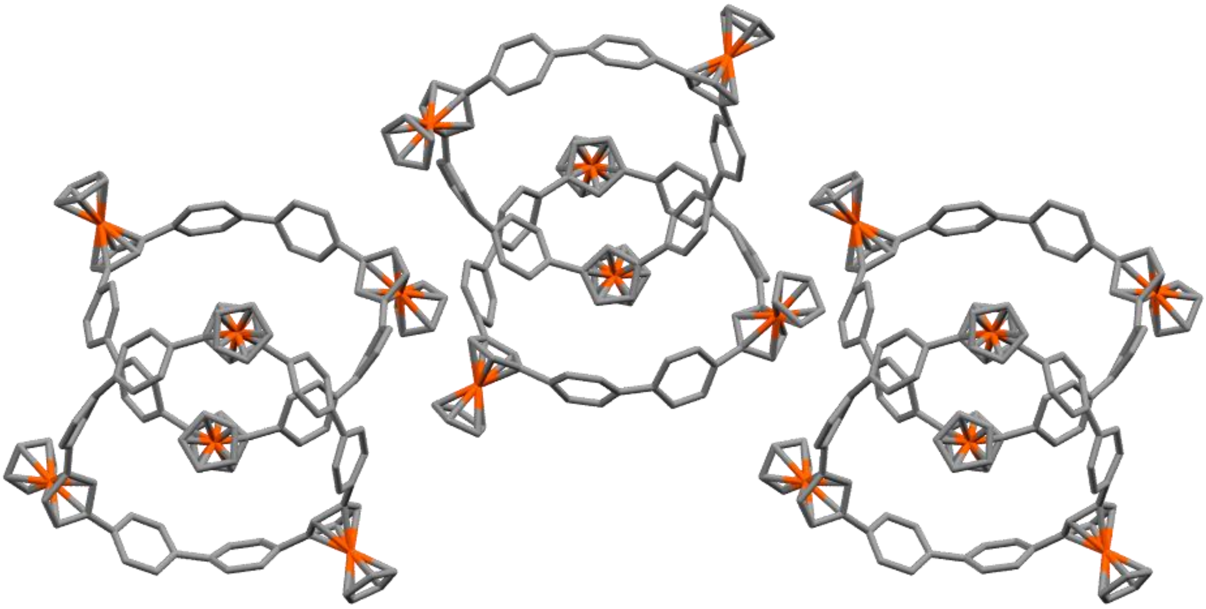

Figure S4. Packing of $\mathbf{A_2B}$ (All hydrogen atoms and solvent was omitted for clarity)

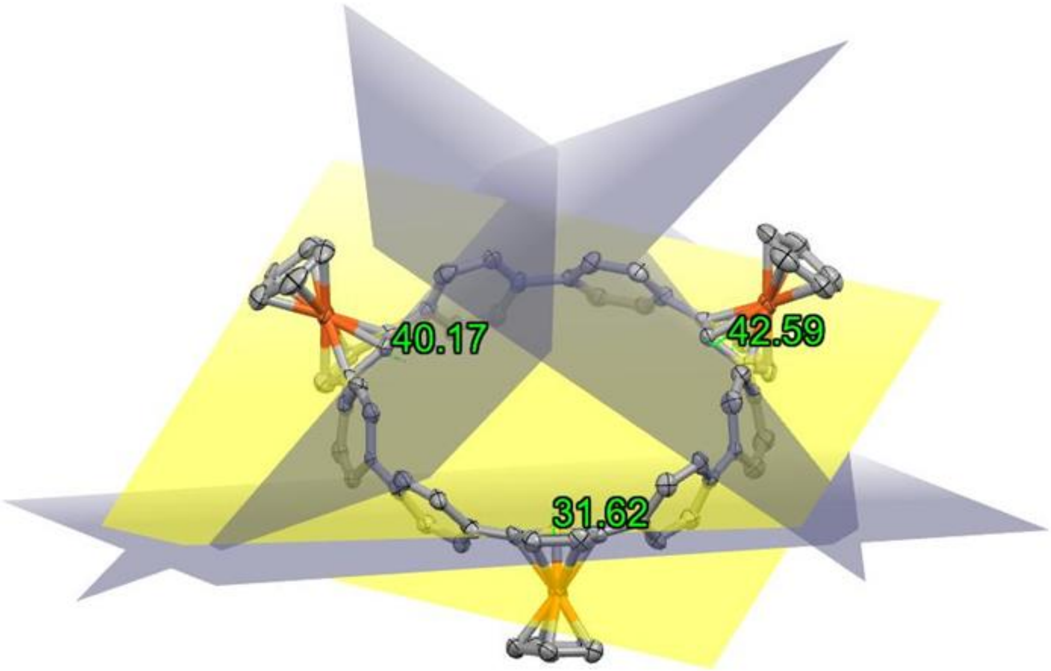

Figure S5. Measurement of ferrocenyl rotational angles in **A$_2$B**

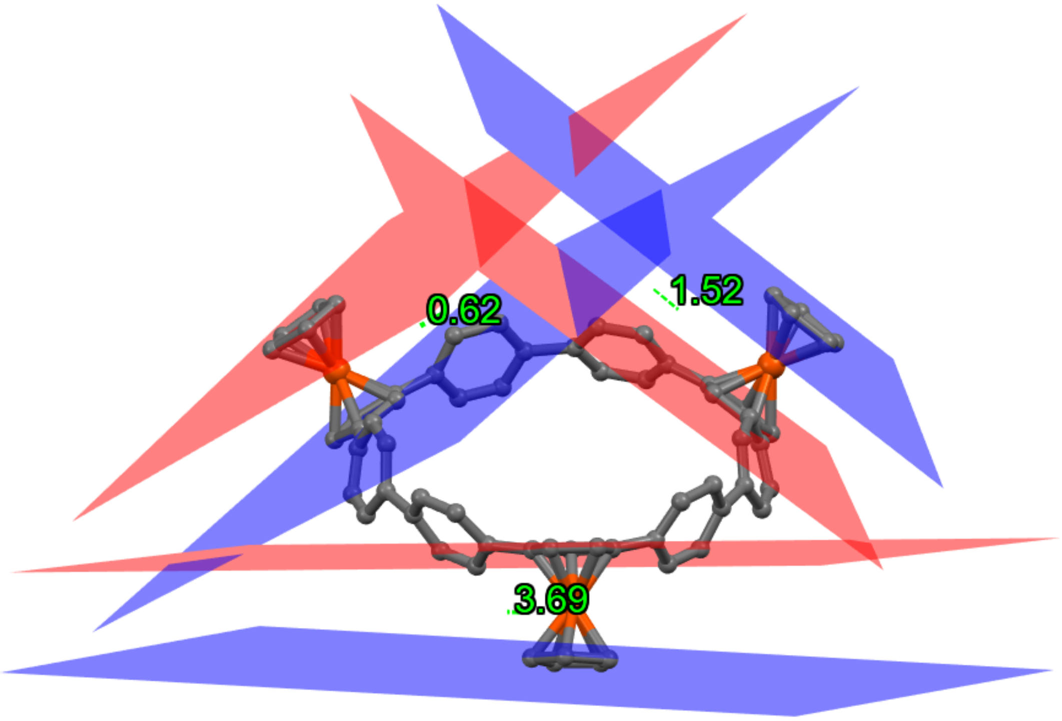

Figure S6. Measurement of the tilting of FeCp in **A$_2$B.**

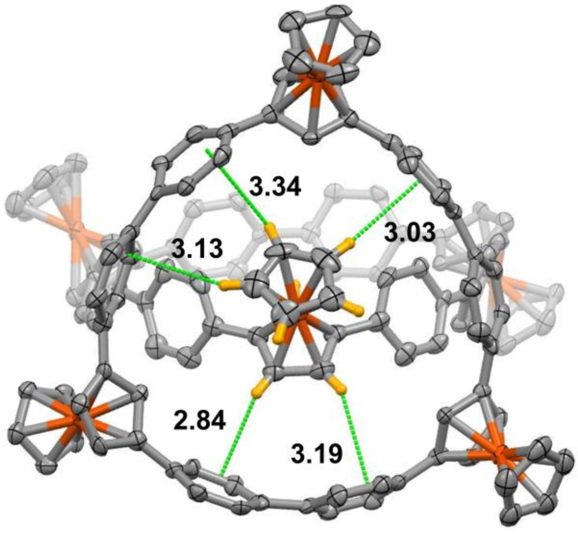

d_{PLN}(Å)	∠C-H-X (°)	d_{C-X} (Å)
3.13	172.92	4.26
3.34	119.19	3.93
3.03	153.75	3.91
3.19	131.95	3.97
2.84	158.15	4.10

Figure S7. Definition of C-H-π interactions in crystal stacking of **A_2B.**

3.3 Single-crystal Structure and Packing of A_3

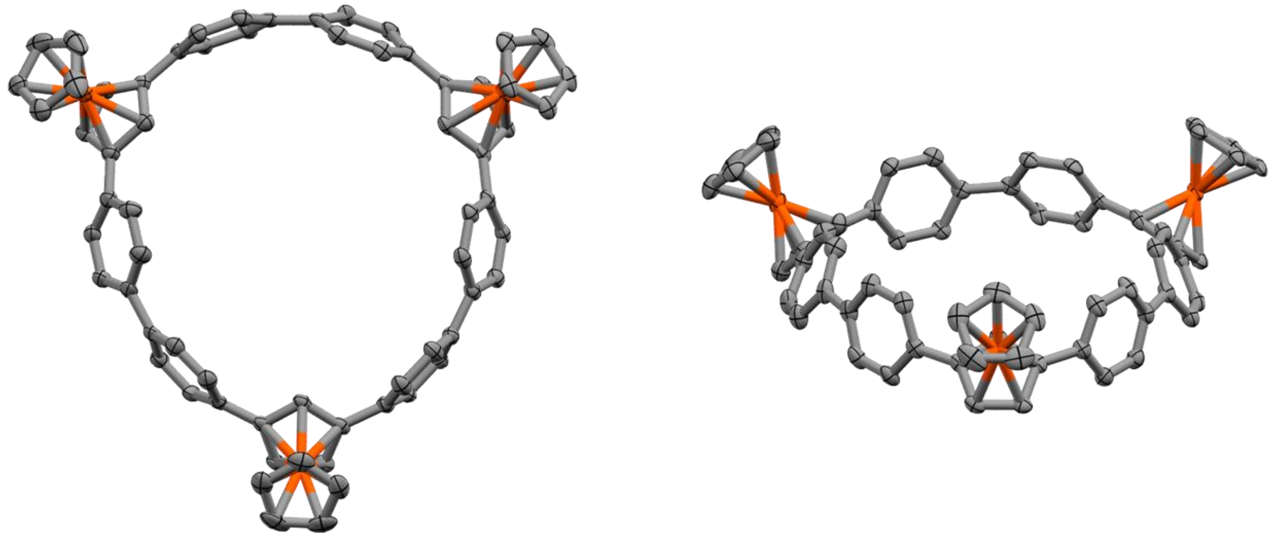

Figure S8. X-ray crystallographic structures of **A_3** (thermal ellipsoids shown at 50% probability. All hydrogen atoms and solvent was omitted for clarity)

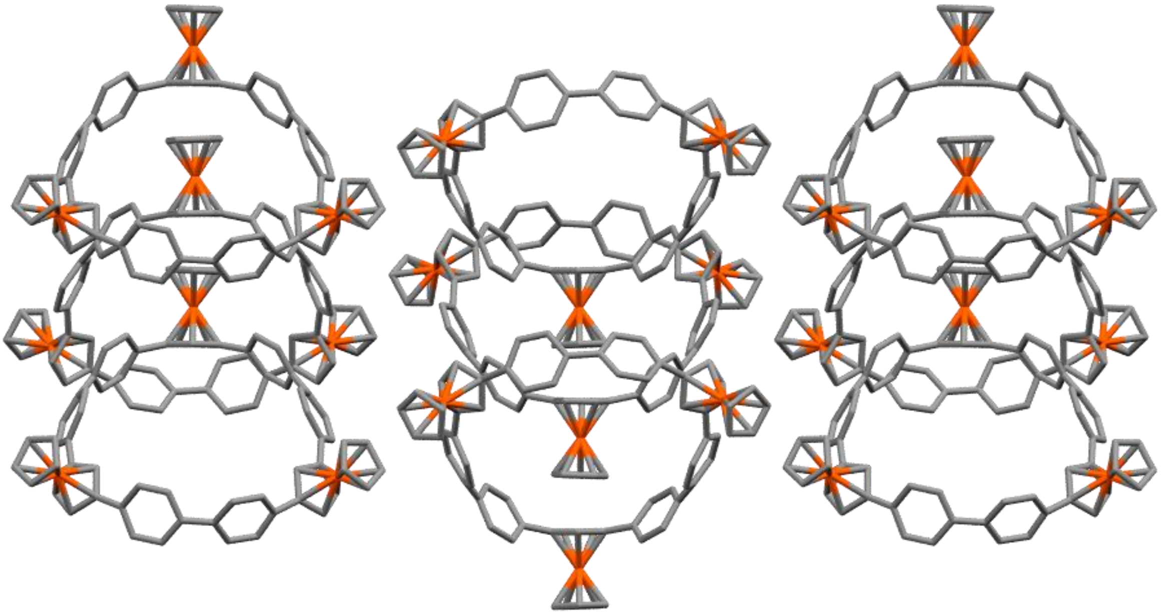

Figure S9. Packing of **A**$_3$ (All hydrogen atoms and solvent was omitted for clarity)

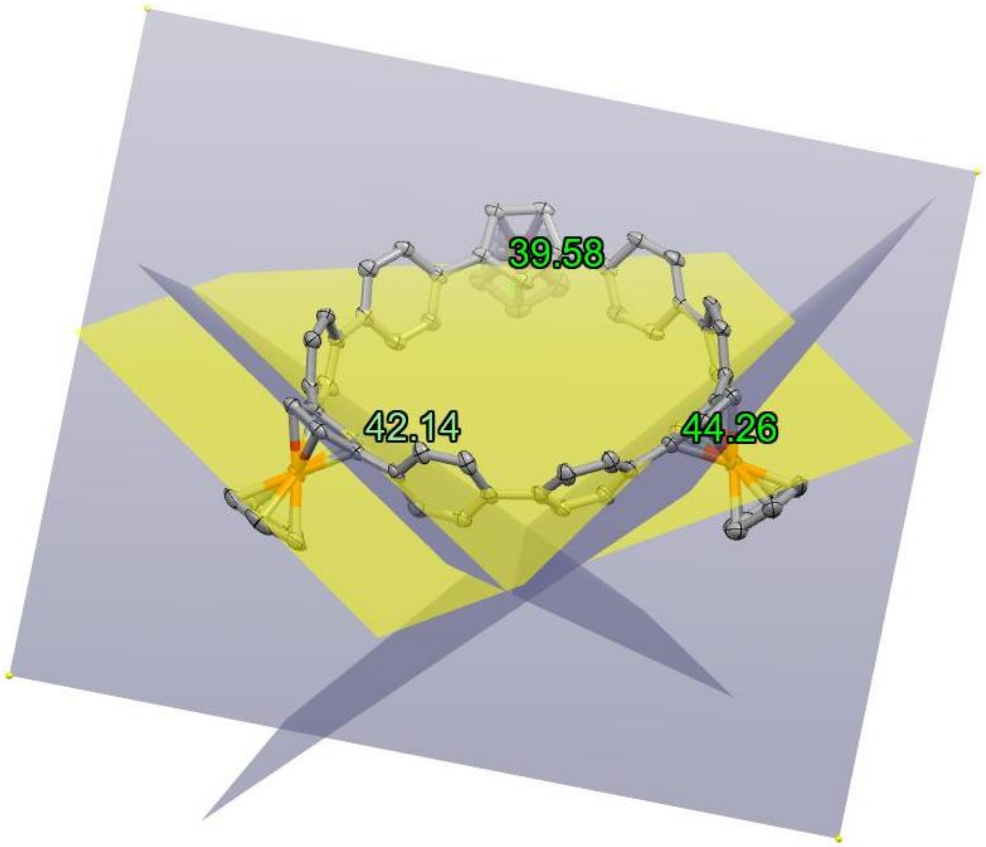

Figure S10. Measurement of ferrocenyl rotational angles in **A**$_3$.

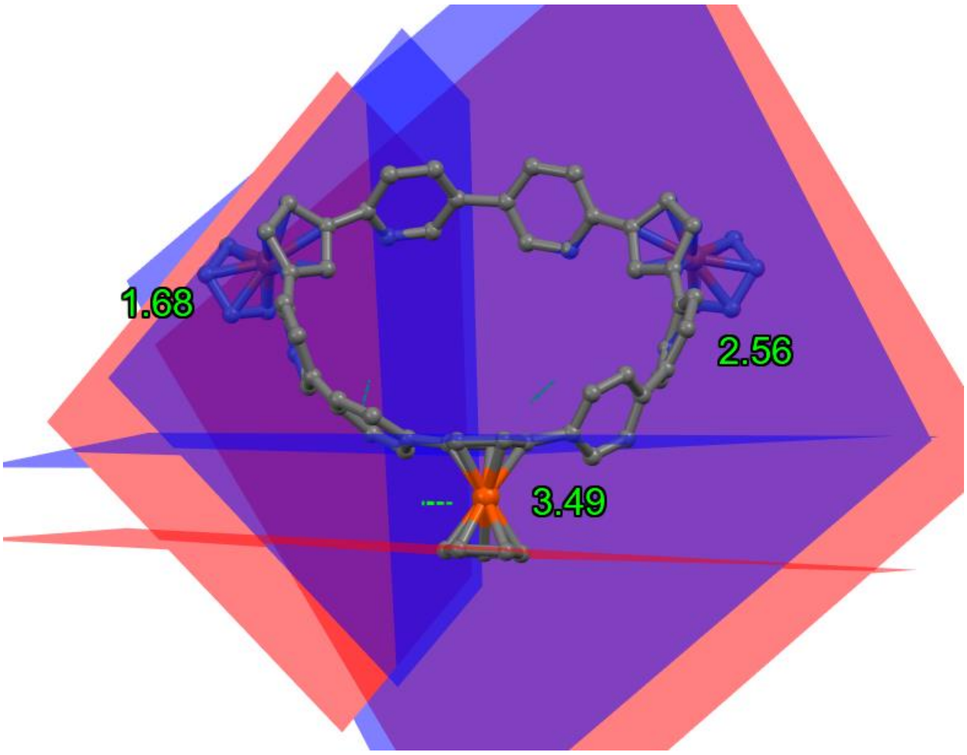

Figure S11. Measurement of the tilting of FeCp in **A**$_3$.

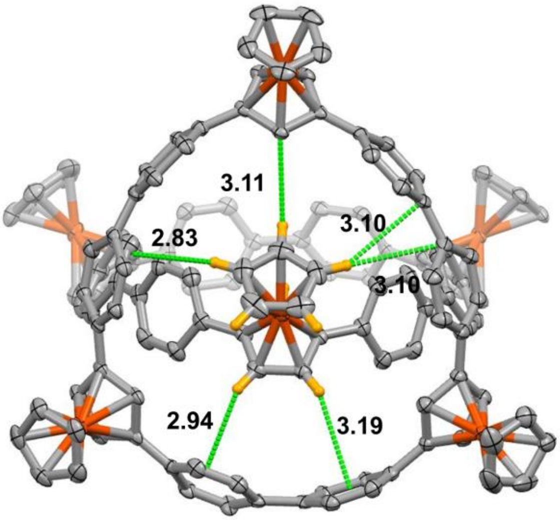

d_{PLN}(Å)	∠C-H-X (°)	d_{C-X} (Å)
2.93	154.10	4.35
3.11	123.24	3.71
3.10	152.61	3.96
3.19	138.94	3.95
2.94	140.95	3.88

Figure S12. Definition of C-H-π interactions in crystal stacking of **A**$_3$.

4. Electrochemistry

Electrochemical measurements were carried out with Metrohm Autolab PGSTAT204 electricity workstation at room temperature. Cyclic voltammograms (CV) and differential pulse voltammogram (DPV) were carried out using a conventional three-electrode system equipped with a glassy carbon working electrode (1.5 mm diameter), a platinum counter electrode, and Ag/AgCl (3 mol L^{-1} KCl) reference electrode. The commercial electrolytes Na[BArF] and [*n*-Bu_4N][PF_6] were purified by recrystallisation. [*n*-Bu_4N][BArF] was synthesized as previously reported.[4] Analyte solutions were between 0.1-1 mM.

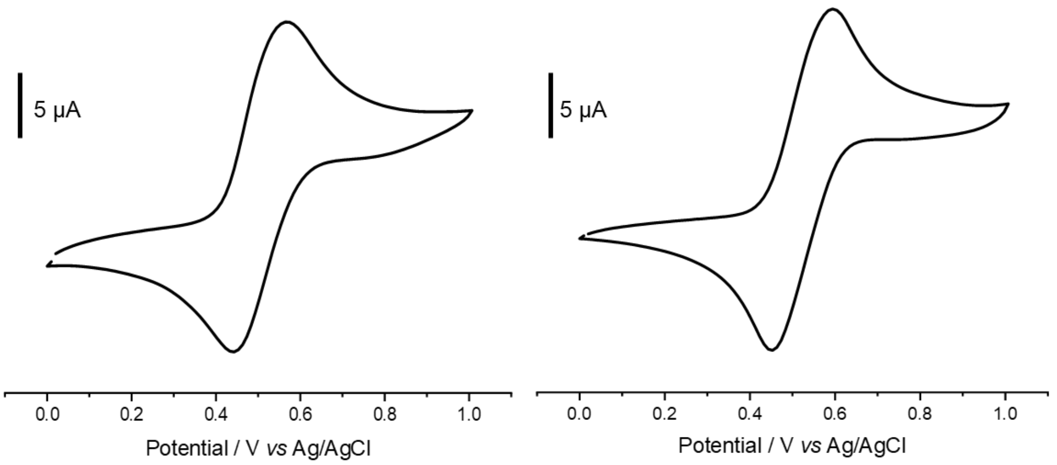

Figure S13. Cyclic voltammograms of **A_2B** (left) and **A_3** (right) recorded in CH_2Cl_2/0.1 M *n*-Bu_4NPF_6 at scan rate of 100 mV/s.

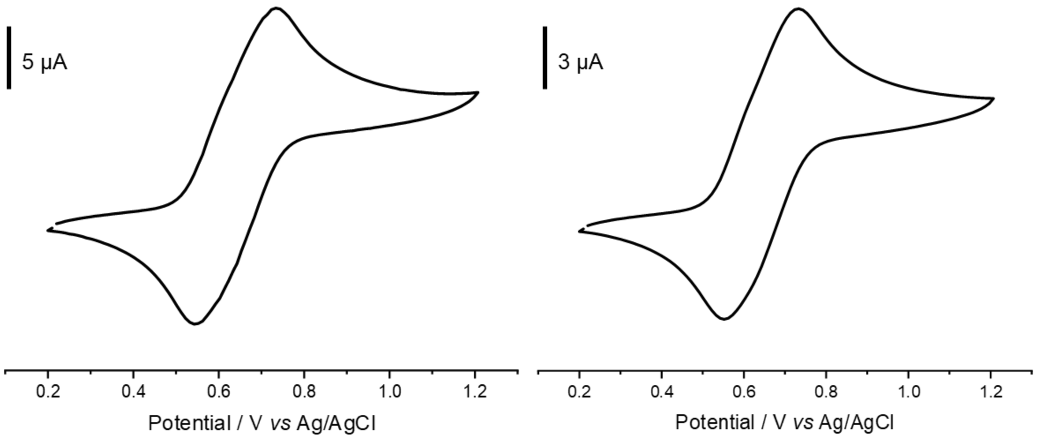

Figure S14. Cyclic voltammograms of **A_2B** (left) and **A_3** (right) recorded in CH_2Cl_2/0.1 M *n*-Bu_4NBArF at scan rate of 100 mV/s.

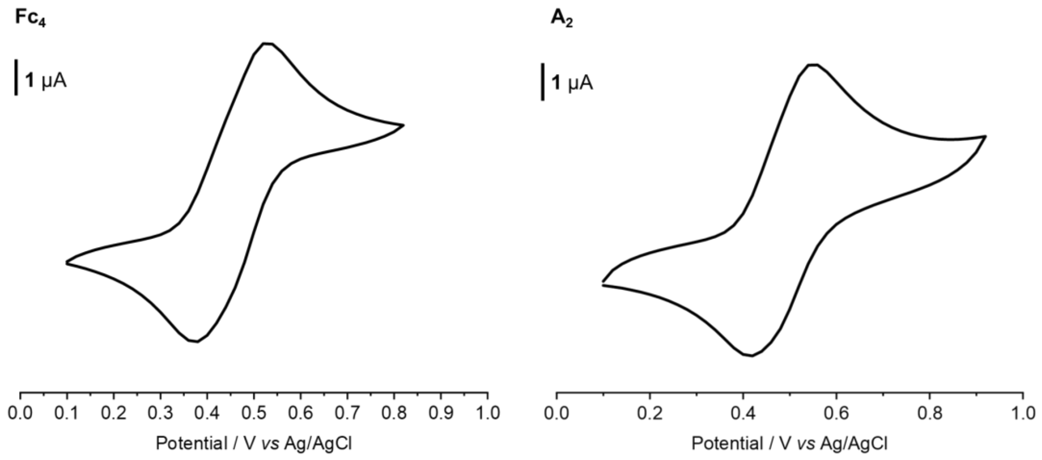

Figure S15. Cyclic voltammograms of **Fc_4** (left) and **A_2** (right) recorded in CH_2Cl_2/0.1 M *n*-Bu_4NBArF at a scan rate of 100 mV/s.

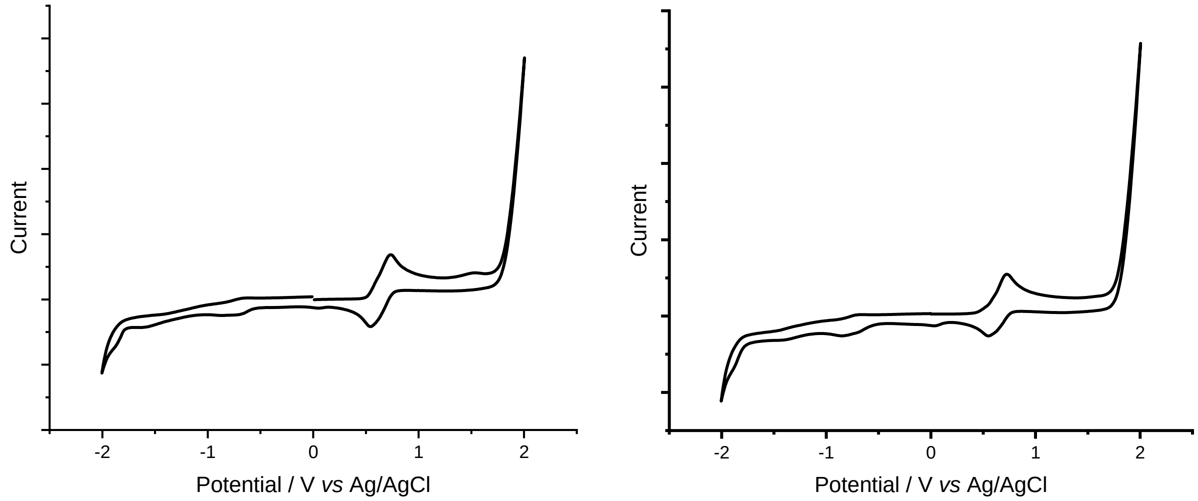

Figure S16. Cyclic voltammograms of **A_2B** (left) and **A_3** (right) with a large potential sweep recorded in CH_2Cl_2/0.1 M *n*-Bu_4NBArF at a scan rate of 100 mV/s.

5. NLO measurements

The NLO properties of the $\mathbf{A_2B}$ crystal were examined with a home-built femtosecond pumped scanning microscope[5] (800~1040 nm, Spectra Physics Mai Tai, 100 fs, 82 MHz; 1200~1500 nm, Spectra Physics Ace, TOPAS Prime, ~50 fs, 1 KHz) in reflection geometry with the incidence and detection angles at 45°.

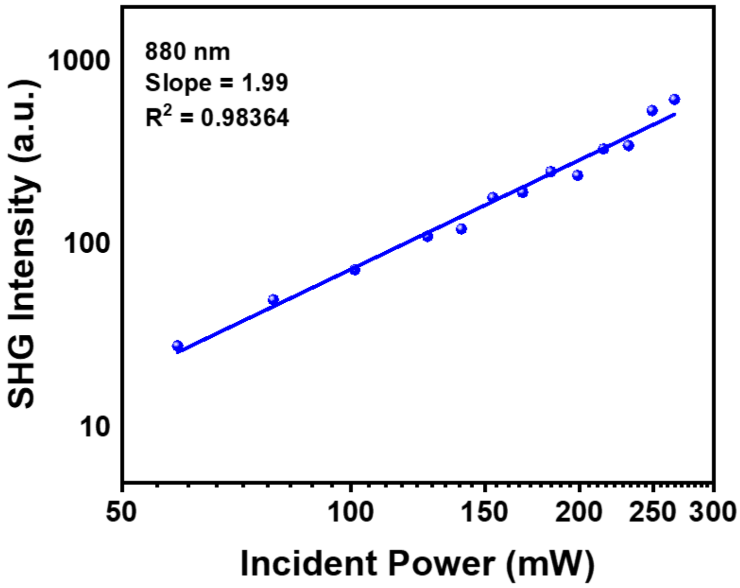

Figure S17. Logarithmic plot of the SHG intensity as a function of incident power. The solid line is a linear fit with a slope of 1.99.

6. DFT Calculations

The density functional theory (DFT) calculations were performed using Gaussian 16 (Revision C.01) program[6]. DFT geometry optimizations were carried out in unconstrained C1 symmetry. If not stated otherwise, all calculations were carried out employing the B3LYP hybrid functional and basis sets 6-31G(d) for C, H, and SDD[7] (Stuttgart relativistic small-core ECP basis) for Fe. In addition, the optical transition diagrams were obtained through TD-DFT calculated at the level of PBE0-D3(BJ)/Def2-SVP. Each structure was optimized to meet standard convergence criteria and the existence of a local minimum was verified by a normal mode frequency calculation (with no imaginary frequency).

6.1 Calculated parameters

**PBE0-D3(BJ)/Def2-SVP**	***E [hartree]***	***E+ZPE [hartree]***	***H [hartree]***	***G [hartree]***	**HOMO [eV]**	**LUMO [eV]**
A_2B	-6329.4153	-6328.4739	-6328.4200	-6328.5644	-5.63	-1.22
A_3	-6329.4117	-6328.4703	-6328.4163	-6328.5610	-5.63	-1.26
**B3LYP/SDD, 6-31g(d)**	***E* [hartree]**	***E+ZPE* [hartree]**	***H* [hartree]**	***G* [hartree]**	**HOMO [eV]**	**LUMO [eV]**
Monomer	-973.1076	-972.7761	-972.7568	-972.8246	-5.27	-0.56
Dimer	-1945.0246	-1944.3816	-1944.3433	-1944.4571	-5.17	-1.07
A_2B	-2915.6796	-2914.7466	-2914.6915	-2914.8384	-5.10	-1.08
A_3	-2915.6760	-2914.7430	-2914.6878	-2914.8347	-5.10	-1.12
AAAA	-3887.6220	-3886.3772	-3886.3030	-3886.4947	-5.11	-1.09
AAAB	-3887.6170	-3886.3722	-3886.2981	-3886.4889	-5.18	-1.03
AABB	-3887.6231	-3886.3782	-3886.3040	-3886.4951	-5.11	-1.09
ABAB	-3887.6086	-3886.3645	-3886.2899	-3886.4836	-5.13	-1.09

Table S2. Electronic energy (***E***), sum of electronic energies including zero-point-energy (***ZPE***), sum of electronic and thermal enthalpies (***H***), sum of electronic and free energies (***G***).

6.2 Calculated molecular geometries

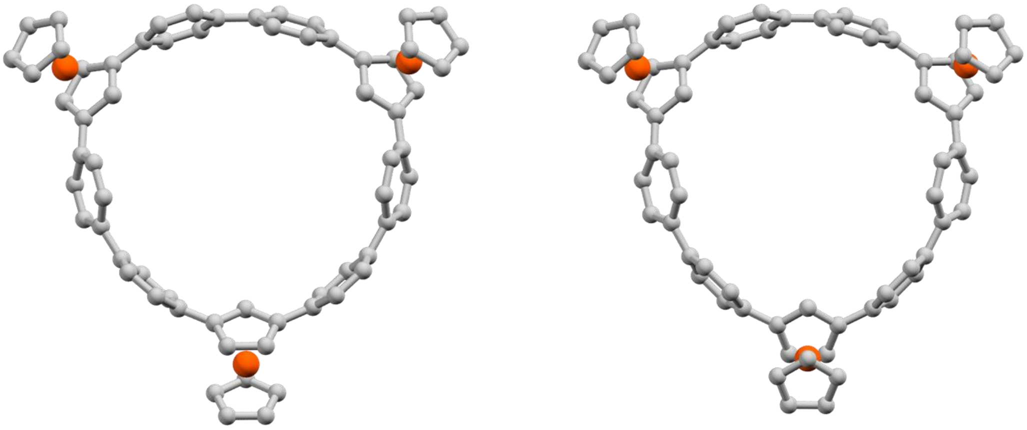

A_2B A_3

Figure S18. Calculated trimeric macrocycles geometries (PBE0-D3(BJ)/Def2-SVP)

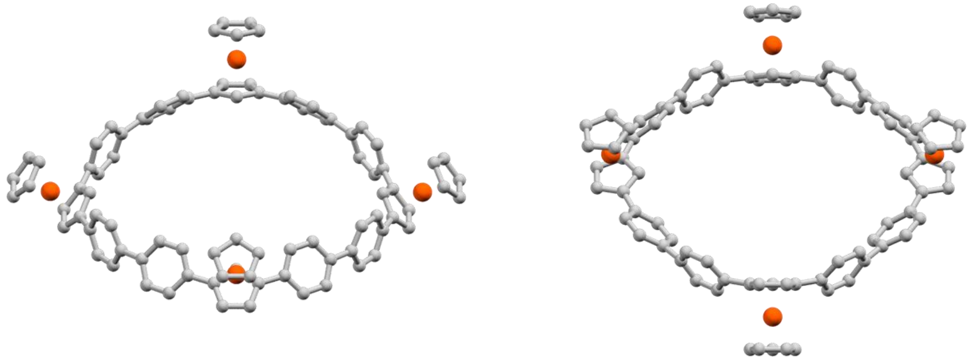

AAAA AAAB

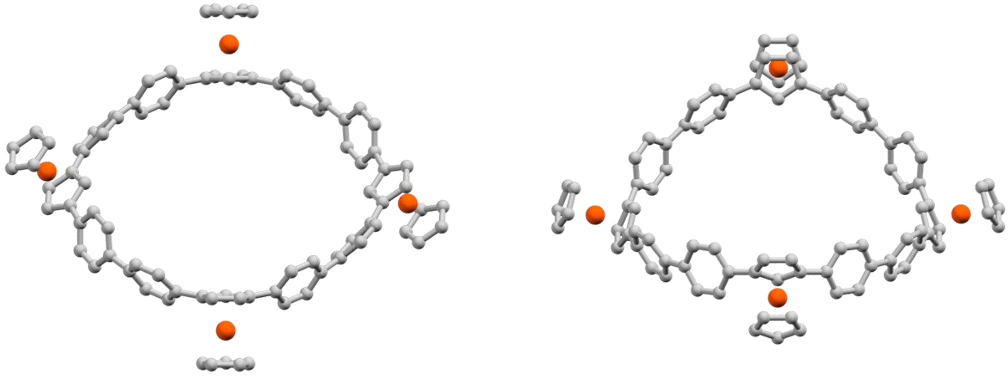

AABB ABAB

Figure S19. Calculated tetrameric macrocycles geometries (B3LYP/SDD, 6-31G(d))

6.3 Rotational Barriers

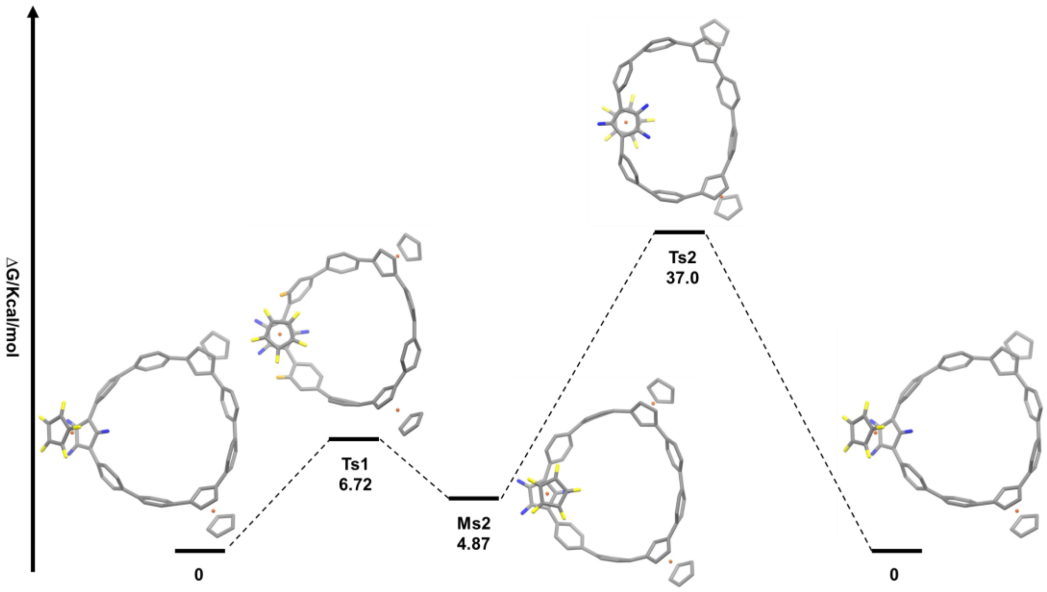

Figure S20. The flipping barrier of *anti* ferrocenyl in **A_2B** (B3LYP/SDD, 6-31G(d))

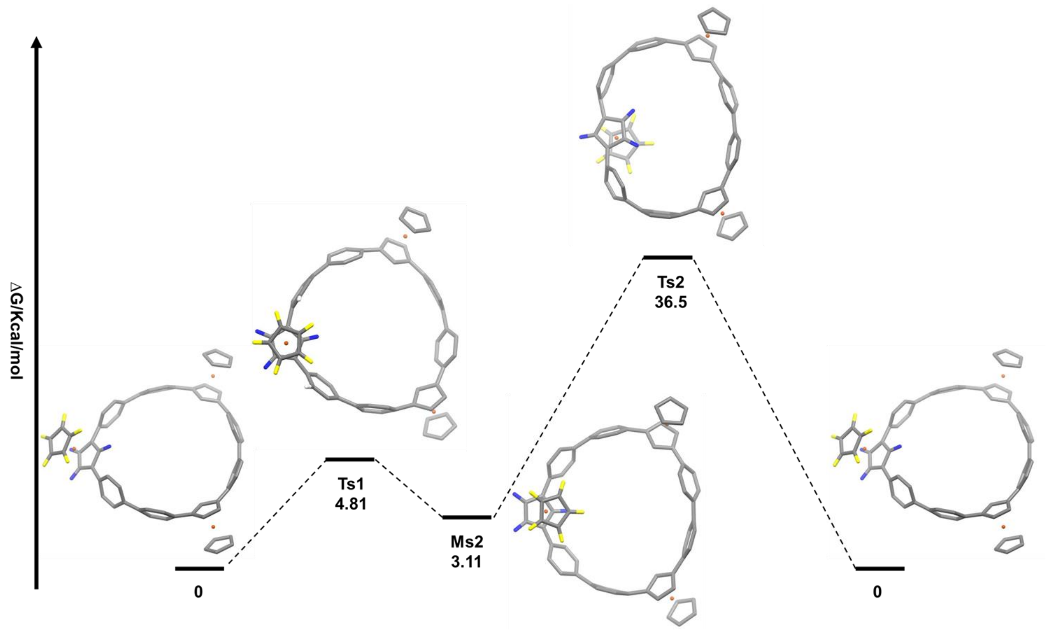

Figure S21. The flipping barrier of *syn* ferrocenyl in **A_2B** (B3LYP/SDD, 6-31G(d))

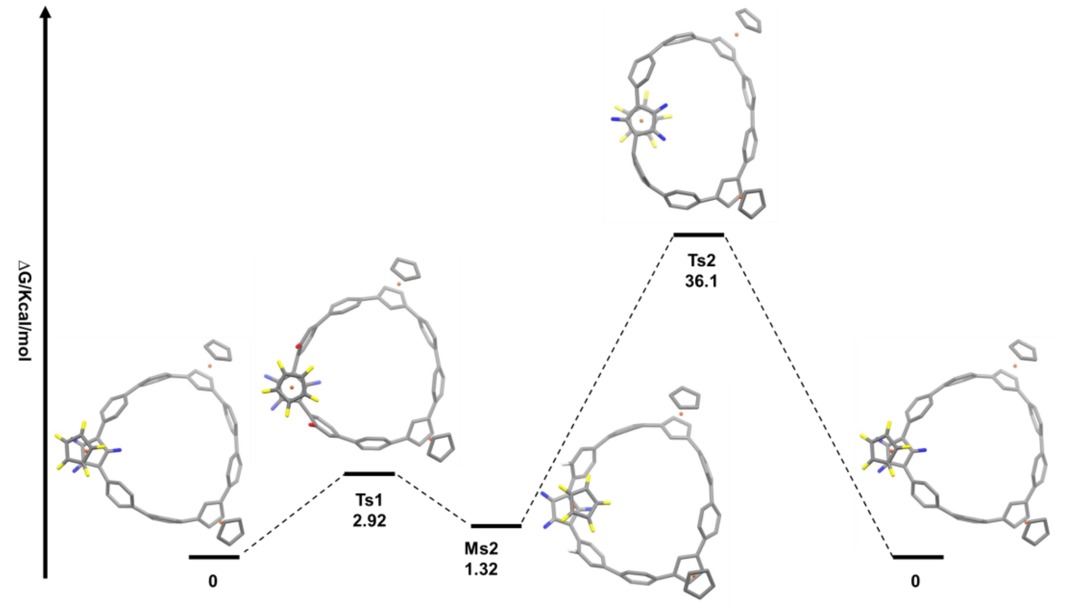

Figure S22. The flipping barrier of ferrocenyl in **A**$_3$ (B3LYP/SDD, 6-31G(d))

6.4 TD-DFT calculation

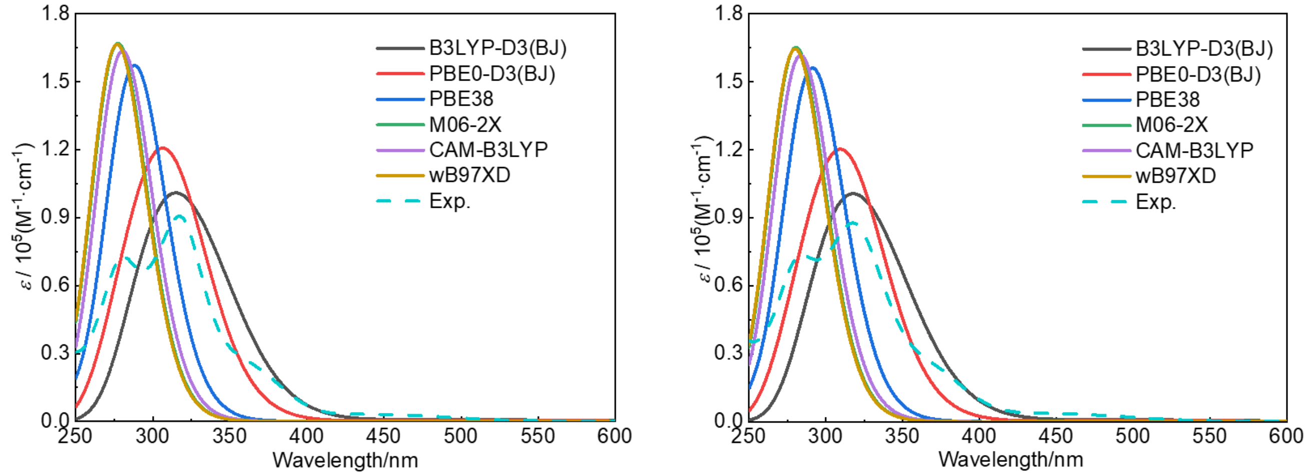

Figure S23. TD-DFT calculated optical transition diagrams (Screening different functions for **A_2B (left)** and **A_3 (right)** at the Def2-SVP basis set)

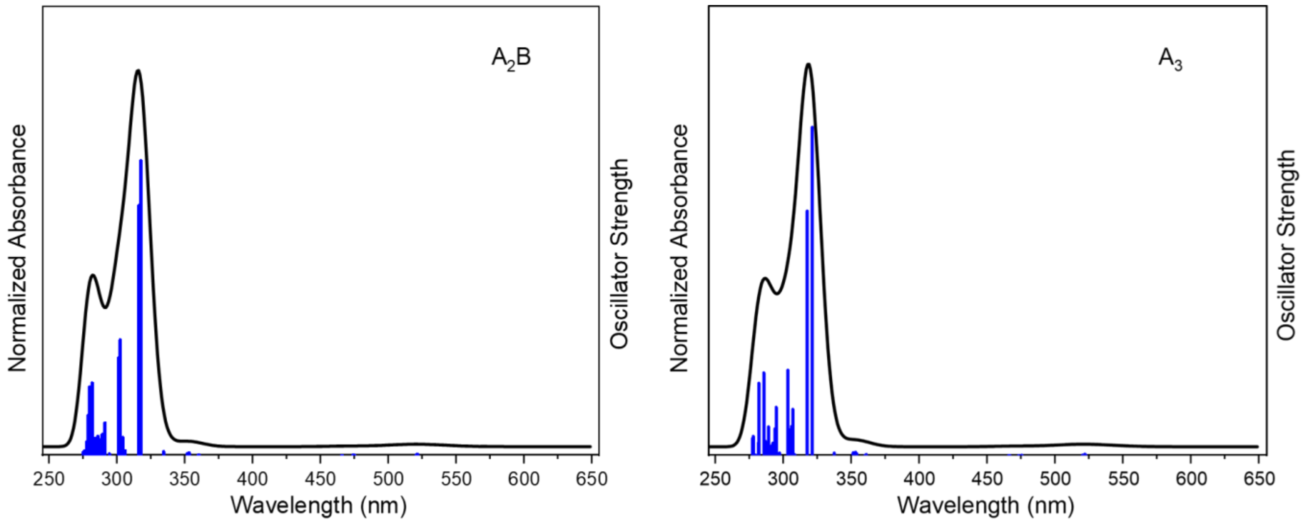

Figure S24. TD-DFT Absorbance of **A_2B** and **A_3** (PBE0-D3(BJ)/Def2-SVP)

Table S3. Major electronic transitions for **A_2B** (PBE0-D3(BJ)/Def2-SVP)

Energy (eV)	Wavelength (nm)	Osc. Strength	Major contributions
2.6891	461.06	0.0003	H-9 -> L+12 27.1%, H-3 -> L+12 11.2%
3.6880	336.18	0.0158	H -> L 67.1%, H -> L+1 8.0%
3.8910	318.64	1.0610	H -> L+1 44.0%, H-1 -> L 23.2%, H-1 -> L+1 7.0%
3.9097	317.12	0.9021	H -> L+2 37.5%, H-2 -> L 31.9%, H-1 -> L 11.4%
4.0520	305.98	0.0142	H-2 -> L 20.3%, H-3 -> L 18.5%, H-4 -> L 13.9%, H-5 -> L 10.0%
4.0679	304.79	0.1358	H-1 -> L 28.0%, H -> L+2 18.1%
4.1053	302.01	0.2739	H-3 -> L 25.6%, H-1 -> L+1 17.1%, H-3 -> L+1 10.5%
4.3323	286.19	0.0493	H-4 -> L+2 18.8%, H-3 -> L+1 10.4%
4.3954	282.08	0.1900	H-6 -> L+1 21.7%, H-7 -> L 18.5%
4.4202	280.49	0.1865	H-8 -> L 28.2%, H-6 -> L+2 11.6%
4.4724	277.22	0.0056	H -> L+4 22.9%, H-7 -> L+2 12.0%, H-4 -> L+2 8.4%

Table S4. Major electronic transitions for **A_3** (PBE0-D3(BJ)/Def2-SVP)

Energy (eV)	Wavelength (nm)	Osc. Strength	Major contributions
2.6645	465.32	0.0003	H-11 -> L+11 27.7%, H-11 -> L+12 11.8%
3.6768	337.21	0.0084	H -> L 70.5%, H-1 -> L+1 9.2%
3.8629	320.96	1.0957	H -> L+1 37.6%, H-1 -> L 34.8%
3.9065	317.38	0.8157	H -> L+2 41.1%, H-2 -> L 39.5%
4.0247	308.06	0.0034	H-3 -> L 19.5%, H-2 -> L 12.2%, H-3 -> L+1 11.5%
4.0647	305.03	0.0874	H-5 -> L 53.1%, H-4 -> L 17.7%, H-1 -> L+2 5.3%
4.0900	303.14	0.2851	H-3 -> L 25.1%, H -> L+2 16.3%, H-2 -> L 15.0%
4.2090	294.57	0.1615	H-1 -> L+2 30.2%, H-1 -> L 14.8%
4.2658	290.65	0.0312	H-6 -> L 20.0%, H-2 -> L+2 9.2%, H-4 -> L+1 9.2%
4.3446	285.38	0.2758	H-7 -> L 28.9%, H-6 -> L+1 15.1%, H-3 -> L+2 9.6%
4.4005	281.75	0.2417	H-7 -> L+2 28.6%, H-6 -> L+2 10.8%, H -> L+3 9.5%
4.4654	277.66	0.0641	H-3 -> L+1 13.6%, H-4 -> L+2 10.2%

6.5 Strain energy calculations

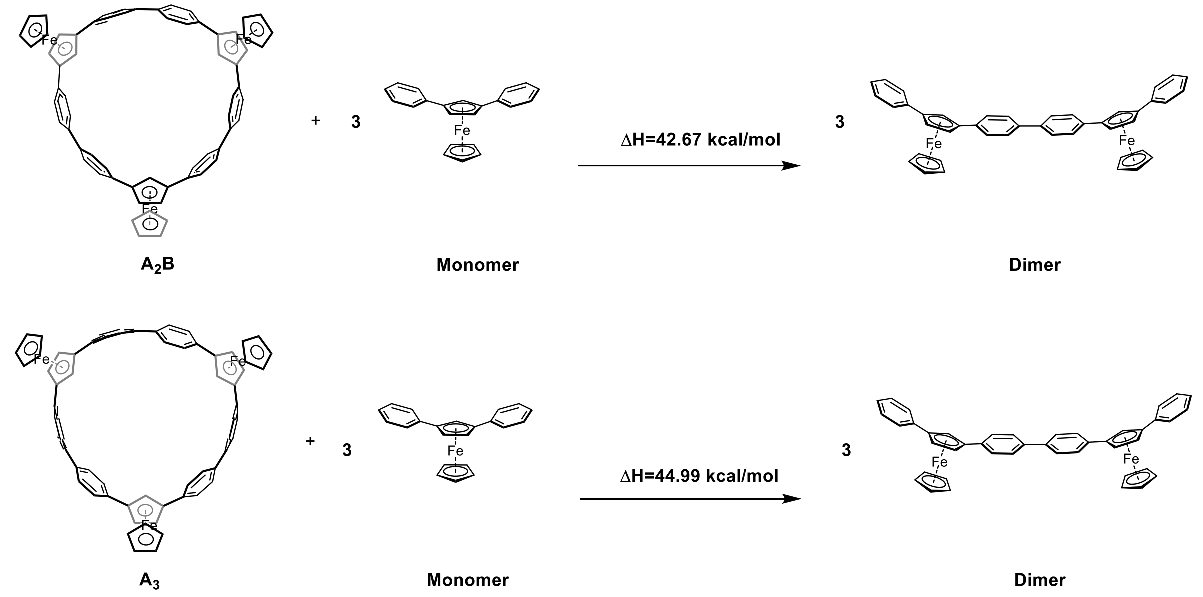

Scheme S1. Homodesmotic reactions used for estimation of strain energies. (B3LYP/SDD, 6-31G(d))

Table S5. Homodesmotic reactions for the calculation of strain energies. (B3LYP/SDD, 6-31G(d))

	H **[hartree]**	***ΔH*** **[kcal/mol]**
**Monomer**	-972.7568	--
**Dimer**	-1944.3433	--
**A_2B**	-2914.6915	42.67
**A_3**	-2914.6878	44.99
**AAAA**	-3886.3030	26.98
**AAAB**	-3886.2981	30.06
**AABB**	-3886.3040	26.35
**ABAB**	-3886.2899	35.20

StrainViz program analysis

Table S6. The strain energies calculated by StrainViz and classified in total strain, bond strain, angle strain and torsion strain (B3LYP/SDD, 6-31G(d)).

	**Total strain [kcal/mol]**	**Bond strain [kcal/mol]**	**Angle strain [kcal/mol]**	**Dihedral strain [kcal/mol]**
**A_2B**	42.24	1.04	3.09	38.1
**A_3**	44.47	1.10	3.51	39.8

6.6 Frontier Molecular Orbitals

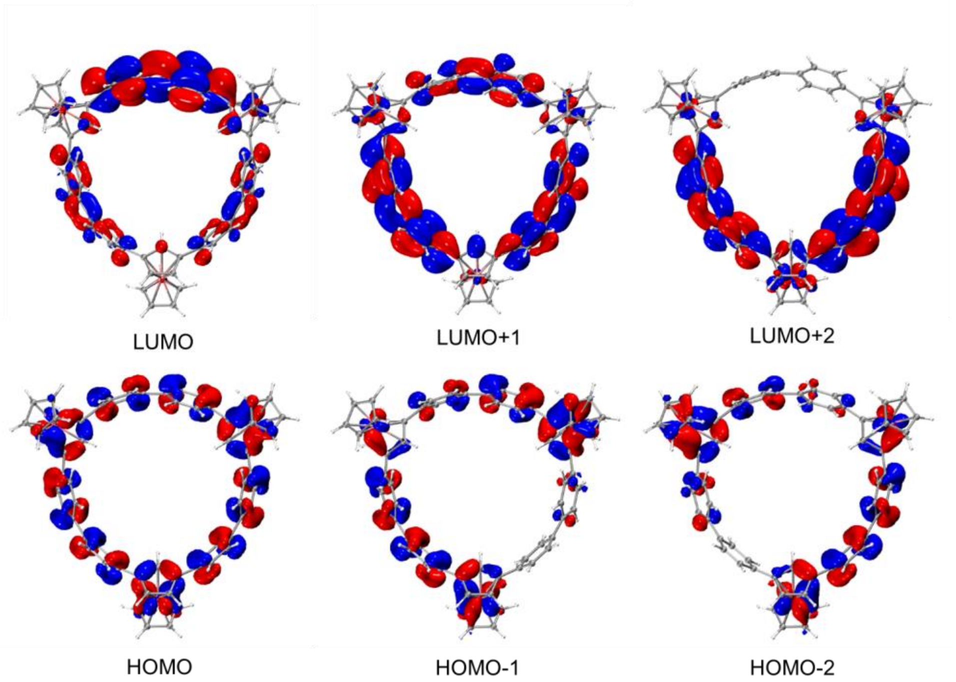

Figure S25. Calculated Kohn-Sham HOMO and LUMO orbitals for $\mathbf{A_2B}$ (PBE0-D3(BJ)/Def2-SVP).

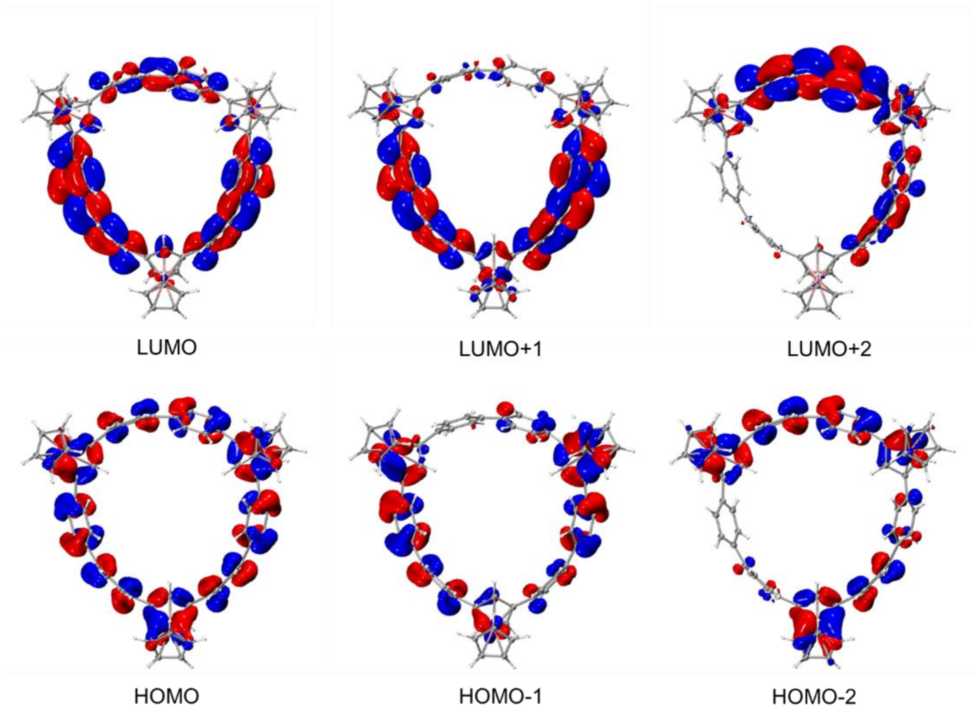

Figure S26. Calculated Kohn-Sham HOMO and LUMO orbitals for $\mathbf{A_3}$ (PBE0-D3(BJ)/Def2-SVP).

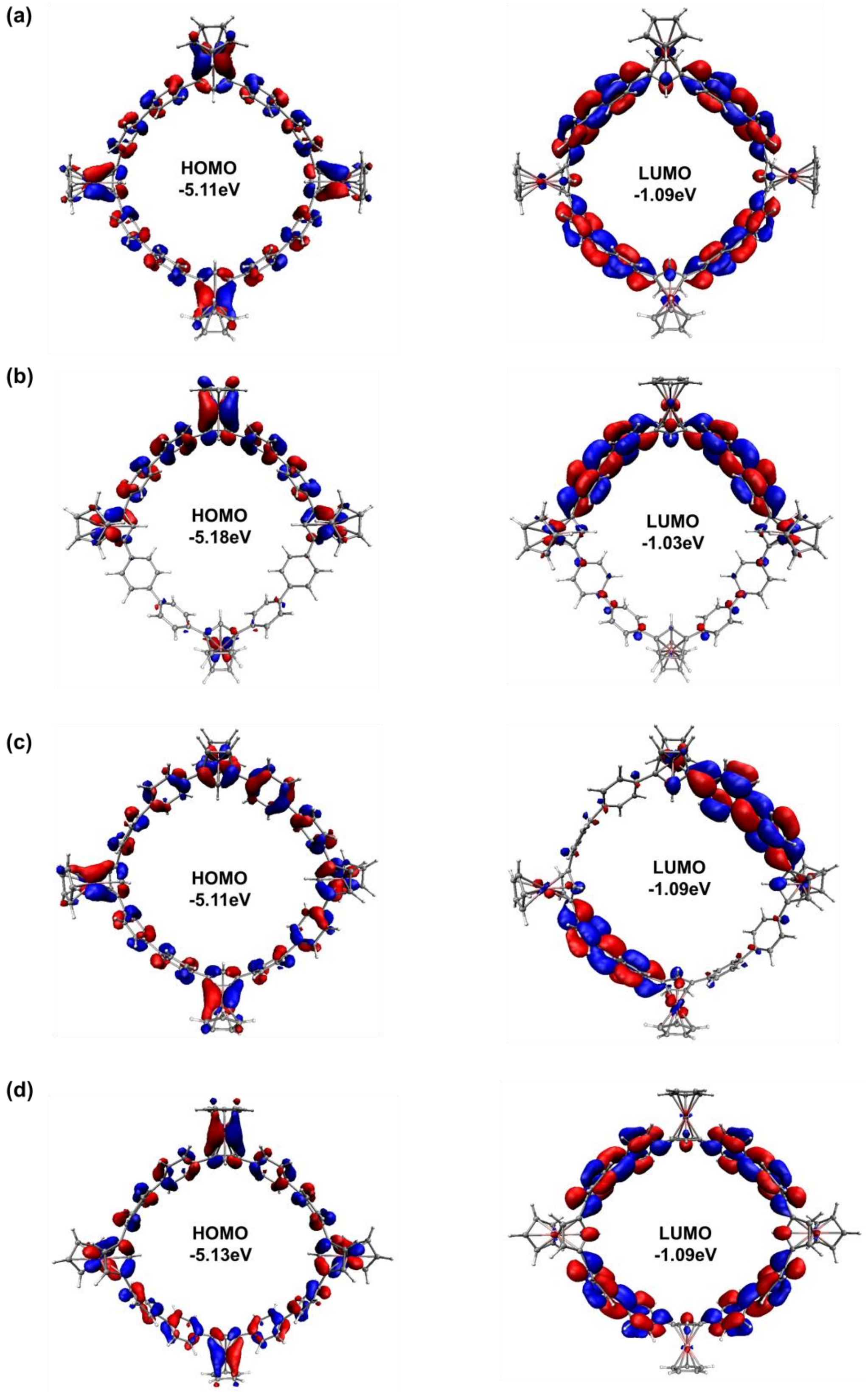

Figure S27. Calculated Kohn-Sham HOMO and LUMO orbitals for (a) **AAAA**, (b) **AAAB**, (c) **AABB** and (d) **ABAB** (B3LYP/SDD, 6-31G(d)).

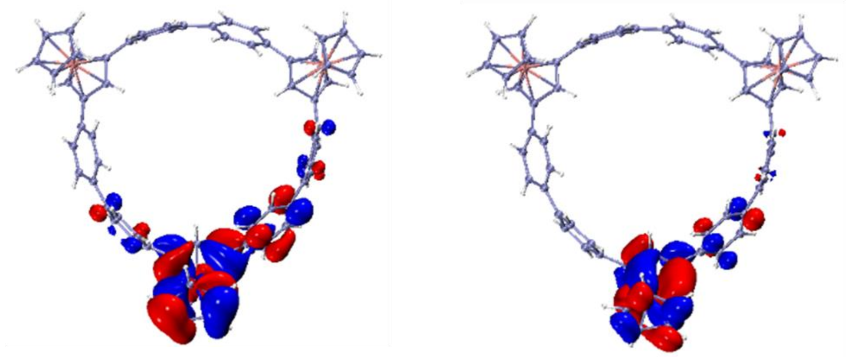

α-LUMO β-LUMO

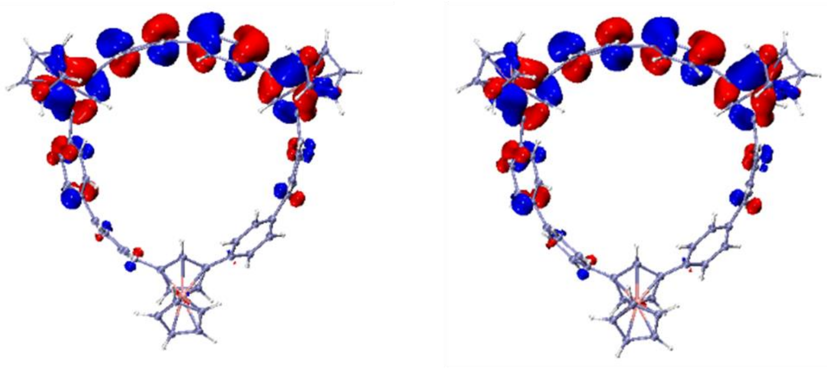

α-HOMO β-HOMO

Figure S28. Calculated Kohn-Sham highest occupied and lowest unoccupied molecular spin-orbitals of $\mathbf{A_3}^+$. (UPBE0-D3(BJ)/Def2-SVP)

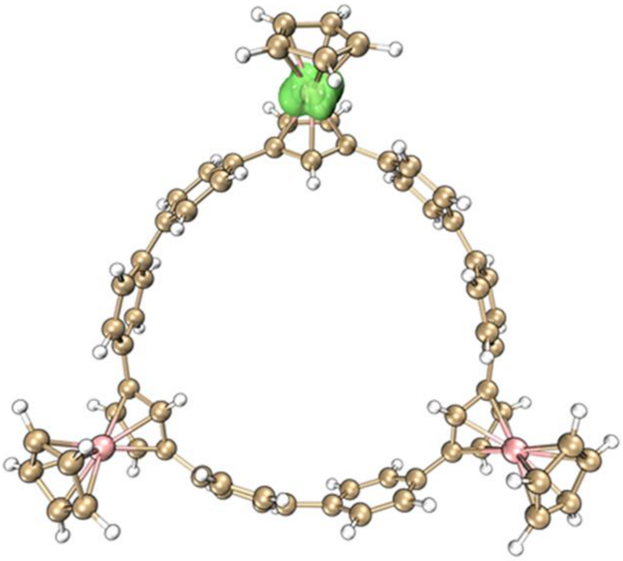

Figure S29. Spin density maps for $\mathbf{A_3}^+$.

6.7 Cartesian coordinates of optimized structures

A_2B (PBE0-D3(BJ)/Def2-SVP)

C	-8.44187	-1.63551	1.49166
C	-9.09813	-2.57427	0.63972
C	-8.53459	-3.86289	0.88601
C	-7.52959	-3.72096	1.89011
C	-7.47220	-2.34416	2.26523
C	-5.23916	-3.23782	-0.55825
C	-5.15372	-1.86655	-0.15622
C	-6.09050	-1.08417	-0.90317
C	-6.80015	-1.99428	-1.75237
C	-6.28457	-3.31036	-1.5376
Fe	-7.05878	-2.5524	0.23809
C	-3.56688	-4.1375	1.07291
C	-2.32953	-4.73316	1.28775
C	-1.67667	-5.44944	0.267
C	-2.39934	-5.67949	-0.91738
C	-3.63629	-5.0752	-1.13726
C	-4.20576	-4.22199	-0.17675
C	-5.60431	1.04645	-2.0906
C	-5.04042	2.31947	-2.05361
C	-4.85422	3.00326	-0.83779
C	-5.42129	2.42111	0.31223
C	-5.96377	1.13946	0.28115
C	-5.99185	0.3938	-0.90893
C	-0.06979	6.07596	-0.82083
C	0.95862	5.30138	-0.19672
C	2.24324	5.7448	-0.64342
C	2.00766	6.84362	-1.5341
C	0.59543	7.0475	-1.64061
Fe	1.20280	7.28038	0.33491
C	-2.33117	5.65791	-1.85185
C	-3.50710	4.90648	-1.85758
C	-3.88222	4.12535	-0.74892
C	-3.11580	4.27802	0.42266
C	-1.95648	5.04052	0.43621
C	-1.48848	5.67533	-0.72803
C	3.60133	4.34452	0.9146
C	4.44598	3.25597	1.09342
C	5.18983	2.72598	0.02263
C	5.17592	3.44593	-1.18572
C	4.32244	4.53419	-1.36872
C	3.45402	4.95002	-0.34566
C	3.91585	-4.66908	0.80732
C	4.21999	-3.41708	0.18495
C	5.42996	-2.88316	0.73146
C	5.90692	-3.84579	1.68165
C	4.98211	-4.93603	1.72821
Fe	5.75966	-4.73764	-0.19228
C	1.9672	-5.26905	-0.63688
C	0.59506	-5.45057	-0.75859
C	-0.21333	-5.68692	0.36916
C	0.44216	-5.89389	1.59613
C	1.81863	-5.70506	1.72073

C	2.59594	-5.30757	0.61987
C	6.1723	-0.61643	1.51429
C	6.11449	0.76688	1.3462
C	5.70604	1.3361	0.12651
C	5.53716	0.46244	-0.96399
C	5.60319	-0.91584	-0.7999
C	5.83201	-1.48629	0.46471
C	2.35219	7.94626	1.93309
C	2.10292	9.02076	1.0269
C	0.68948	9.20228	0.935
C	0.06443	8.23996	1.7843
C	1.09208	7.46396	2.40226
C	7.64731	-5.42956	-0.71642
C	6.72008	-6.51493	-0.679
C	5.68392	-6.24333	-1.62276
C	5.97138	-4.98989	-2.24452
C	7.18465	-4.48656	-1.68322
H	-8.62866	-0.5699	1.5231
H	-9.86739	-2.34344	-0.08584
H	-8.80084	-4.78166	0.37965
H	-6.8984	-4.51257	2.27295
H	-6.79582	-1.91046	2.99059
H	-4.42727	-1.46131	0.53538
H	-7.61122	-1.73262	-2.42057
H	-6.64257	-4.21413	-2.01496
H	-4.00561	-3.52264	1.85419
H	-1.81283	-4.54813	2.22505
H	-1.95755	-6.2801	-1.70815
H	-4.11909	-5.18832	-2.10449
H	-5.62932	0.50236	-3.03119
H	-4.63061	2.73157	-2.97079
H	-5.38091	2.94935	1.26058
H	-6.31059	0.68052	1.20288
H	0.79142	4.44588	0.44305
H	2.76963	7.44126	-2.01888
H	0.11343	7.82581	-2.21909
H	-2.01944	6.1627	-2.76271
H	-4.10256	4.87555	-2.76594
H	-3.37187	3.71035	1.31146
H	-1.35489	5.07741	1.34015
H	2.97515	4.67659	1.73815
H	4.44313	2.74034	2.04924
H	5.7845	3.1071	-2.02008
H	4.2579	5.00219	-2.34776
H	3.58067	-2.90637	-0.52227
H	6.82825	-3.77548	2.24644
H	5.08736	-5.82758	2.33391
H	2.55198	-5.00671	-1.51435
H	0.12896	-5.29969	-1.72786
H	-0.1394	-6.13746	2.48149
H	2.27918	-5.77304	2.70303
H	6.38685	-1.02481	2.49857
H	6.31735	1.4104	2.19828
H	5.23947	0.8629	-1.92866
H	5.38431	-1.56504	-1.64335
H	3.32414	7.54573	2.19095
H	2.85343	9.579	0.48228

H	0.17933	9.92261	0.30859
H	-1.00168	8.10009	1.90877
H	0.94137	6.63967	3.08756
H	8.53017	-5.32478	-0.09909
H	6.77579	-7.37802	-0.02825
H	4.81594	-6.86285	-1.80834
H	5.36376	-4.49644	-2.99227
H	7.65295	-3.54038	-1.92203

A_3 (PBE0-D3(BJ)/Def2-SVP)

C	-6.80052	-4.60602	1.8016
C	-7.06529	-5.81362	1.0935
C	-5.9513	-6.68403	1.275
C	-4.99679	-6.01417	2.09434
C	-5.52132	-4.72915	2.42052
C	-3.58957	-4.91266	-0.63211
C	-4.00542	-3.59622	-0.26663
C	-5.2805	-3.32217	-0.84517
C	-5.67042	-4.49147	-1.56834
C	-4.63718	-5.46829	-1.43309
Fe	-5.356	-4.94444	0.40059
C	-1.55155	-4.92689	0.78915
C	-0.16983	-4.99249	0.8785
C	0.60678	-5.53732	-0.15828
C	-0.08664	-6.15756	-1.21022
C	-1.4727	-6.07485	-1.3121
C	-2.22518	-5.38356	-0.35334
C	-5.79982	-1.17563	-1.98189
C	-5.79244	0.21426	-1.92499
C	-5.79883	0.8943	-0.69636
C	-5.99652	0.12003	0.45906
C	-5.98051	-1.26812	0.40665
C	-5.79842	-1.94077	-0.80861
C	-2.57433	5.54556	-0.77296
C	-1.28956	5.11917	-0.32085
C	-0.30481	6.08159	-0.69638
C	-0.99358	7.12821	-1.38739
C	-2.38397	6.79965	-1.43451
Fe	-1.73977	6.9112	0.49955
C	-4.5554	4.32154	-1.73186
C	-5.35005	3.17674	-1.71123
C	-5.3383	2.30007	-0.61445
C	-4.63291	2.71843	0.52637
C	-3.85505	3.8645	0.51401
C	-3.72871	4.64099	-0.647
C	1.51331	5.09864	0.66665
C	2.72544	4.43039	0.72496
C	3.61731	4.43626	-0.36069
C	3.30079	5.28048	-1.4374
C	2.07454	5.93851	-1.50622
C	1.12264	5.7906	-0.48925
C	6.19546	-0.55961	-0.7584
C	5.26517	-1.47479	-0.17821
C	5.51018	-2.78438	-0.6922
C	6.62392	-2.68143	-1.5825
C	7.04336	-1.31824	-1.62355
Fe	7.14807	-2.04067	0.28488
C	3.90745	-4.44333	-1.60162
C	2.6916	-5.10492	-1.47317
C	2.05391	-5.22854	-0.22823
C	2.7772	-4.80752	0.90106
C	3.98321	-4.12968	0.77389
C	4.53049	-3.86402	-0.48898
C	5.75282	1.48788	0.62074
C	5.12021	2.72118	0.71336

C	4.67258	3.39915	-0.43358
C	5.06771	2.87709	-1.67563
C	5.71944	1.65255	-1.77089
C	5.98917	0.89241	-0.62572
C	-0.78124	7.65199	2.1368
C	-1.4531	8.69656	1.43822
C	-2.8412	8.3748	1.39215
C	-3.02814	7.13121	2.06215
C	-1.75489	6.68459	2.524
C	7.32906	-2.02467	2.31439
C	7.57451	-3.3324	1.80145
C	8.66927	-3.25068	0.89271
C	9.10114	-1.89315	0.84361
C	8.27346	-1.13537	1.72195
H	-7.45438	-3.73603	1.83983
H	-7.95291	-6.02307	0.49867
H	-5.836	-7.6762	0.84151
H	-4.02195	-6.40322	2.38587
H	-5.02313	-3.96873	3.02043
H	-3.41667	-2.87826	0.30117
H	-6.60914	-4.62488	-2.10426
H	-4.65732	-6.47635	-1.8454
H	-2.11896	-4.43365	1.58192
H	0.31959	-4.51927	1.7316
H	0.46948	-6.65359	-2.00885
H	-1.97013	-6.47533	-2.19896
H	-5.67597	-1.67591	-2.94561
H	-5.65453	0.774	-2.85247
H	-6.09072	0.60919	1.43096
H	-6.03112	-1.84763	1.33065
H	-1.0833	4.17132	0.17225
H	-0.54224	8.03823	-1.7806
H	-3.16854	7.41745	-1.86948
H	-4.50607	4.92585	-2.64124
H	-5.93346	2.92513	-2.60004
H	-4.60815	2.08146	1.41216
H	-3.2578	4.12192	1.39162
H	0.80959	5.01356	1.49782
H	2.93559	3.80776	1.5963
H	3.99253	5.36938	-2.27824
H	1.81603	6.50107	-2.40693
H	4.44708	-1.20238	0.48658
H	7.08859	-3.5099	-2.1157
H	7.88391	-0.92439	-2.19355
H	4.32025	-4.26489	-2.59757
H	2.17599	-5.42633	-2.3802
H	2.35918	-4.95413	1.89933
H	4.48114	-3.73514	1.66302
H	6.00554	0.93687	1.52977
H	4.90894	3.13121	1.70333
H	4.7549	3.37416	-2.59609
H	5.90917	1.21897	-2.75591
H	0.2922	7.58817	2.31085
H	-0.98306	9.57263	0.99419
H	-3.61959	8.96169	0.90708
H	-3.97338	6.60073	2.17001
H	-1.55785	5.75848	3.06271

H	6.54506	-1.7499	3.01874
H	7.00829	-4.23269	2.03626
H	9.0848	-4.07696	0.31811
H	9.90517	-1.49798	0.2248
H	8.33659	-0.06054	1.88623

7. Variable Temperature NMR Spectroscopy

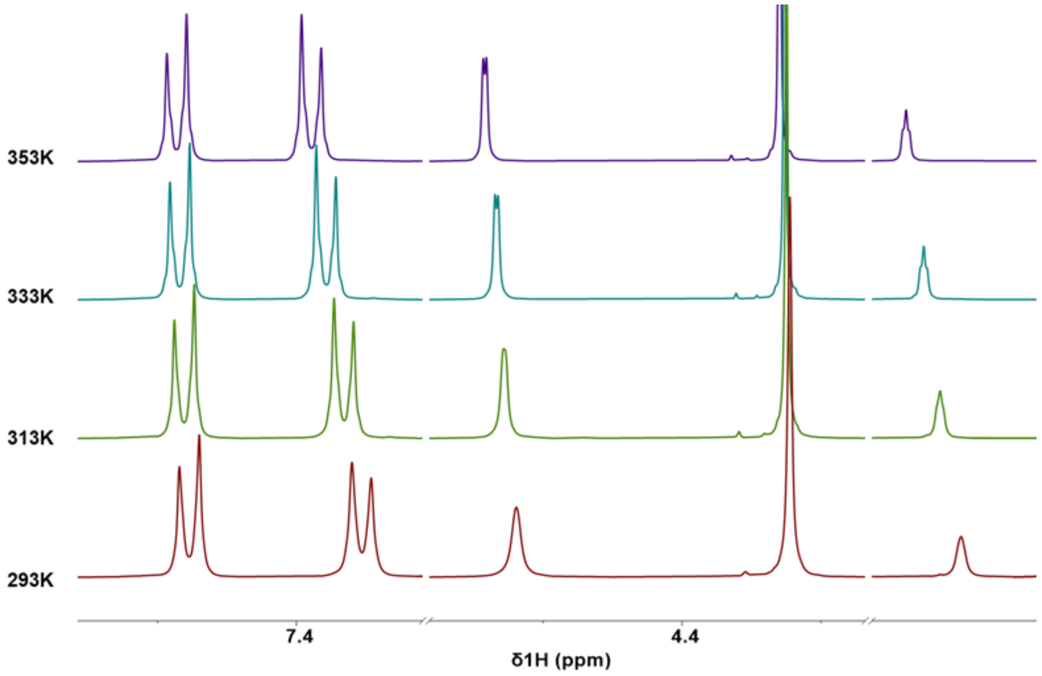

Figure S30. ^{1}H NMR variable temperature experiment of **A**$_3$ in 1,1,2,2-tetrachloroethane-d_2 (500 MHz).

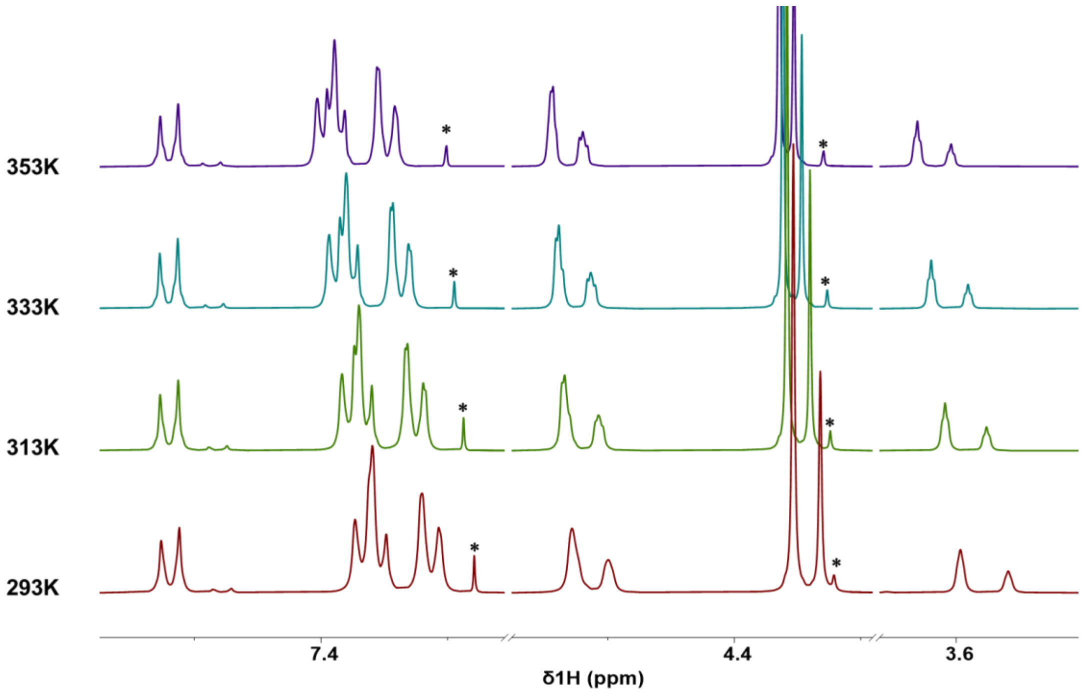

Figure S31. ^{1}H NMR variable temperature experiment of **A**$_2$**B** in 1,1,2,2-tetrachloroethane-d_2 (500 MHz).

8. UV/VIS/NIR SPECTROSCOPY

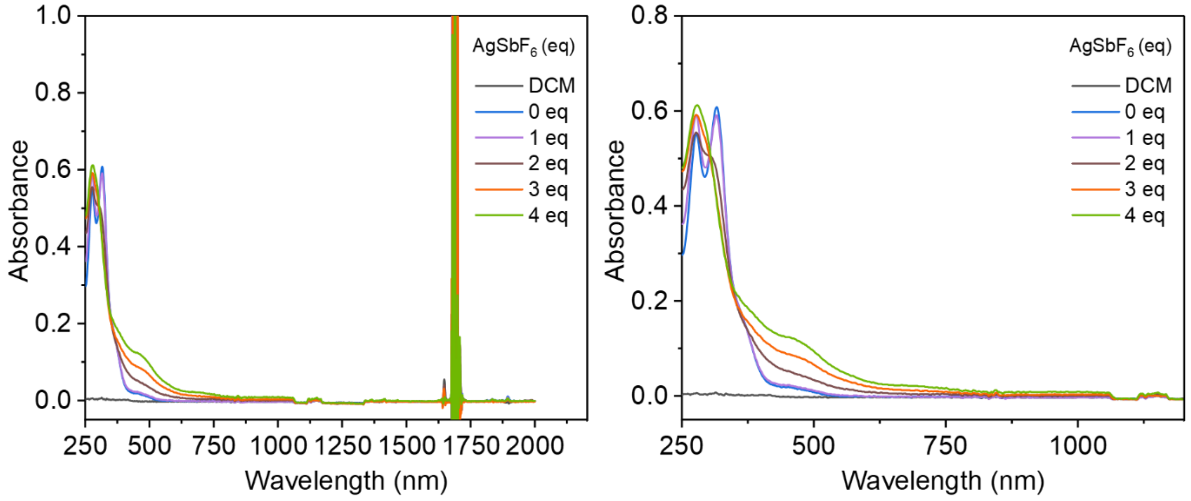

Figure S32. UV/Vis/NIR spectra of oxidation of **A$_2$B** to **A$_2$B^{3+}** via the addition of $AgSbF_6$ to a solution of **A$_2$B** in CH_2Cl_2 containing *n*-Bu_4NBArF.

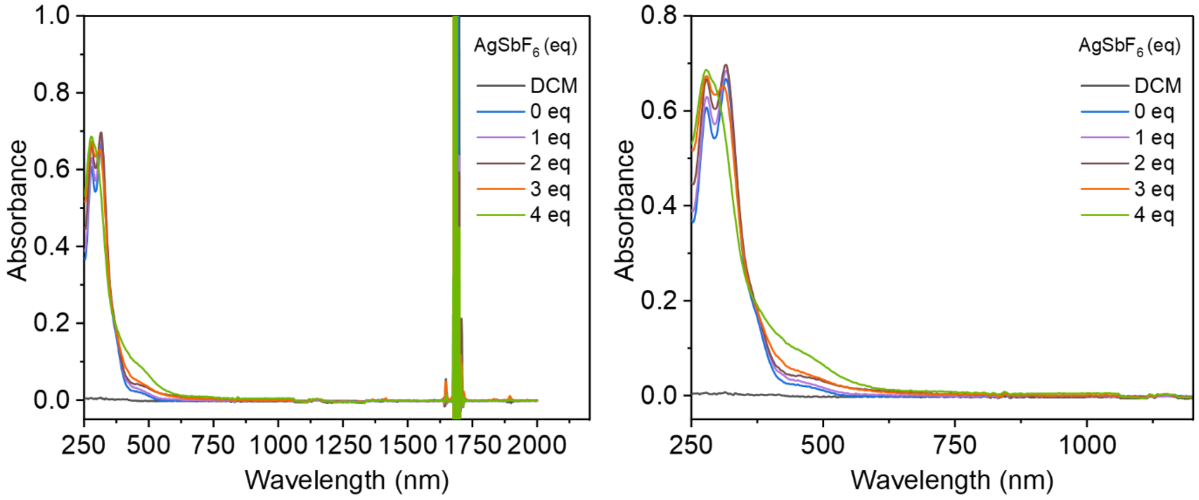

Figure S33. UV/Vis/NIR spectra of oxidation of **A$_3$** to **A$_3$$^{3+}$** via the addition of $AgSbF_6$ to a solution of **A$_3$** in CH_2Cl_2 containing *n*-Bu_4NBArF.

9. References

(1) Dolomanov, O. V.; Bourhis, L. J.; Gildea, R. J.; Howard, J. A. K.; Puschmann, H., OLEX2: a complete structure solution, refinement and analysis program. *J. Appl. Crystallogr.* **2009,** *42*, 339-341.

(2) van der Sluis, P.; Spek, A. L., BYPASS: an effective method for the refinement of crystal structures containing disordered solvent regions. *Acta Cryst.* **1990,** *46*, 194-201.

(3) Zirakzadeh, A.; Herlein, A.; Groß, M. A.; Mereiter, K.; Wang, Y.; Weissensteiner, W., Halide-Mediated Ortho-Deprotonation Reactions Applied to the Synthesis of 1,2- and 1,3-Disubstituted Ferrocene Derivatives. *Organometallics.* **2015,** *34*, 3820-3832.

(4) Farney, E. P.; Chapman, S. J.; Swords, W. B.; Torelli, M. D.; Hamers, R. J.; Yoon, T. P., Discovery and Elucidation of Counteranion Dependence in Photoredox Catalysis. *J. Am. Chem. Soc.* **2019,** *141*, 6385-6391.

(5) Duan, Y.; Ju, C.; Yang, G.; Fron, E.; Coutino-Gonzalez, E.; Semin, S.; Fan, C.; Balok, R. S.; Cremers, J.; Tinnemans, P.; Feng, Y.; Li, Y.; Hofkens, J.; Rowan, A. E.; Rasing, T.; Xu, J., Aggregation Induced Enhancement of Linear and Nonlinear Optical Emission from a Hexaphenylene Derivative. *Adv. Funct. Mater.* **2016,** *26*, 8968-8977.

(6) Gaussian 16, Revision C.01, M. J. Frisch, G. W. Trucks, H. B. Schlegel, G. E. Scuseria, M. A. Robb, J. R. Cheeseman, G. Scalmani, V. Barone, G. A. Petersson, H. Nakatsuji, X. Li, M. Caricato, A. V. Marenich, J. Bloino, B. G. Janesko, R. Gomperts, B. Mennucci, H. P. Hratchian, J. V. Ortiz, A. F. Izmaylov, J. L. Sonnenberg, D. Williams-Young, F. Ding, F. Lipparini, F. Egidi, J. Goings, B. Peng, A. Petrone, T. Henderson, D. Ranasinghe, V. G. Zakrzewski, J. Gao, N. Rega, G. Zheng, W. Liang, M. Hada, M. Ehara, K. Toyota, R. Fukuda, J. Hasegawa, M. Ishida, T. Nakajima, Y. Honda, O. Kitao, H. Nakai, T. Vreven, K. Throssell, J. A. Montgomery, Jr., J. E. Peralta, F. Ogliaro, M. J. Bearpark, J. J. Heyd, E. N. Brothers, K. N. Kudin, V. N. Staroverov, T. A. Keith, R. Kobayashi, J. Normand, K. Raghavachari, A. P. Rendell, J. C. Burant, S. S. Iyengar, J. Tomasi, M. Cossi, J. M. Millam, M. Klene, C. Adamo, R. Cammi, J. W. Ochterski, R. L. Martin, K. Morokuma, O. Farkas, J. B. Foresman, and D. J. Fox, Gaussian, Inc., Wallingford CT, 2016.

(7) Dolg, M.; Wedig, U.; Stoll, H.; Preuss, H., Energy‐adjusted ab initio pseudopotentials for the first row transition elements. *J. Chem. Phys.* **1987,** *86*, 866-872.

10. Mass Spectra

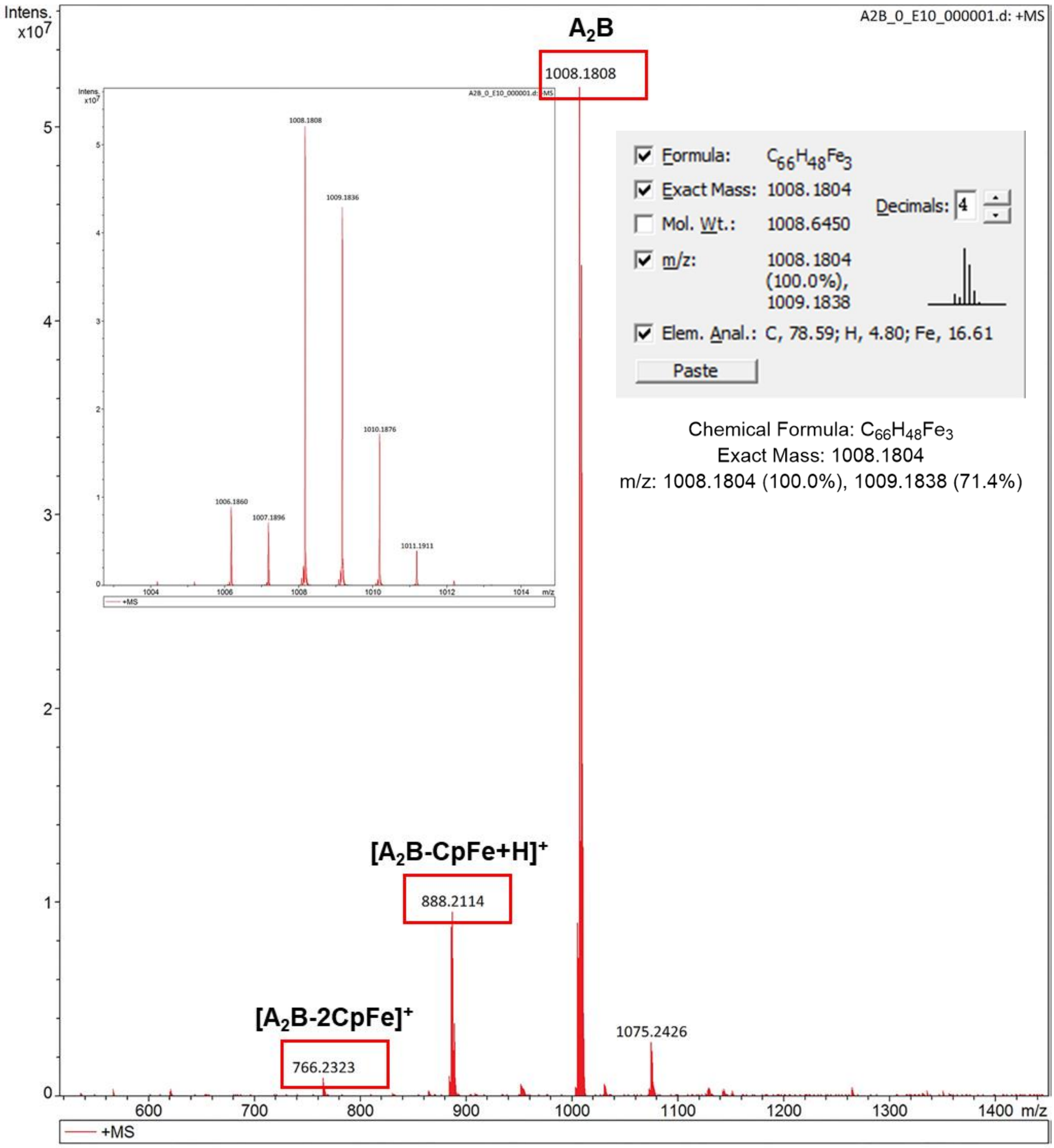

Figure S34. FTICR-MS of **A$_2$B**

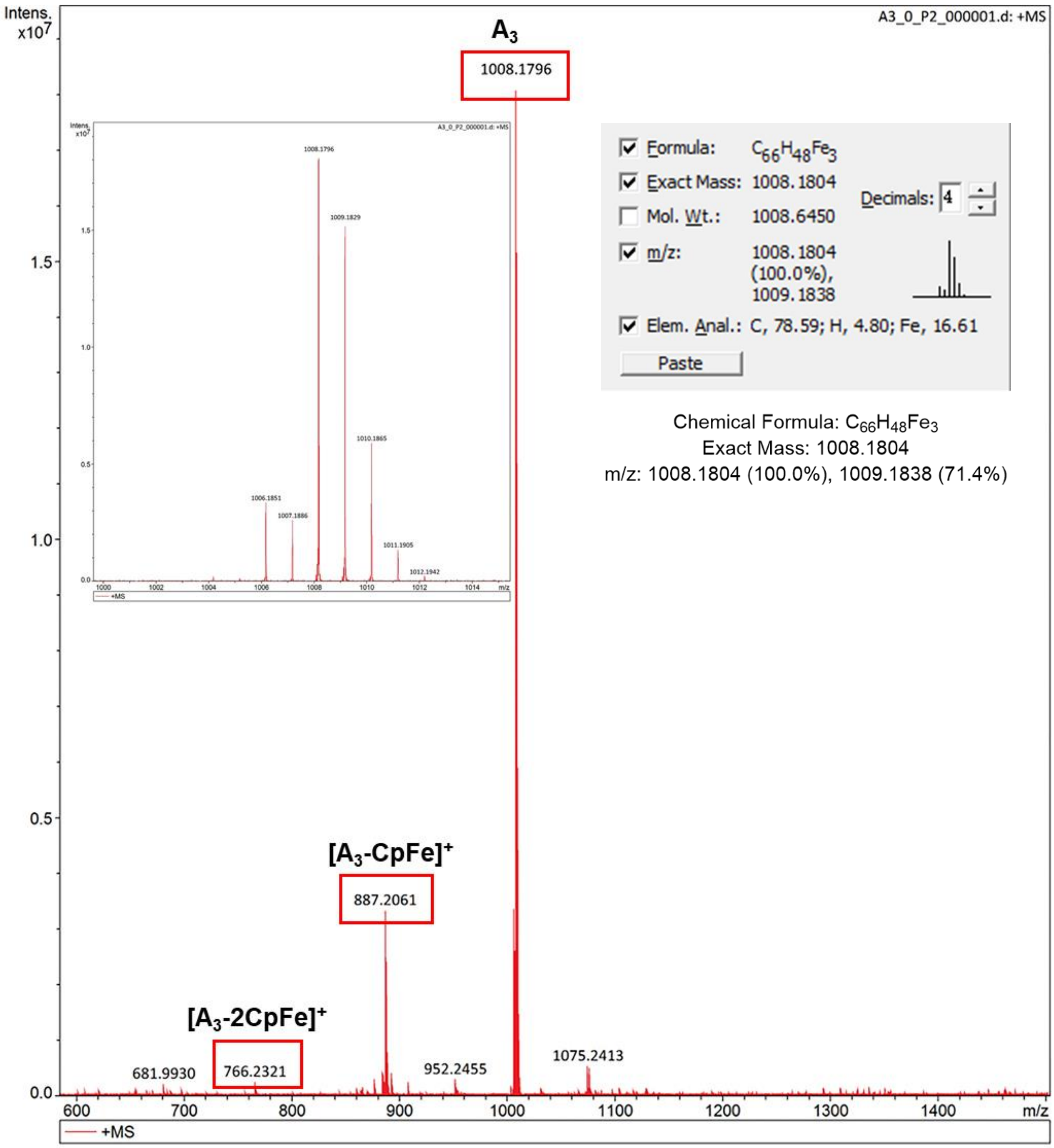

Figure S35. FTICR-MS of **A**$_3$

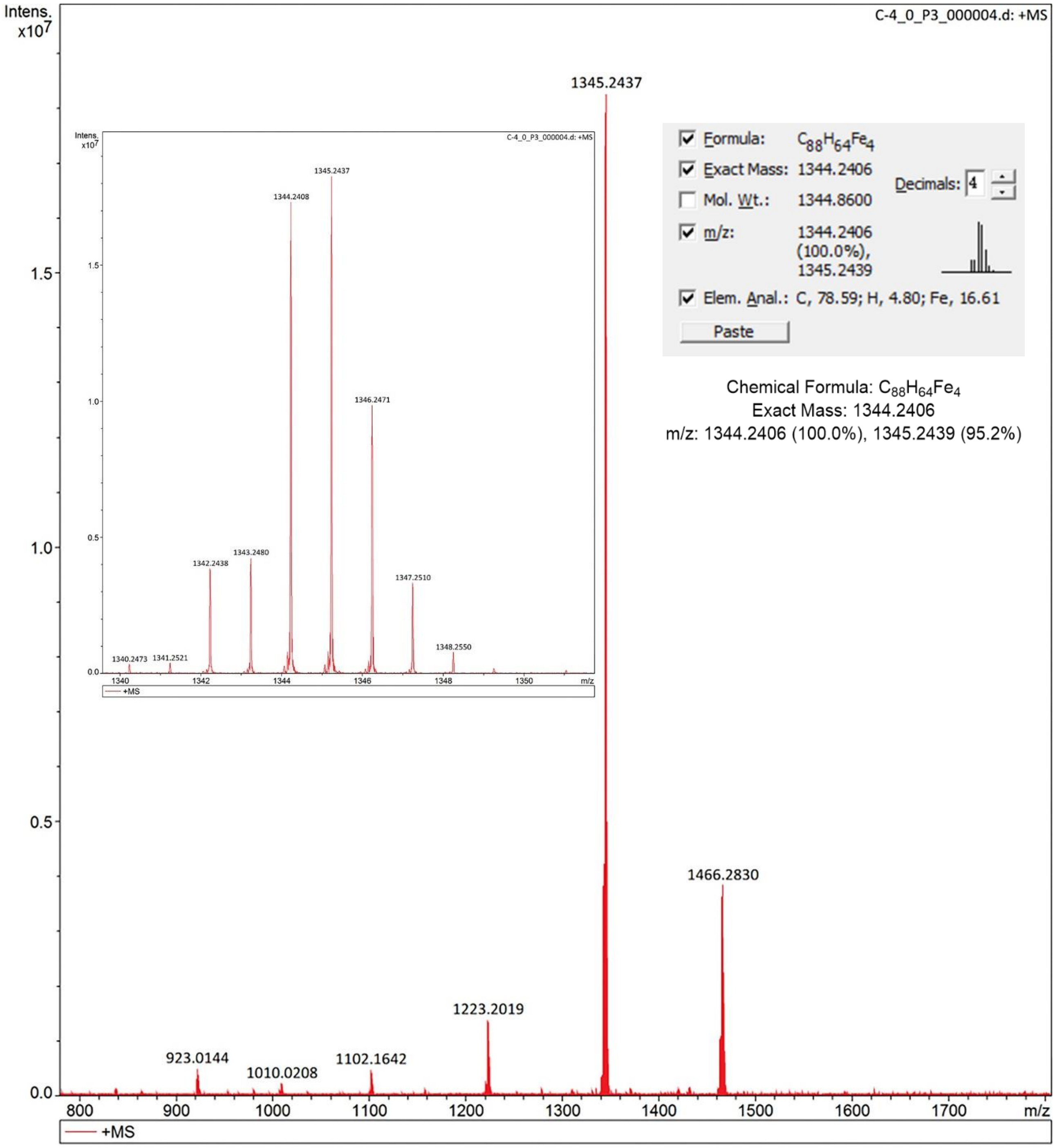

Figure S36. FTICR-MS of **Fc**$_4$

11. NMR Spectra

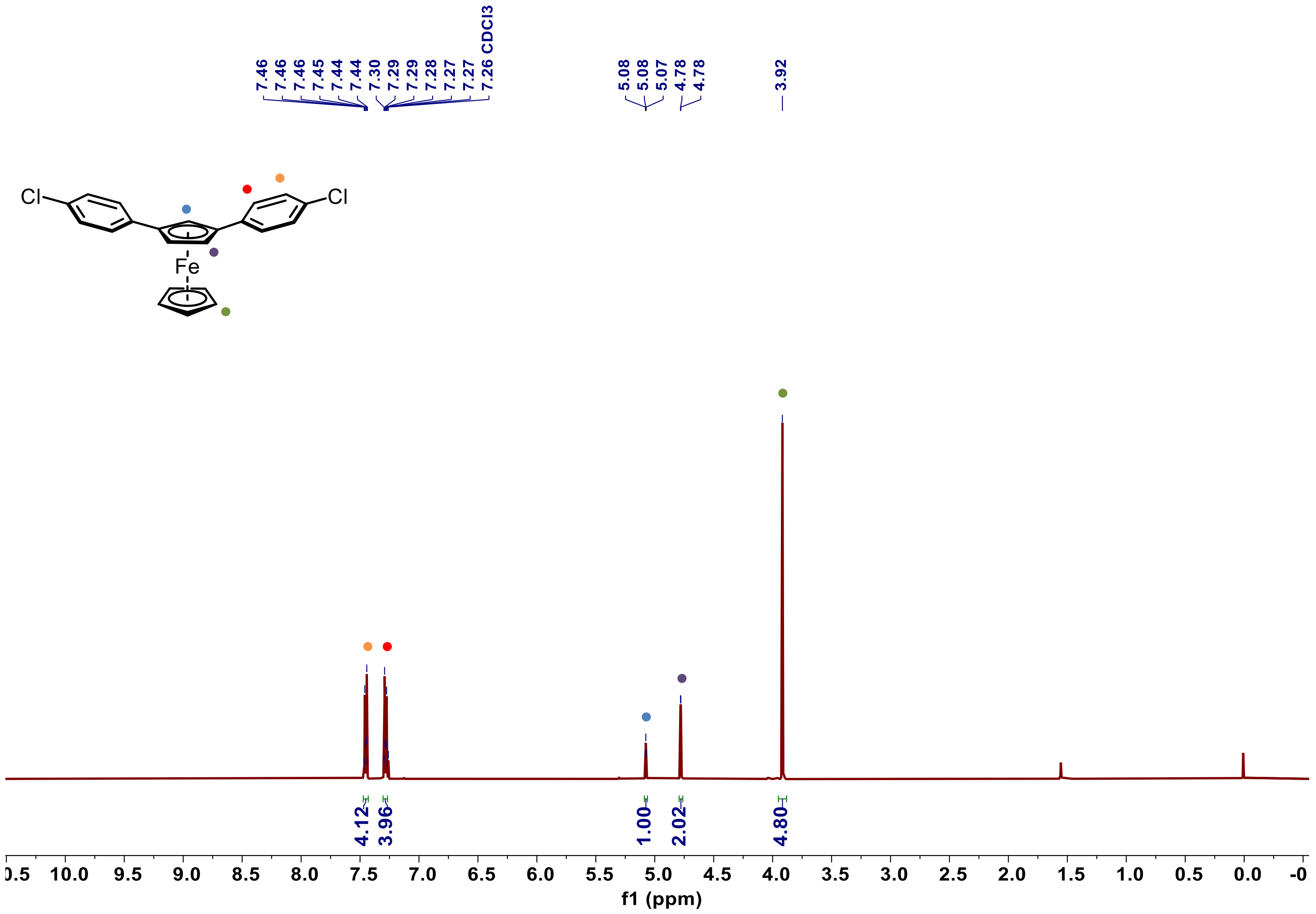

Figure S37. ^{1}H NMR-spectrum of **2** in $CDCl_3$ (500 MHz).

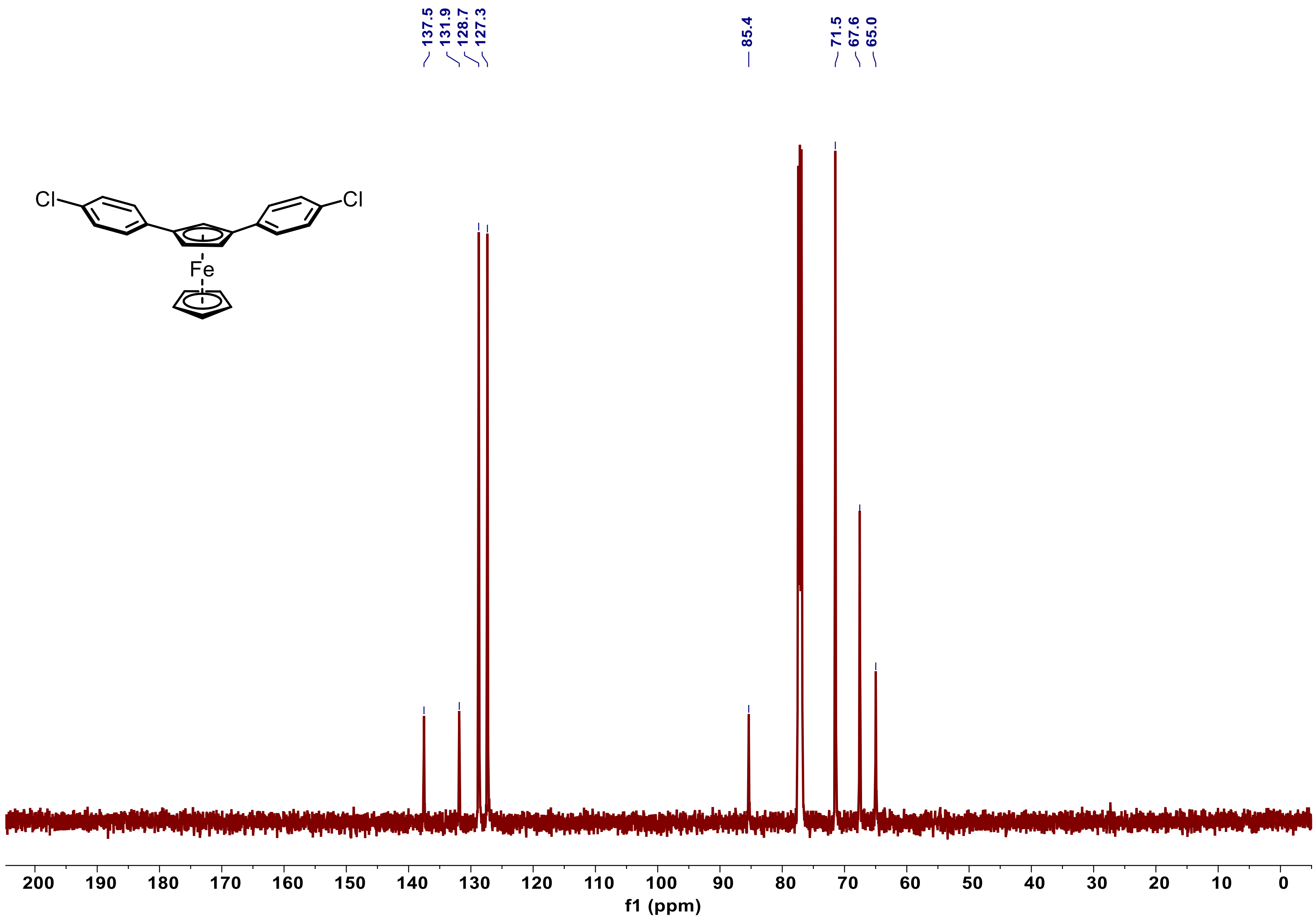

Figure S38. $^{13}C\{^{1}H\}$ NMR-spectrum of **2** in $CDCl_3$ (126 MHz).

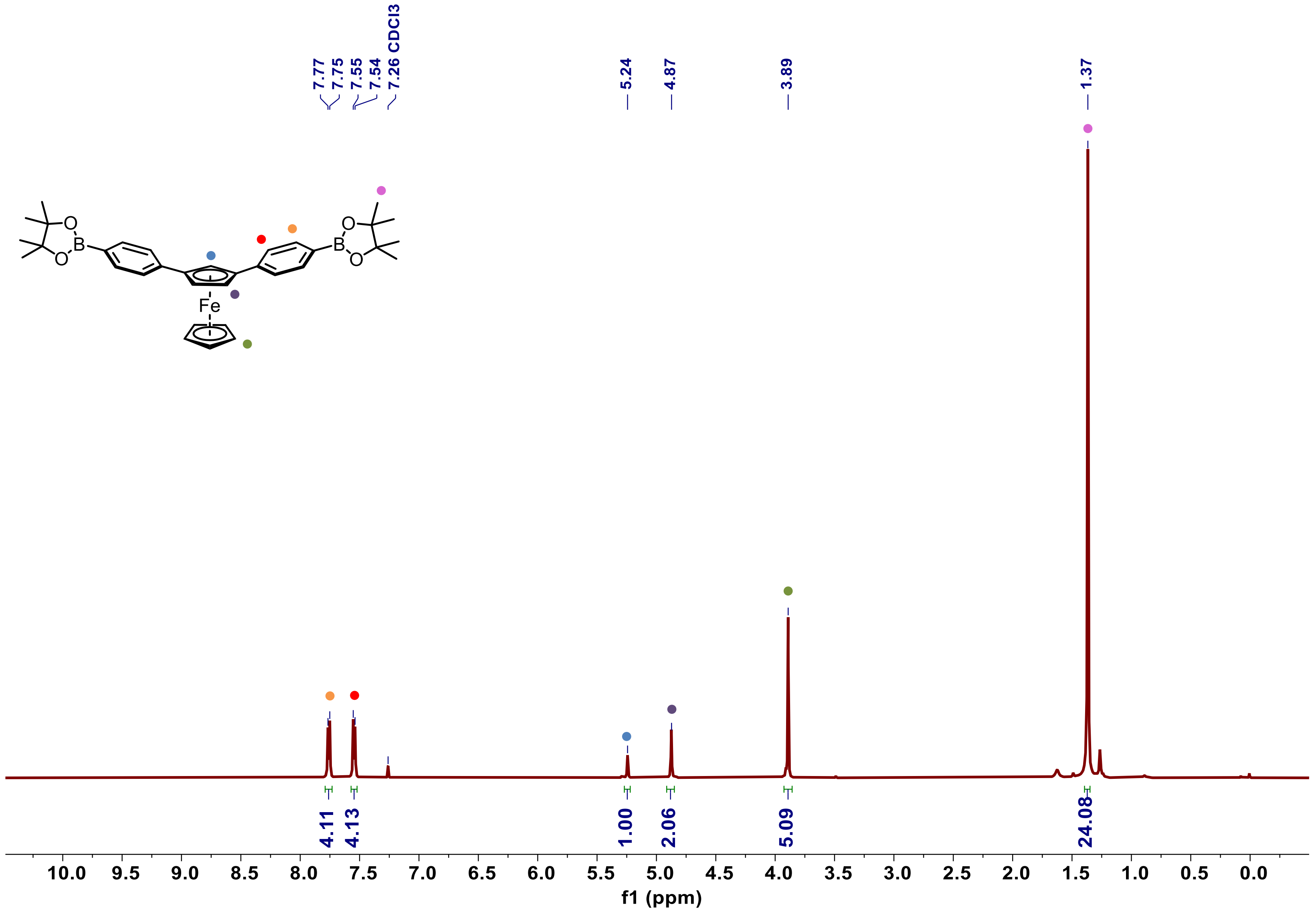

Figure S39. ^{1}H NMR-spectrum of **3** in $CDCl_3$ (500 MHz).

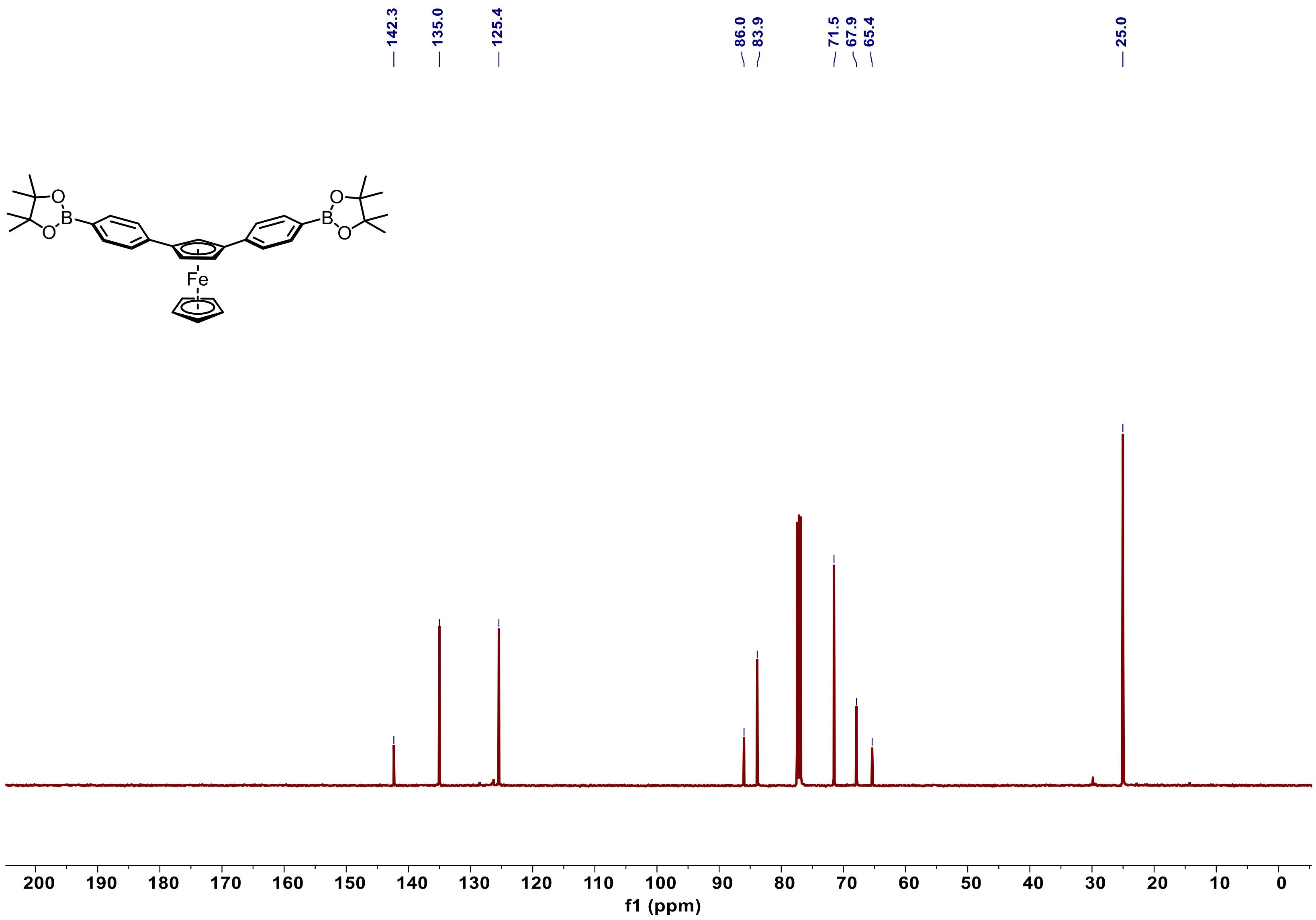

Figure S40. $^{13}C\{^{1}H\}$ NMR-spectrum of **3** in $CDCl_3$ (126 MHz).

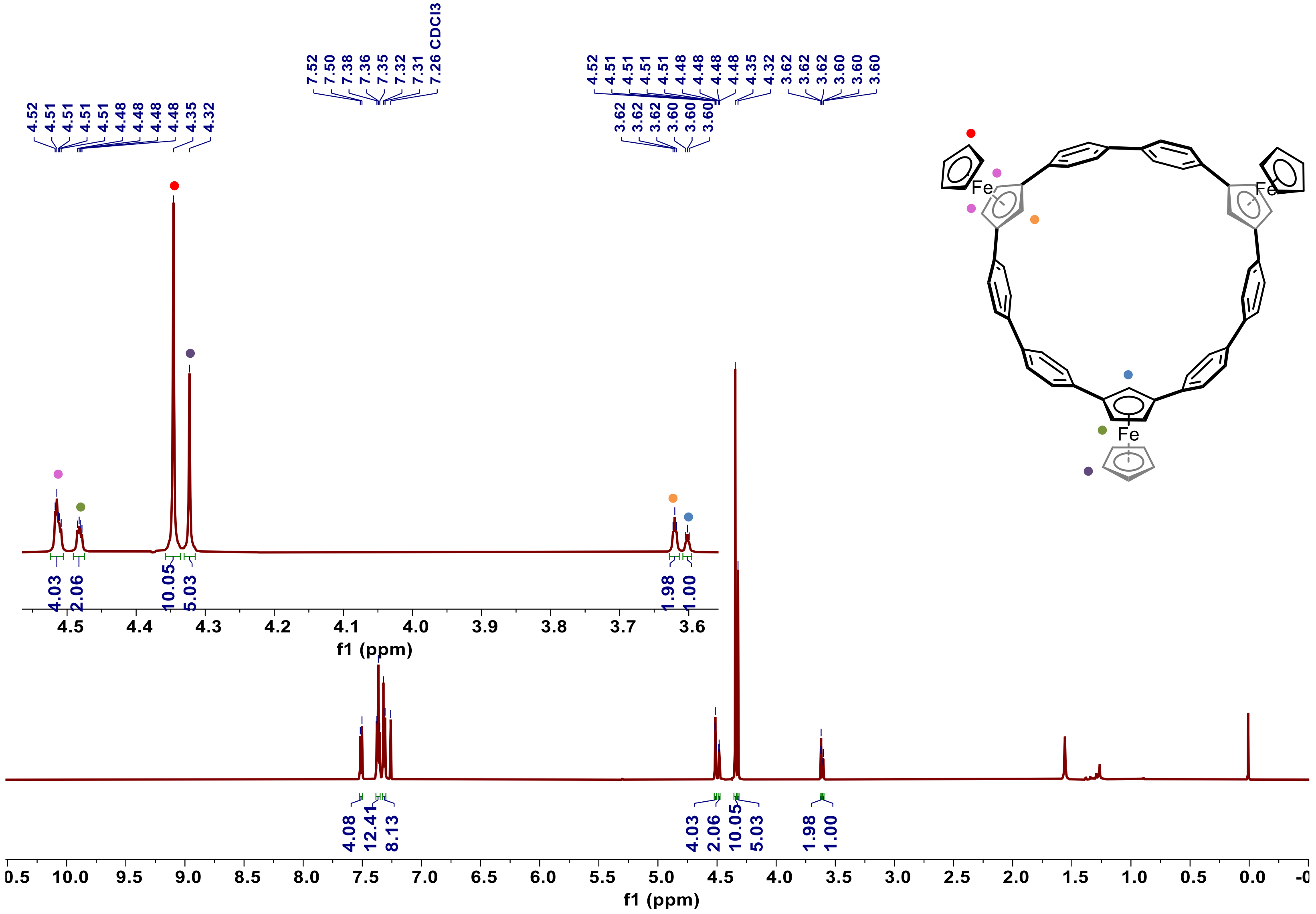

Figure S41. ^{1}H NMR-spectrum of **A$_2$B** in $CDCl_3$ (600 MHz).

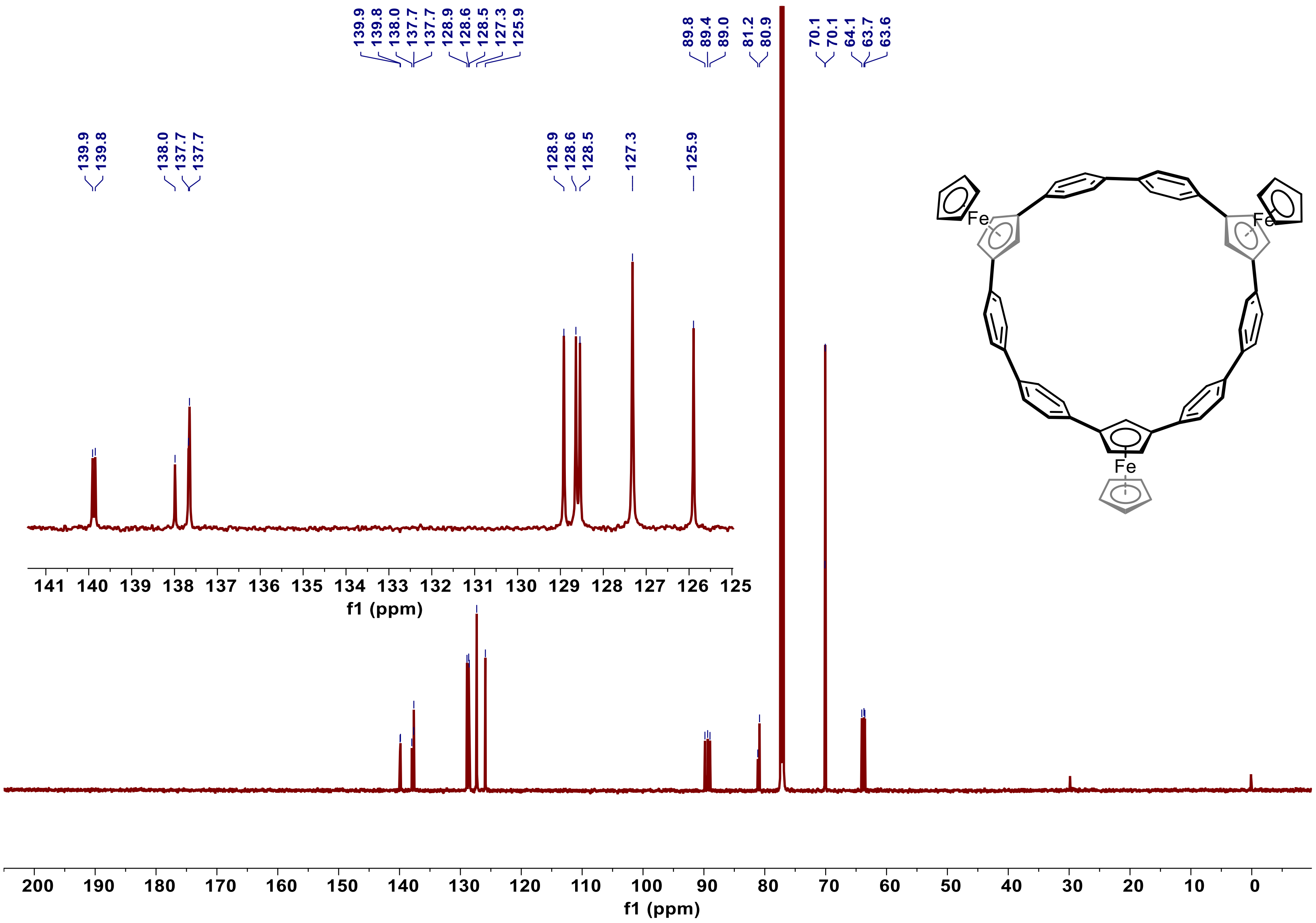

Figure S42. $^{13}C\{^{1}H\}$ NMR-spectrum of **A_2B** in $CDCl_3$ (151 MHz).

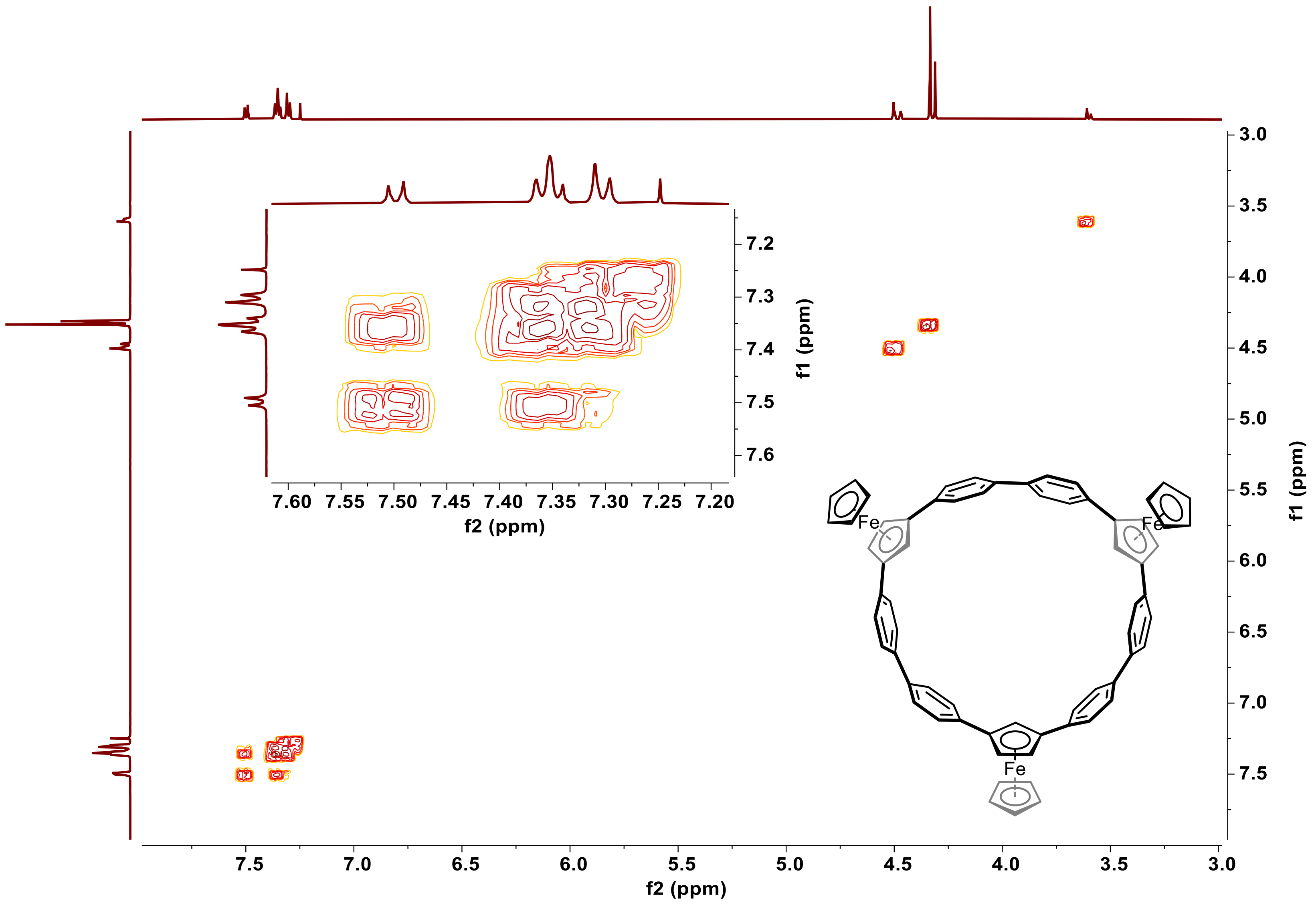

Figure S43. COSY-spectrum of **A$_2$B** in $CDCl_3$ (500 MHz).

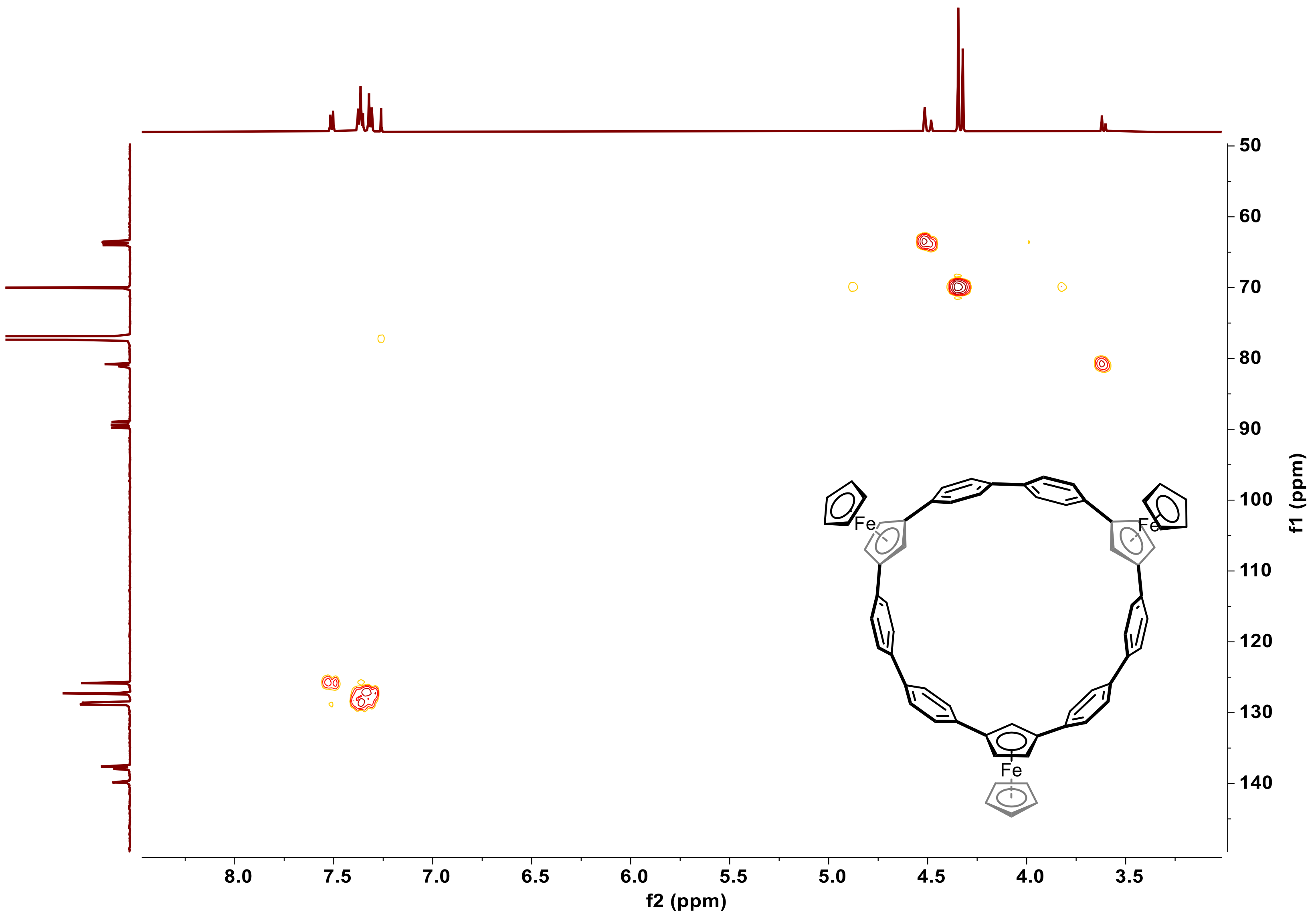

Figure S44. HMQC-spectrum of **A$_2$B** in $CDCl_3$ (500/126 MHz).

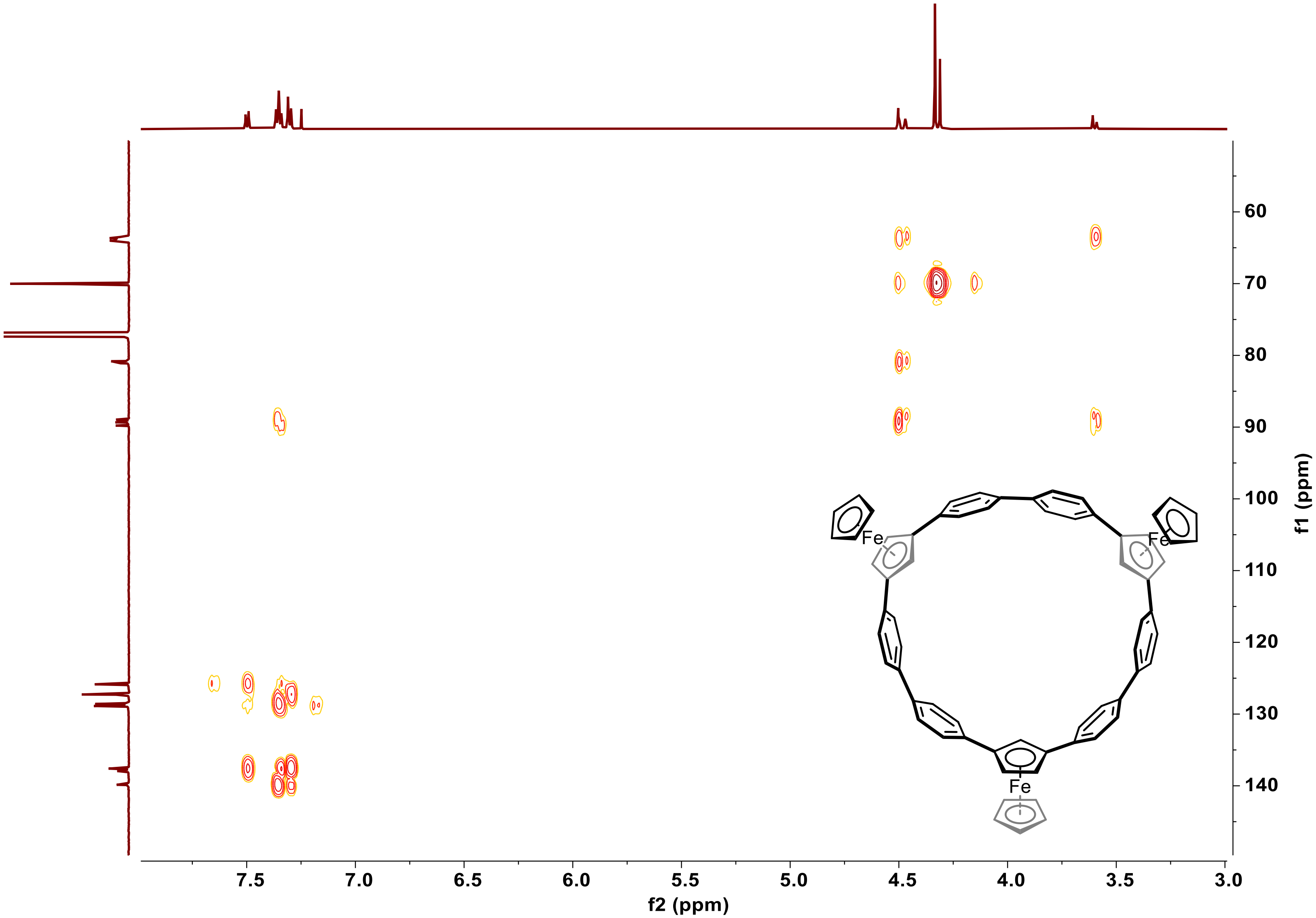

Figure S45. HMBC-spectrum of **A$_2$B** in $CDCl_3$ (500/126 MHz).

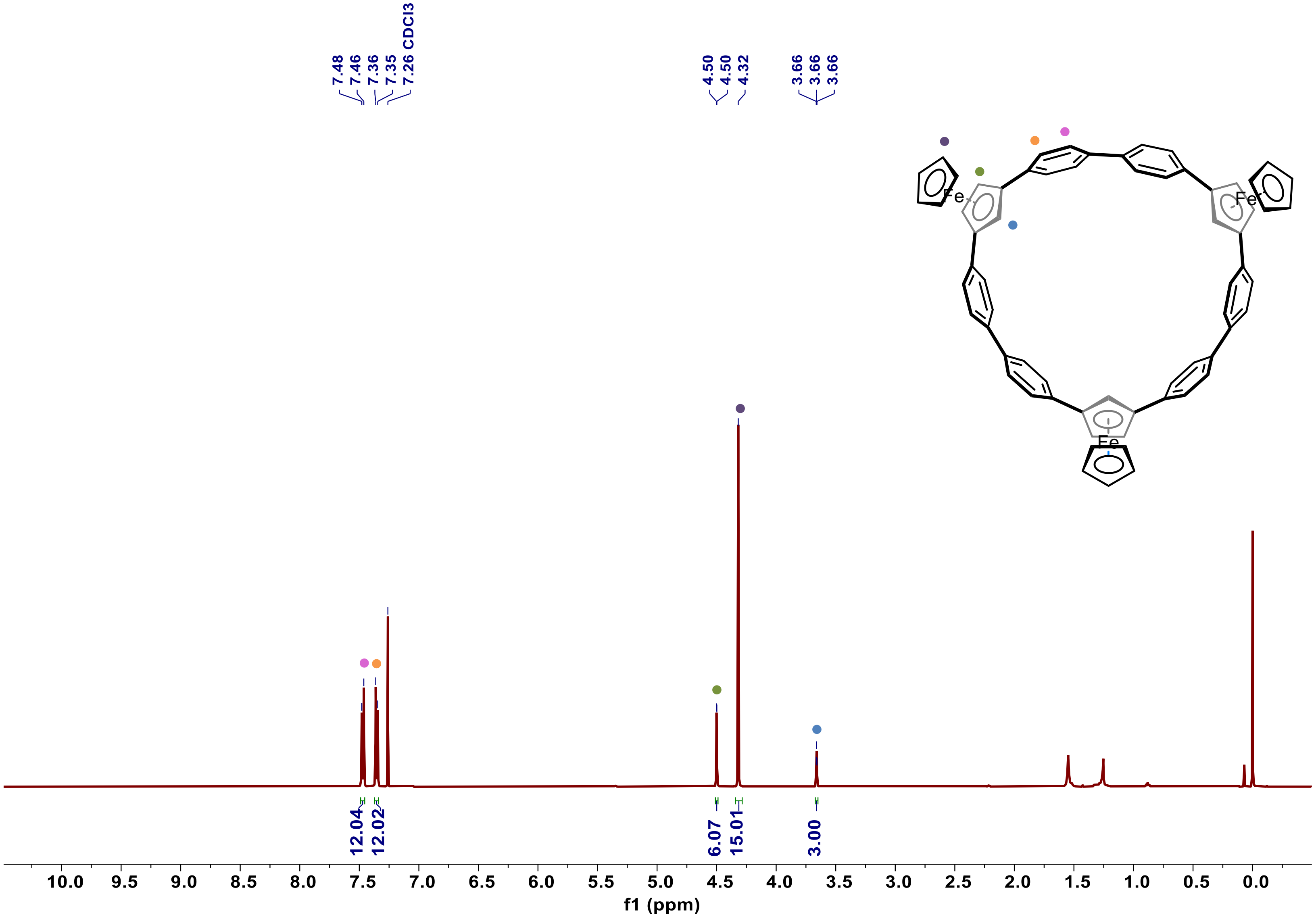

Figure S46. ^{1}H NMR-spectrum of **A$_3$** in $CDCl_3$ (600 MHz).

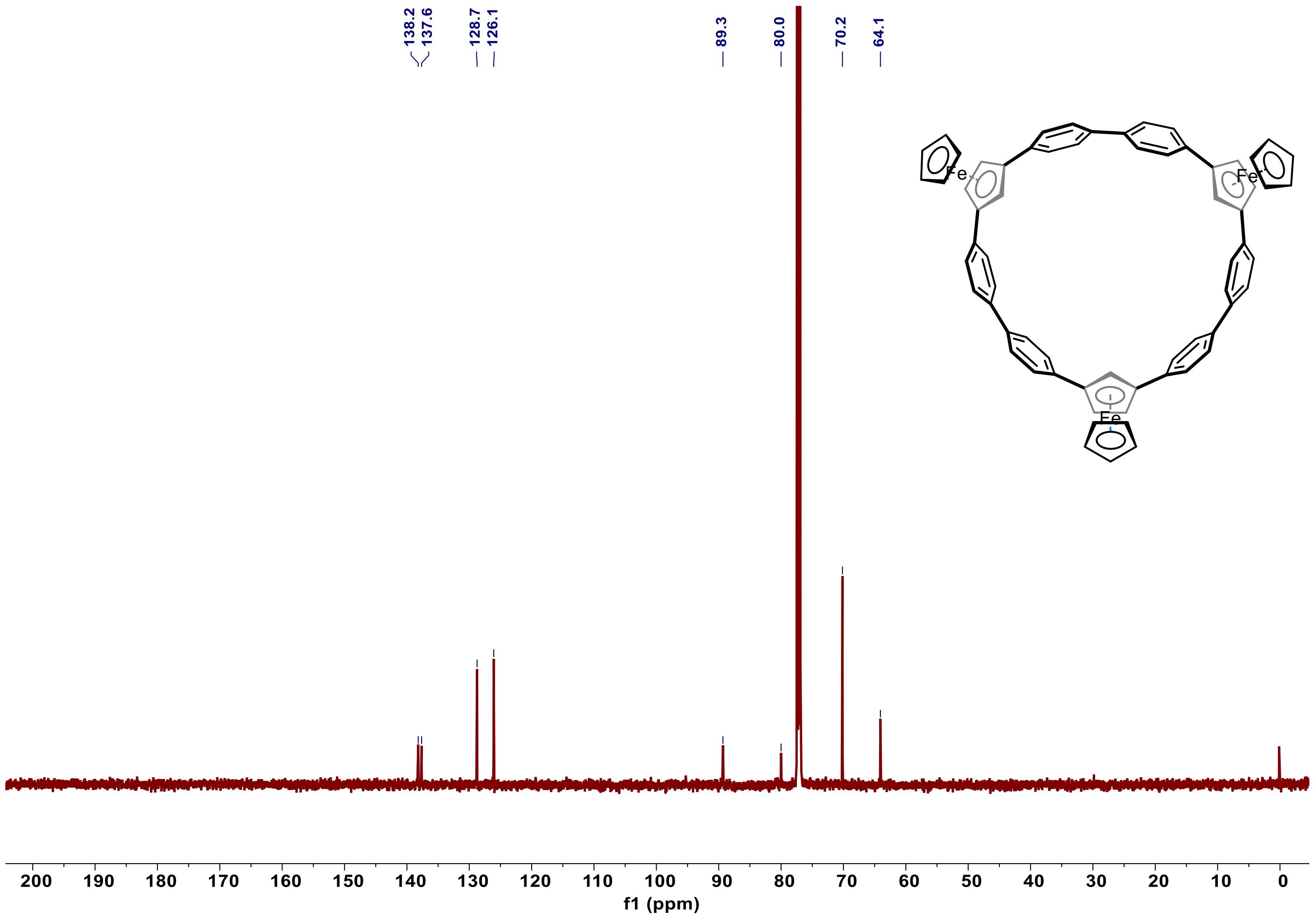

Figure S47. $^{13}C\{^{1}H\}$ NMR-spectrum of $\mathbf{A_3}$ in $CDCl_3$ (151 MHz).

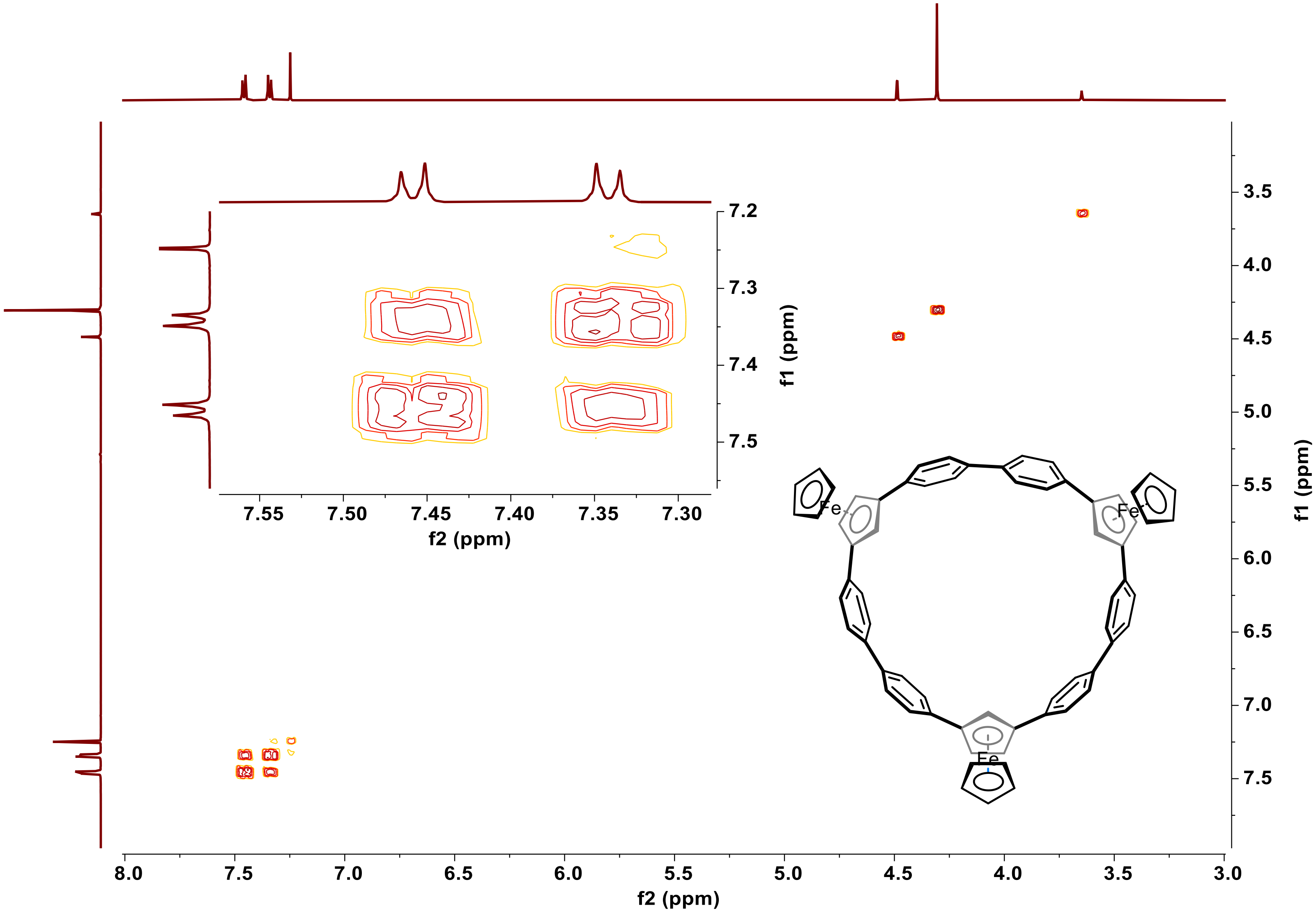

Figure S48. COSY-spectrum of **A**$_3$ in $CDCl_3$ (500 MHz).

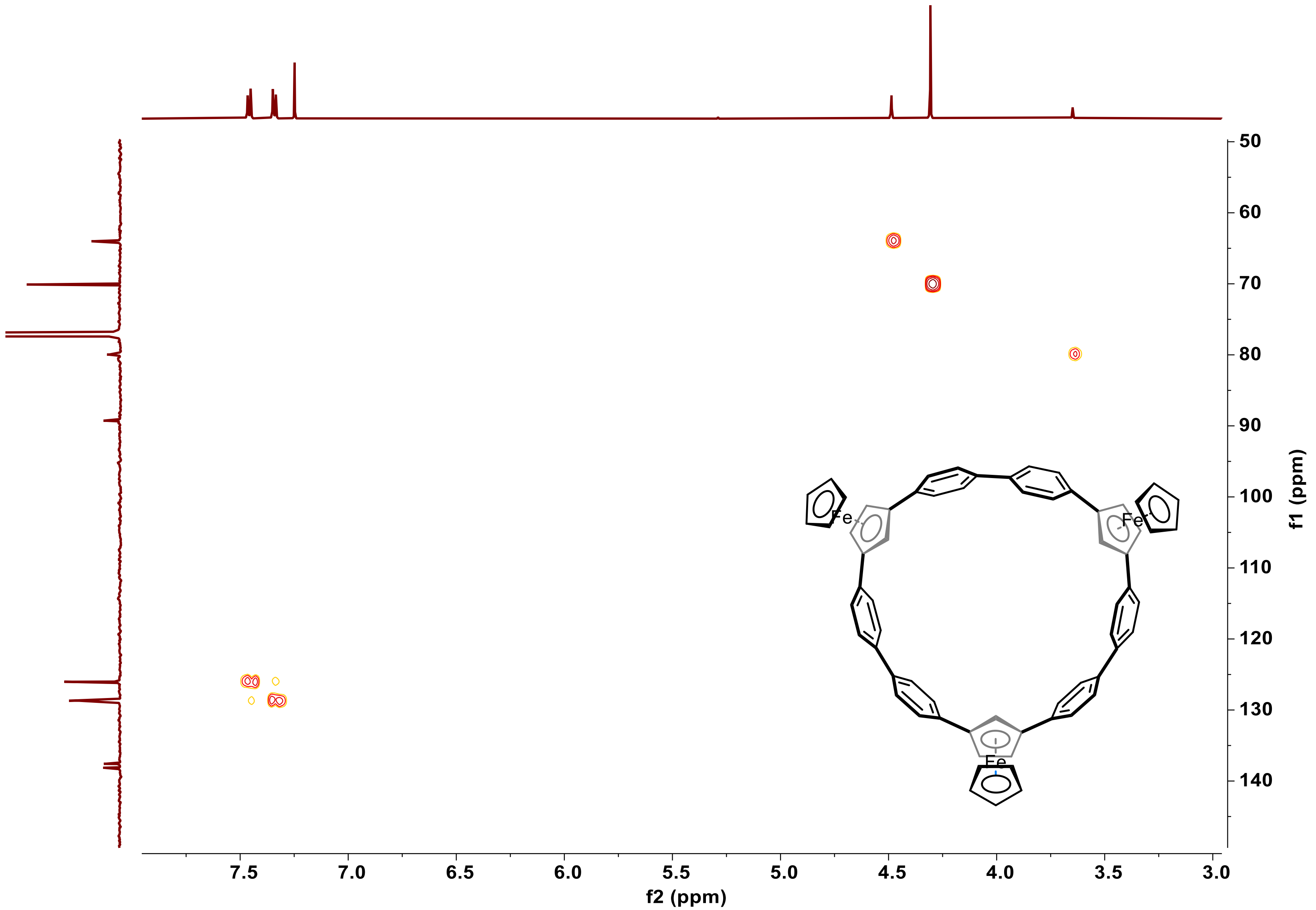

Figure S49. HMQC-spectrum of **A**$_3$ in $CDCl_3$ (500/126 MHz).

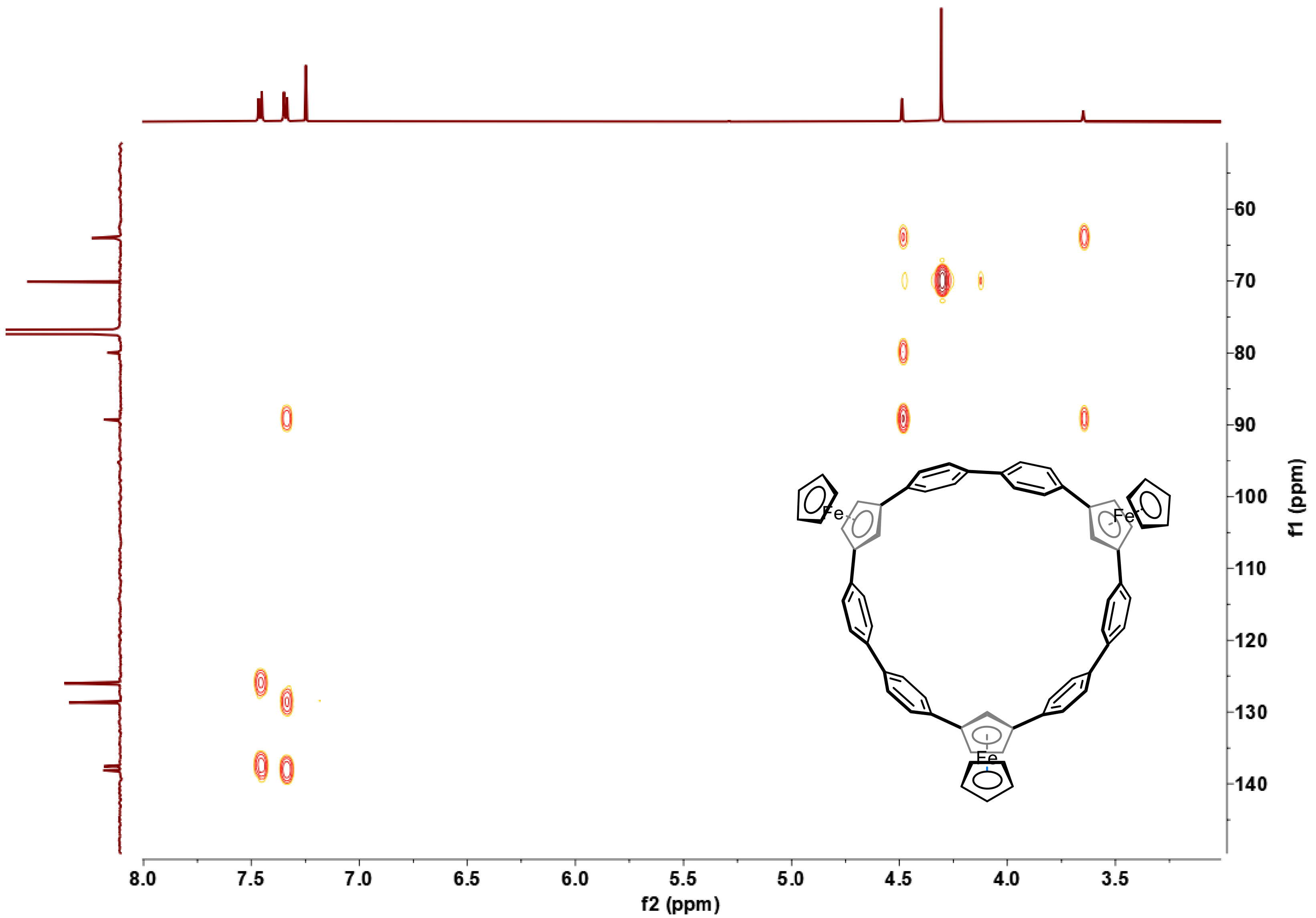

Figure S50. HMBC-spectrum of **A**$_3$ in $CDCl_3$ (500/126 MHz).

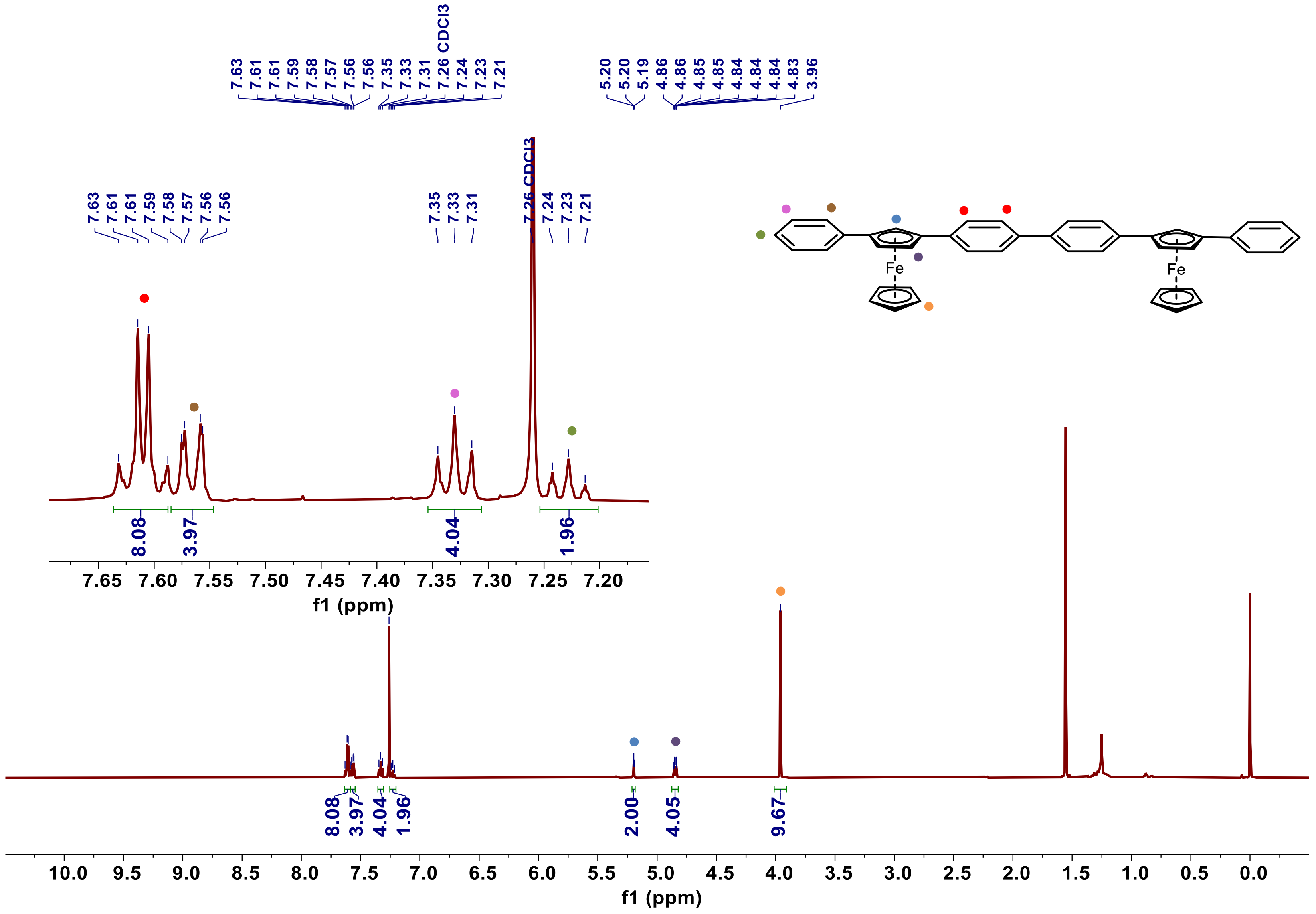

Figure S51. ^{1}H NMR-spectrum of **A_2** in $CDCl_3$ (600 MHz).

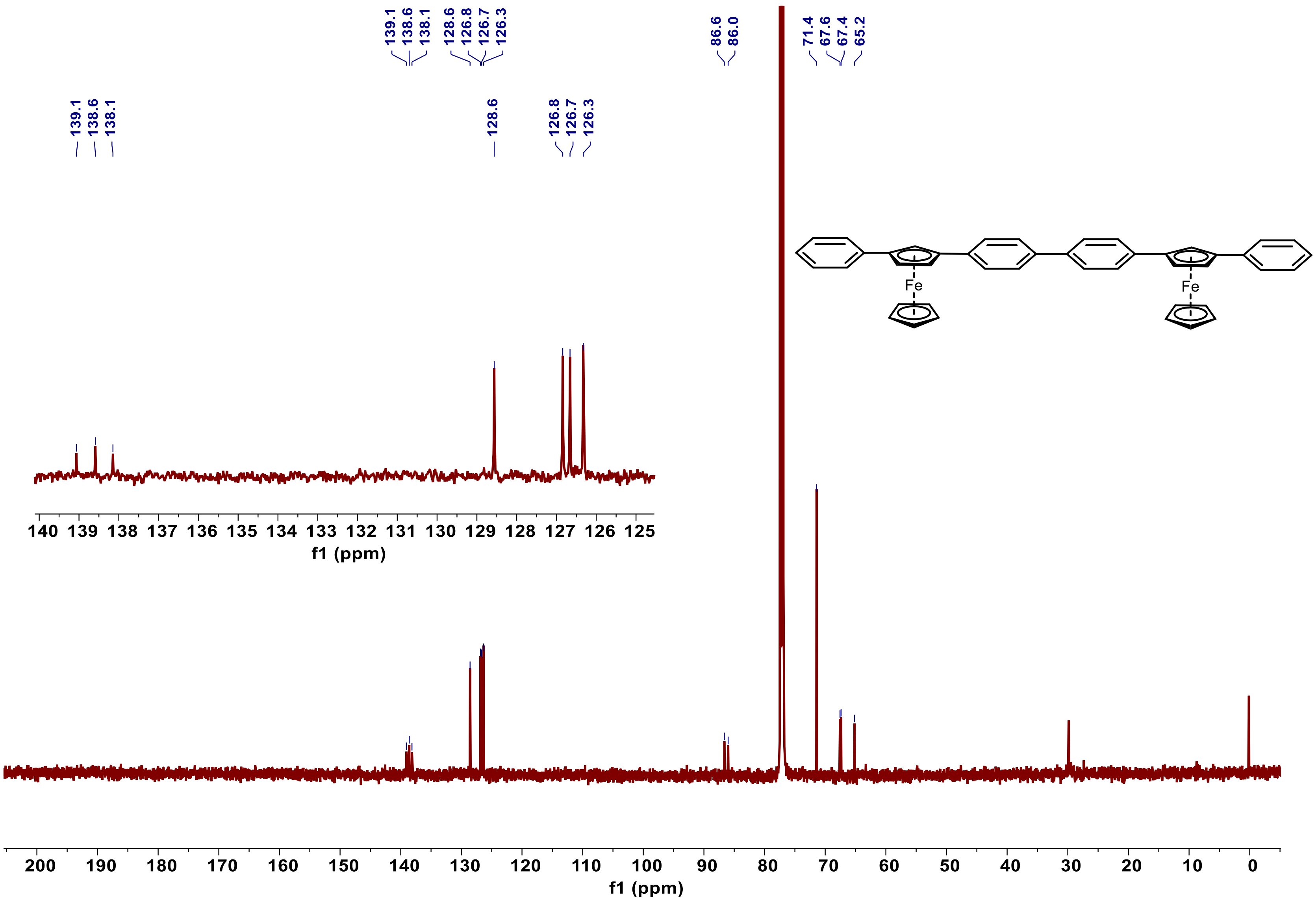

Figure S52. $^{13}C\{^{1}H\}$ NMR-spectrum of **A$_2$** in $CDCl_3$ (151 MHz).

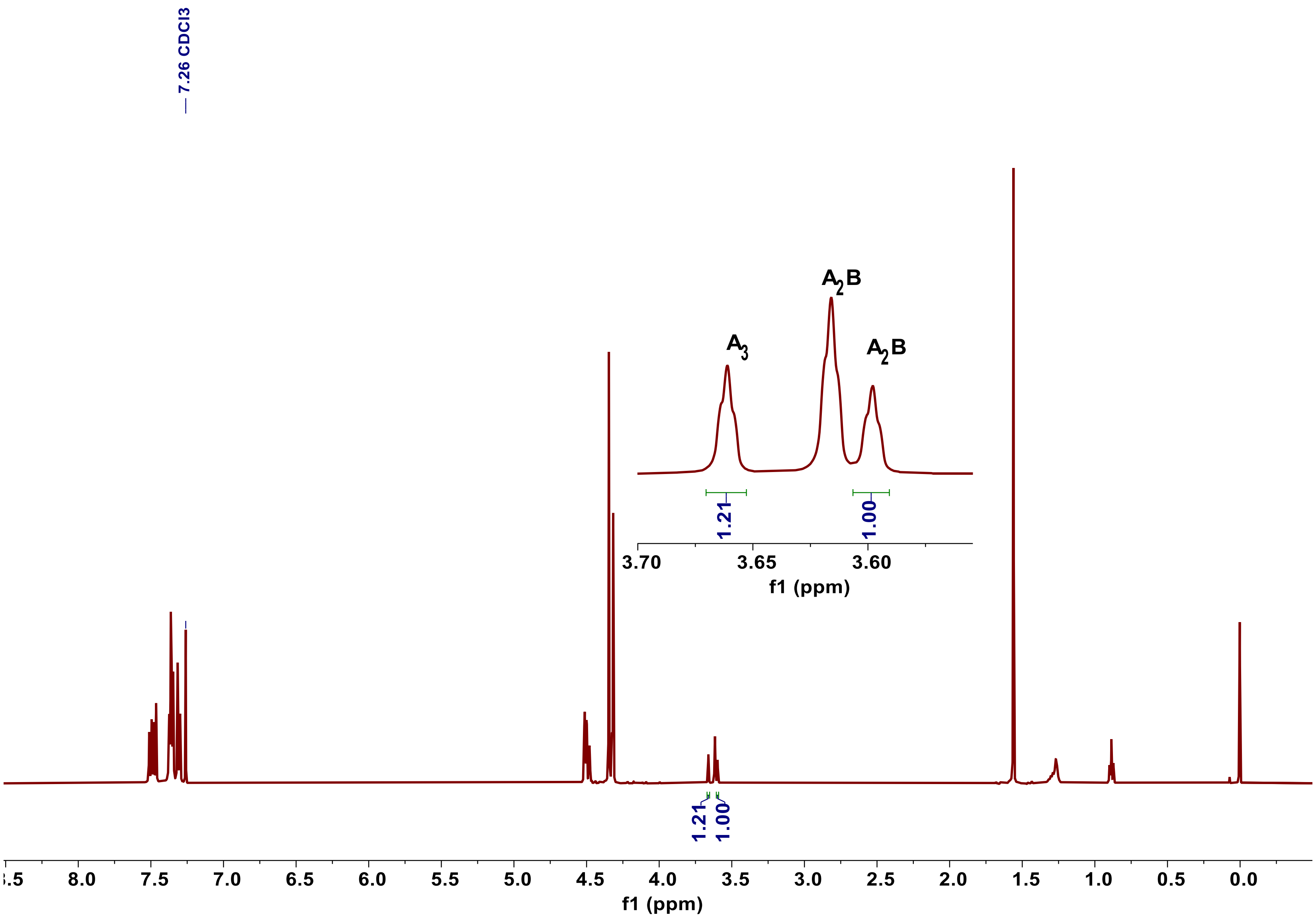

Figure S53. ^{1}H NMR analysis of the ratio of **A$_2$B** and **A$_3$**

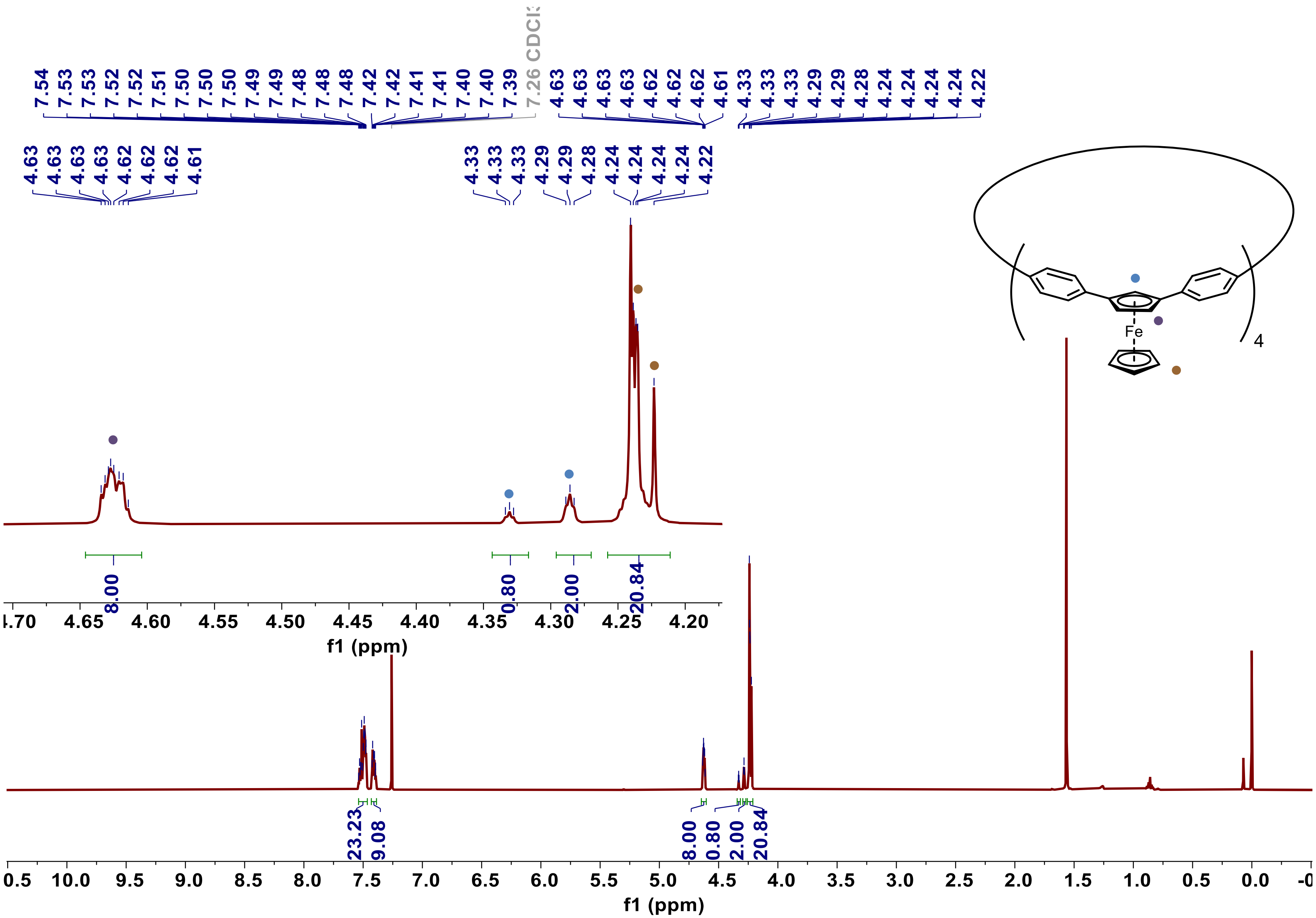

Figure S54. ^{1}H NMR of **Fc$_4$** in $CDCl_3$ (600 MHz).

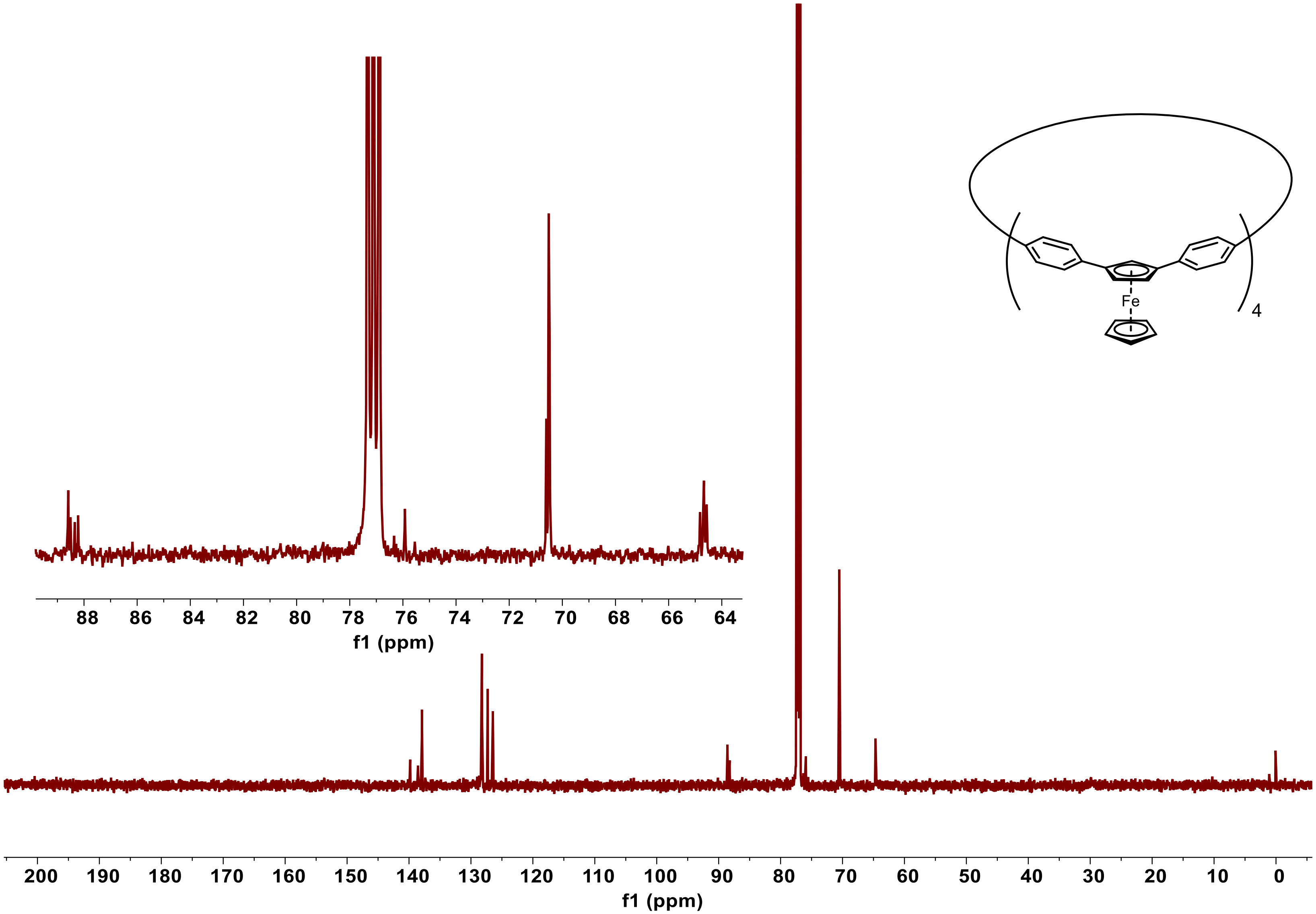

Figure S55. $^{13}C\{^{1}H\}$ NMR of **Fc4** in $CDCl_3$ (151 MHz).
